# Placental Inflammation Leads to Abnormal Embryonic Heart Development

**DOI:** 10.1161/CIRCULATIONAHA.122.061934

**Published:** 2022-12-09

**Authors:** Eleanor J. Ward, Serena Bert, Silvia Fanti, Kerri M. Malone, Robert T. Maughan, Christina Gkantsinikoudi, Fabrice Prin, Lia Karina Volpato, Anna Paula Piovezan, Gerard J. Graham, Neil P. Dufton, Mauro Perretti, Federica M. Marelli-Berg, Suchita Nadkarni

**Affiliations:** William Harvey Research Institute, Queen Mary University of London, Charterhouse Square, UK (E.J.W., S.B., S.F., C.G., N.P.D., M.P., F.M.M.-B., S.N.).; European Bioinformatics Institute, Wellcome Genome Campus, Hinxton, Cambridge, UK (K.M.M.).; National Heart and Lung Institute, Imperial College London, UK (R.T.M.).; Crick Advanced Light Microscopy Facility, the Francis Crick Institute, London, UK (F.P.).; Postgraduate Program in Health Science, University of Southern Catarina, Campus Pedra Branca, Palhoça, SC, Brazil (L.K.V., A.P.P.).; Institute of Infection, Immunity and Inflammation, University of Glasgow, UK (G.J.G.).

**Keywords:** fetus, heart defects, congenital, inflammation, macrophages, mothers, neutrophils, placenta

## Abstract

Placental heart development and embryonic heart development occur in parallel, and these organs have been proposed to exert reciprocal regulation during gestation. Poor placentation has been associated with congenital heart disease, an important cause of infant mortality. However, the mechanisms by which altered placental development can lead to congenital heart disease remain unresolved.

**Methods:**

In this study, we use an in vivo neutrophil-driven placental inflammation model through antibody depletion of maternal circulating neutrophils at key stages during time-mated murine pregnancy: embryonic days 4.5 and 7.5. Pregnant mice were culled at embryonic day 14.5 to assess placental and embryonic heart development. A combination of flow cytometry, histology, and bulk RNA sequencing was used to assess placental immune cell composition and tissue architecture. We also used flow cytometry and single-cell sequencing to assess embryonic cardiac immune cells at embryonic day 14.5 and histology and gene analyses to investigate embryonic heart structure and development. In some cases, offspring were culled at postnatal days 5 and 28 to assess any postnatal cardiac changes in immune cells, structure, and cardiac function, as measured by echocardiography.

**Results:**

In the present study, we show that neutrophil-driven placental inflammation leads to inadequate placental development and loss of barrier function. Consequently, placental inflammatory monocytes of maternal origin become capable of migration to the embryonic heart and alter the normal composition of resident cardiac macrophages and cardiac tissue structure. This cardiac impairment continues into postnatal life, hindering normal tissue architecture and function. Last, we show that tempering placental inflammation can prevent this fetal cardiac defect and is sufficient to promote normal cardiac function in postnatal life.

**Conclusions:**

Taken together, these observations provide a mechanistic paradigm whereby neutrophil-driven inflammation in pregnancy can preclude normal embryonic heart development as a direct consequence of poor placental development, which has major implications on cardiac function into adult life.

**Clinical Perspective**

**What is New?**

- Placental inflammation leads to loss of barrier function, which negatively affects embryonic heart development.
- Placental CCR2^+^ maternal inflammatory monocytes/macrophages impede resident cardiac fetal macrophage phenotype and function.
- Directly targeting placental tumor necrosis factor-α rescues defective cardiac development.

**What are the Clinical Implications?**

- There is potential for placental inflammation to be used as a diagnostic tool to evaluate fetal risk of congenital heart diseases.
- Targeted placental anti-inflammatory therapy offers an alternative clinical approach to current invasive in utero interventions.

**Editorial, see p 973**

Congenital heart diseases (CHDs) are an important cause of stillbirths, with ≈11% of stillbirths attributed to a type of CHD,^1^ and are associated with ≈35% of infant deaths.^2^ The heart is one of the first organs to develop in the human embryo, the first stages beginning by the end of the second week of pregnancy.^3^ In a series of coordinated developmental stages, the heart begins as a primitive heart tube, developing into the 4-chamber organ by the eighth week of pregnancy with a detectable heart rate.^4^ By the second trimester, the heart can start pumping blood around the fetus. The placenta is a specialized organ that acts as a tight barrier to regulate the transfer of oxygen and nutrients to the developing fetus while preventing passage of harmful pathogens and cells. Placental heart development and embryonic heart development occur in parallel, suggesting that the 2 organs influence the development of each other.^5^ Clinical studies suggest a strong association between placental dysfunction and CHDs,^6^ with poor trophoblast invasion and aberrant oxygen and nutrient transfer from the mother, leading to poor fetal cardiac development.^7^ This is compounded by evidence suggesting that women who have preeclampsia during their pregnancy have a significantly increased risk of their fetuses developing a CHD.^8^

The role of tissue-resident macrophages in promoting normal organogenesis is well established.^9^ At around embryonic day (E) 8.5, yolk sac–derived erythro-myeloid progenitors migrate to the developing embryo in a chemokine-dependent manner. These erythro-myeloid progenitors develop into premacrophages expressing CX_3_CR1, Kit, and CSFR1 and seed various organs, including brain, liver, and heart, where they persist into adulthood through a process of self-renewal.^10^ In the heart, CX_3_CR1^+^CCR2^−^ macrophages migrate from the yolk sac to promote angiogenesis, regulate coronary vascular development,^11^ and exert reparative functions in adult cardiac tissue.^12^ This contrasts with blood monocyte–derived CCR2^+^ macrophages, which promote inflammation in the adult heart.^12,13^

We previously demonstrated that our model of maternal neutrophil depletion during murine pregnancy indues a preeclampsia-like phenotype typified by abnormal placental development, including shallow invasion of trophoblasts into the decidua and subsequent remodeling of the spiral arteries.^14^ In the present study, we revisit this model and demonstrate that maternal neutrophil depletion promotes placental inflammation and a breakdown in the tight placental tissue barrier, both key features of preeclampsia placental phenotype.^15,16^ We go on to show that this neutrophil-driven placental inflammation (NDPI) model allows migration of inflammatory maternal monocytes to the embryonic heart, which, in turn, promotes abnormal fetal cardiac development with inadequate cardiac function in postnatal and adult life.

## Methods

The data that support the findings of this study are available from the corresponding author on reasonable request.

Clinical samples were approved by the institutional review board of the University of Southern Santa Catarina under 34681920.8.0000.5369. Animal studies were conducted with strict adherence to the home office guidelines (PPL P71E91C8E). A detailed description of the methods is available in the Supplemental Material.

Figure S13 provides flow cytometry gating strategy and antibody and primer information. All raw RNA sequencing data and single-cell sequencing data have been deposited on Figshare (https://figshare.com/s/8b13463311cf442e9d15, https://figshare.com/s/98321569e7f6a15aff65, and https://figshare.com/s/83bf3f8ba06d4884d827).

### Statistical Analyses

Statistical analyses were performed with Prism software (GraphPad, version 9). In all cases, data were tested for normality with the Kolmogorov-Smirnov test. For data comparing control with NDPI and that had equal variances, a 2-tailed Student *t* test was used. For unequal variance, the Welch *t* test was used, and the Mann-Whitney test was used for nonparametric comparison. For data comparing ≥3 groups, when data had equal variance, we conducted 1-way ANOVA with post hoc Bonferroni test. Comparison of data with unequal variance was tested with the Brown-Forsythe ANOVA, followed by the post hoc Dunnett test. Two-way ANOVA was used to determine statistical significance between groups and ≥2 cell populations. In all cases, values of *P*<0.05 were considered significant.

## Results

### A Neutrophil-Driven Model of Placental Inflammation

**Figure 1.**
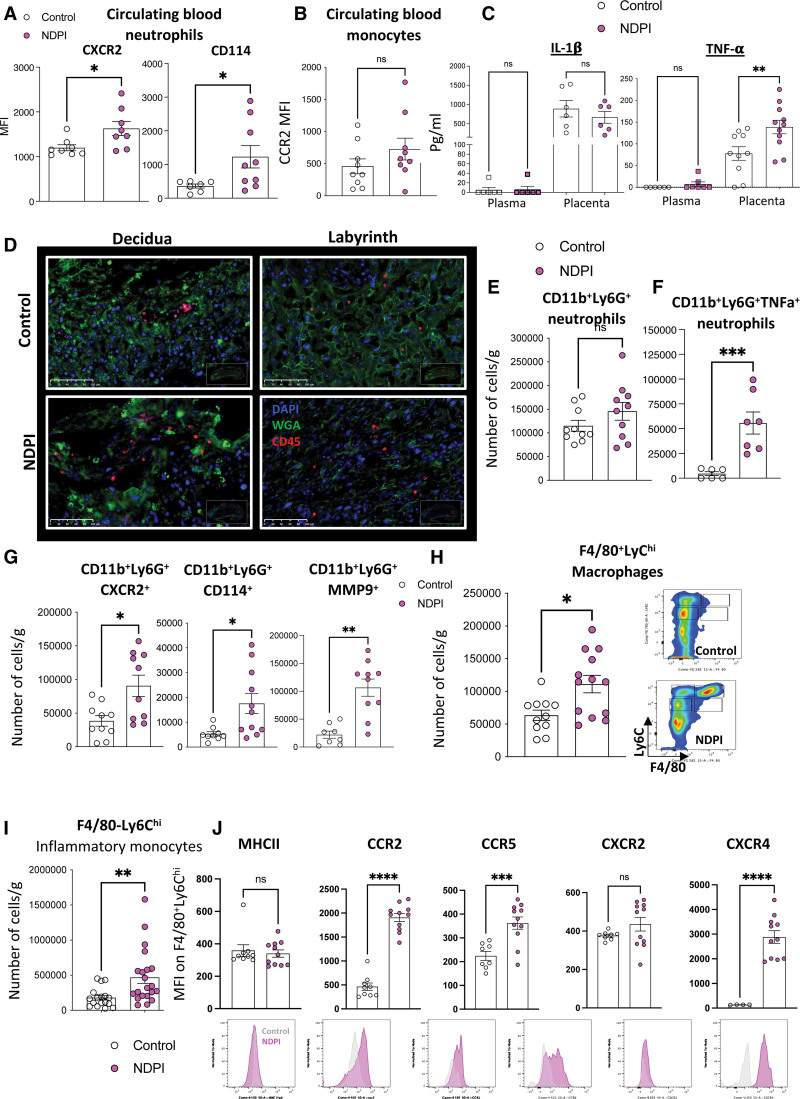
**An NDPI model.** Neutrophils were depleted at days 4.5 and 7.5 of pregnancy with αLy6G. Mice were euthanized at embryonic day E14.5 of pregnancy, and placentas were harvested to assess the structure and immune composition. Isotype control treated (referred to hereafter as control; white) and neutrophil depleted (referred to hereafter as neutrophil-driven placental inflammation [NDPI]; pink). **A**, Expression of activation markers by maternal blood neutrophils. **B**, CCR2 expression on maternal blood monocytes. **C**, ELISA showing the concentration of interleukin-1β (IL-1β) and tumor necrosis factor-α (TNF-α) in plasma and placenta digest supernatants. **D**, Immunofluorescent staining of placentas with CD45 (red) and wheat germ agglutinin (WGA; green) and cell nuclei with DAPI (blue). Expression is shown in the decidual layer (**left**) and labyrinth layer (**right**). **E** through **G**, Neutrophil subpopulations from placentas analyzed by flow cytometry expressed as absolute cell number per gram of tissue. **E**, CD11b^+^Ly6G^+^ neutrophils, (**F**) CD11b^+^Ly6G^+^ TNF-α^+^ neutrophils, and (**G**) neutrophils expressing CD11b^+^Ly6G^+^ CXCR2, CD11b^+^Ly6G^+^ CD114^+^, and CD11b^+^Ly6G^+^ MMP9^+^. **H**, Macrophages from placentas analyzed by flow cytometry expressed as absolute cell number per gram of tissue. **I**, Inflammatory monocytes from placentas analyzed by flow cytometry expressed as absolute cell number per gram of tissue. **J**, F4/80^−^Ly6C^hi^ populations from placentas were analyzed for the expression of major histocompatibility complex II (MHCII), CCR2, CXCR2, CCR5, and CCR6, expressed as median fluorescent intensity. Representative histograms are shown below the quantification (gray=control, pink=NDPI). Each symbol represents an individual mouse from different pregnancies, and statistical significance was tested by unpaired Student *t* test or Welch *t* test (**G** [CXCR2 and CD114] and **H**). In all cases, data are mean±SEM. MFI indicates median fluorescence intensity; MMP9, matrix metalloproteinase-9; and ns, not significant. *P≤0.05. **P≤0.01. ***P≤0.001. ****P≤0.0001.

After antibody depletion (anti-Ly6G, clone IA8), neutrophils return to the circulation within 72 hours,^17^ and these cells now present an activated, proinflammatory phenotype, characterized by high CXCR2 and CD114 (G-CSFR) expression. No differences were observed in circulating proinflammatory CCR2^+^ monocytes (Figure 1A). We next investigated the placental environment in more detail. Maternal neutrophil depletion resulted in smaller placentas and shallow trophoblast invasion (Figure S1B and S1C), coupled with an exaggerated tumor necrosis factor-α (TNF-α) placental concentration, but not the circulation (Figure 1C). There was no overall difference in total CD45^+^ leukocyte numbers (Figure 1D and Figure S1D) or in numbers of CD3^+^ T cells and natural killer cells (Figure S1E). Although placentas from neutrophil-depleted mothers displayed no overall difference in the number of neutrophils compared with their isotype control (referred to hereafter as control) counterparts (Figure 1E), placental neutrophils displayed an activated phenotype with a high expression of TNF-α, CXCR2, CD114 (granulocyte colony-stimulating factor receptor), and matrix metalloproteinase-9 (Figure 1F and 1G). Therefore, neutrophil depletion in pregnant mice induces NDPI.

NDPI was also defined by presence of activated F4/80^+^Ly6C^hi^ macrophages (Figure 1H), with no difference in the number of these macrophages making TNF-α between both groups (Figure S1F), suggesting that neutrophils are the likely source of increased TNF-α in NDPI. Intraplacental monocytes displayed an inflammatory phenotype compared with control (Figure 1I). These F4/80^−^Ly6C^hi^CCR2^+^ monocytes expressed higher levels of CCR2, CCR5, and CXCR4, but not major histocompatibility complex (MHC) II or CXCR2 (Figure 1J), compared with control. Lack of difference in total leukocyte numbers between placentas from control and NDPI pregnancies (Figure S1D), coupled with no overall change in the inflammatory status of circulating maternal monocytes, indicated that the phenotype of placental macrophages and monocytes is not attributable to an influx from the maternal circulation but rather to in situ activation. Thus, we hypothesized that TNF-α–producing neutrophils within the placental tissues regulate the phenotype of placental monocytes and macrophages. To challenge this hypothesis, we isolated placental neutrophils from control and NDPI pregnancies and cocultured them with naive splenic monocytes from nonpregnant age-matched mice. Cocultures induced higher proportions of inflammatory F4/80^+^Ly6C^hi^ macrophages expressing MHC II and CCR2 after monocyte exposure to NDPI neutrophils, mirroring the inflammatory phenotype observed in NDPI in vivo (Figure S1G and S1H).

### Neutrophil-Driven Inflammation Promotes a Breakdown in Placental Tissue Barrier

**Figure 2.**
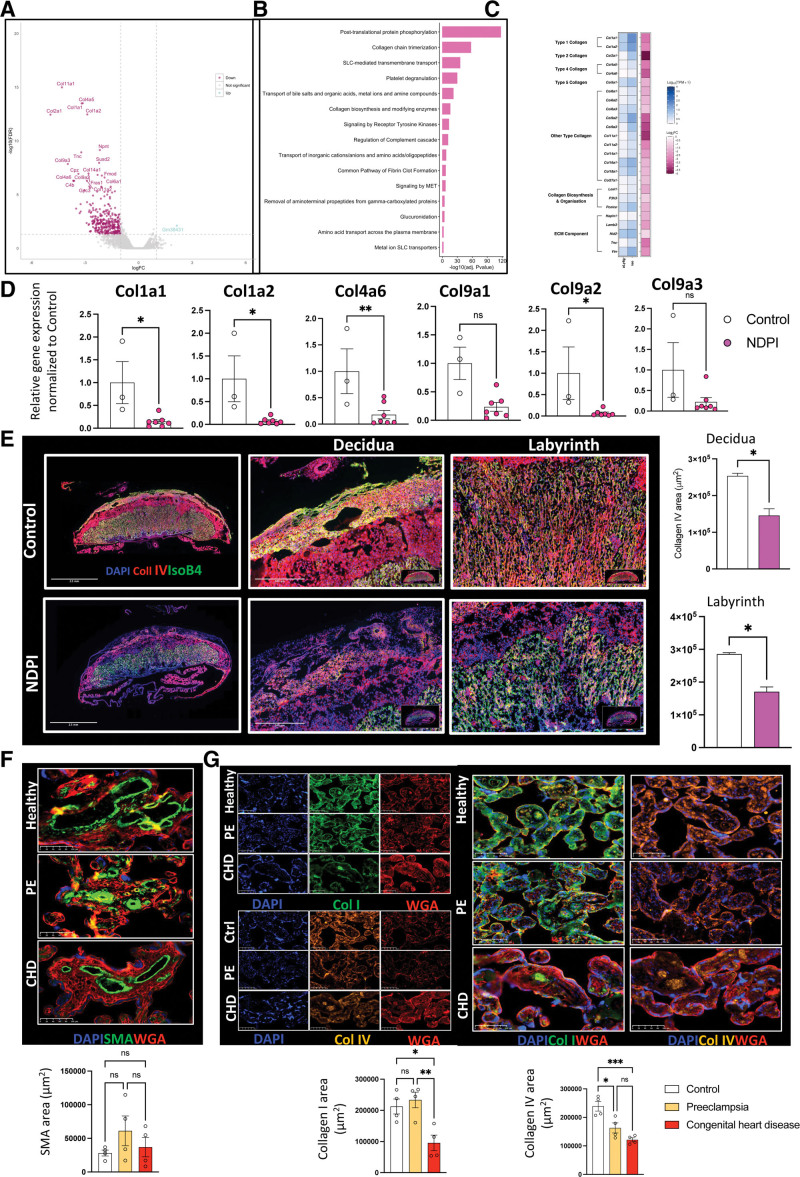
**Neutrophil-driven inflammation promotes a breakdown in the placental tissue barrier. A** through **E**, Neutrophils were depleted at days 4.5 and 7.5 of pregnancy with αLy6G. Mice were euthanized at E14.5 of pregnancy, and placentas were harvested. Control (white) and neutrophil-driven placental inflammation (NDPI; pink). **A** through **C**, RNA sequencing from placentas. **A**, Volcano plot showing differentially expressed genes in control vs NDPI placentas. **B**, Top 15 modulated pathways in placentas of NDPI mice compared with control. **C**, Heat map showing gene expression of extracellular matrix components of NDPI or control placentas. **D**, Reverse transcriptase–polymerase chain reaction showing the gene expression of Col1a1, Col1a2, Col4a6, Col5a1, Col9a1, Col9a2, and Col9a3. **E**, Immunofluorescent images of control or NDPI murine placentas stained with DAPI (blue), collagen (Col) IV (red), and isolectin B4 (red). Magnified images of the decidua and labyrinth of the placenta and graphs showing the quantification of Col IV staining. **F** and **G**, Immunofluorescent staining of term placentas from women with healthy, preeclamptic pregnancies or pregnancies carrying babies with congenital heart disease (CHD) for (**F**) smooth muscle actin (SMA; green), (**G**) Col I (green; **top**), and Col IV (orange; **bottom**). In all images, wheat germ agglutinin (WGA) staining is in red, and cell nuclei are stained with DAPI (blue). Quantification of area stained with SMA, Col I, and Col IV is shown below images. Each symbol represents an individual sample from different pregnancies, and statistical significance was tested by unpaired Student *t* test or Welch *t* test (**E**) or 1-way ANOVA (**F** and **G**). In all cases, data are mean±SEM. ns Indicates not significant. *P≤0.05. **P≤0.01. **P≤0.001.

To further investigate the features of NDPI, we undertook bulk RNA sequencing of E14.5 placental tissues from control and NDPI pregnancies. In NDPI, we observed downregulation of 329 genes, of 357 genes in total, to be significantly changed (false detection rate–corrected *P*<0.05; Figure 2A). Pathway analyses identified posttranslational protein phosphorylation and collagen trimerization as the top pathways (Figure 2B).

We focused our attention on collagen genes because these extracellular matrix components are important for placental tissue integrity.^18^ Further analyses revealed that of the 44 collagen genes within the mouse genome, 17 were significantly downregulated in NDPI (Figure S2A), including *Col1a1* and *Col1a2, Col2a1, Col4a6, Col9a2* and *Col9a3, Col11a1, Col11a2*, and *Col11a3* (Figure 2C and 2D). Immunofluorescent staining for 2 collagens (type I and IV) demonstrated lower expression of collagen I in the decidua of NDPI placentas but no difference in the labyrinth compared with control (Figure S2B). For collagen IV, significant reductions were quantified in both decidua and labyrinth of NDPI placentas (Figure 2E). Collagen IV is also required for the invasive properties of trophoblasts,^19^ suggesting that reduced collagen IV expression may explain the shallow trophoblast invasion displayed in NDPI settings.

We next sought to establish whether these collagen matrix features are present in human tissues in 2 types of pregnancy complications that affect placental development (Table). We assessed term placental tissue from preeclamptic pregnancies, chosen because of an activated neutrophil environment in these patients,^14,16^ and from pregnancies with fetuses that have CHD in the absence of maternal preeclampsia. Both were compared with normal, uncomplicated pregnancies. There were no significant changes in smooth muscle actin expression surrounding the maternal spiral arteries in all 3 placentas (Figure 2F). Collagen I from CHD placentas was significantly attenuated compared with healthy and preeclamptic placentas (Figure 2G), whereas collagen IV was significantly downregulated in both preeclamptic and CHD placentas compared with healthy placentas (Figure 2G). Together, these data indicate that NDPI has a negative functional impact on the placental support structure.

**Table.**
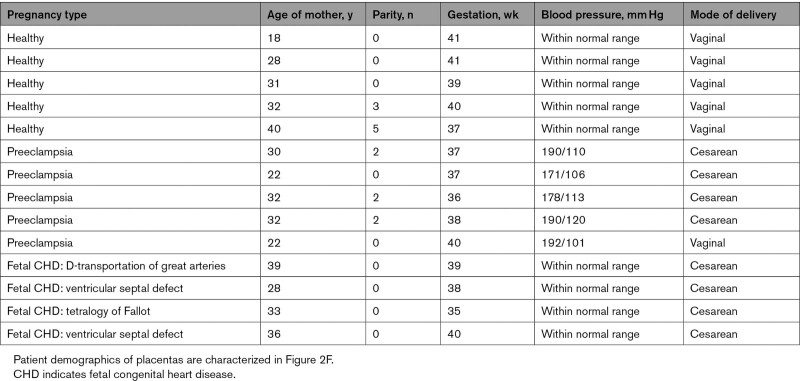
Demographics of Pregnant Patients From Whom Placental Tissue Was Assessed

### Placental Inflammation Leads to Poor Embryonic Cardiac Development

**Figure 3.**
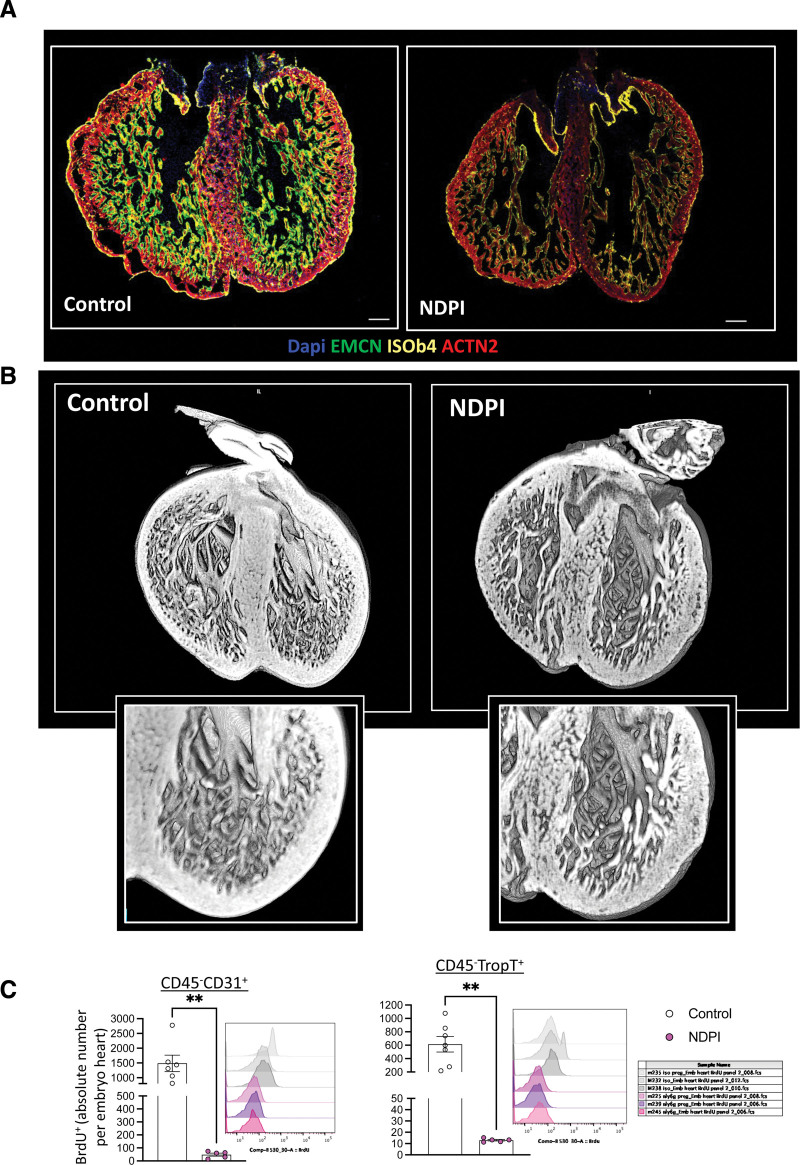
**Placental inflammation leads to poor embryonic cardiac development.** Neutrophils are depleted at days 4.5 and 7.5 of pregnancy with αLy6G. Mice were euthanized at E14.5 of pregnancy, and embryos were harvested. Control is in white; neutrophil-driven placental inflammation (NDPI) is in pink. **A**, Immunofluorescent images of cross section of embryonic day (E) 14.5 embryo heart. Sections were stained with DAPI (blue), endomucin (EMCN; green), isolectin B4 (ISOb4; yellow), and actinin-2 (ACTN2; red). **B**, High-resolution episcopic microscopy images of E14.5 hearts. **C**,Quantification of the in vivo uptake of BrdU into CD45^−^CD31^+^ endothelial cells and CD45^−^troponin-T^+^ cardiomyocytes in embryo hearts. Each symbol represents an individual mouse, and statistical significance was tested by unpaired Student *t* test. In all cases, data are mean±SEM. ns Indicates not significant. *P≤0.05. **P≤0.01. ***P≤0.001. ****P≤0.0001.

We next investigated the association between poor placentation and cardiac development in utero. Initial observations revealed abnormal heart development in the gross structure of embryonic hearts at E14.5 from NDPI pregnancies, with significantly thinner compact myocardium within the left ventricle (LV) compared with control, coupled with attenuated endomucin (endocardial cell marker) staining within the total heart area, indicating poor vascularization (Figure 3A and Figure S3A–S3C). However, this defect appeared to be restricted to the endocardium because we observed no difference in expression on epicardium-specific WT-1 (Figure S3D) or in gene expression of epicardium-specific *Tcf21* and *Sema3d*. We observed that NDPI embryonic hearts have impaired transforming growth factor-β activity, displaying reduced phosphorylation of SMAD3 (Figure S4A) and diminished expression of downstream TGF-SMAD3 target, TAGLN (transgelin), at E14.5 (Figure S4B). SMAD3 is required for the activation of TAGLN,^20^ suggesting a potential defect in smooth muscle cell formation in NDPI E14.5 hearts. Moreover, *Tagln*-Cre:*Tgfbr2* knockout mice display a less compact and thinner LV wall.^21^

Between E9.0 and E9.5, primitive cardiomyocytes within the ventricular wall form finger-like projections called trabeculae that are lined by endocardial cells. As cardiac development progresses, ventricles undergo a switch from a mostly trabecular to a compacted state in which cardiomyocytes compact and increase ventricular wall thickness, which is essential for normal heart function. Dysregulation of this switch can cause hypertrabeculation, leading to a congenital cardiomyopathy called LV noncompaction (LVNC).^22^ Three-dimensional imaging with high-resolution episcopic microscopy revealed increased trabeculation of the ventricular walls of hearts from NDPI embryos compared with controls in terms of both number and length (Figure 3B and Figure S5A), indicative of an LVNC-like phenotype. Recent studies have demonstrated that dysregulation of coronary endothelial cells promotes LVNC.^23^ With this in mind, we interrogated the status of the proliferation of endothelial cells within the embryonic heart. Flow cytometric analyses revealed reduced numbers of proliferating CD31^+^ endothelial cells in NDPI embryonic hearts compared with controls as assessed by in vivo BrdU incorporation (Figure 3C, left), as well as a significant reduction in phospho-histone H3 staining as indicated by immunofluorescence (Figure S5B). We also observed attenuated expression of key regulators of angiogenesis, including intercellular adhesion molecule-1, vascular cell adhesion molecule-1, endoglin, and thrombospondin (Figure S5C). These data indicate that NDPI impedes normal embryonic heart development by hindering embryonic heart vascularization. This attenuation in endothelial cell proliferation was accompanied by a significant downregulation in the number of proliferating troponin-T^+^ cardiomyocytes (Figure 3C, right), adding further support that embryonic hearts from NDPI pregnancies develop abnormally.

### CCR2 Driven Accumulation of Maternal Proinflammatory Leukocytes in Embryonic Hearts of NDPI Pregnancies

**Figure 4.**
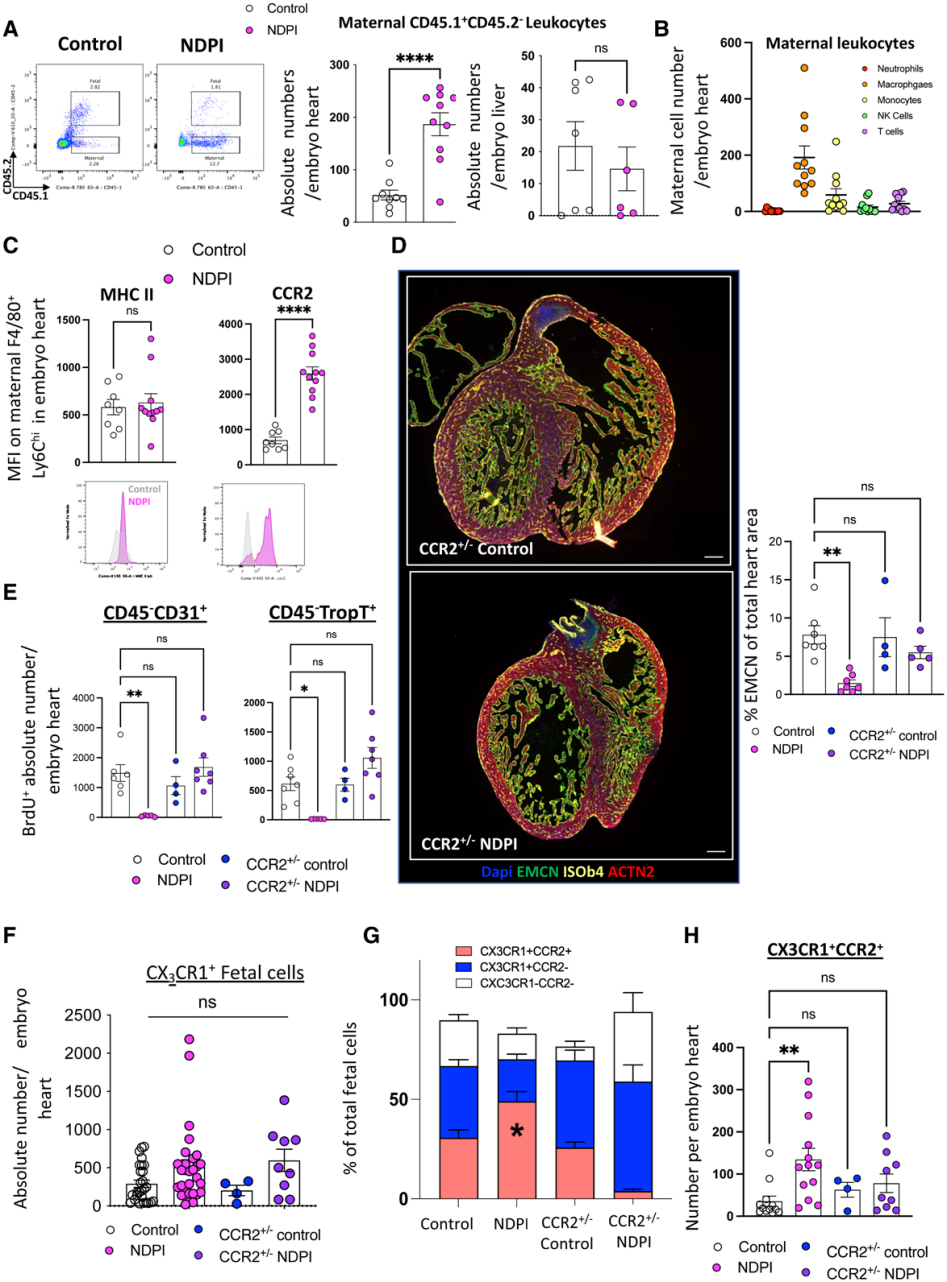
**CCR2-driven accumulation of maternal proinflammatory leukocytes in embryonic hearts of NDPI pregnancies.** Neutrophils were depleted at days 4.5 and 7.5 of pregnancy with αLy6G. Mice were euthanized at embryonic day (E)14.5 of pregnancy, and hearts were dissected from harvested embryos. Control is shown in white, and neutrophil depleted (neutrophil-driven placental inflammation [NDPI]) is shown in pink. **A**, Flow cytometry plots of leukocytes in E14.5 fetal hearts and NDPI pregnancies; CD45.1^+^ CD45.2^−^ cells are of maternal origin and CD45.1^+^CD45.2^+^ cells are of fetal origin. Graphs show quantification of number of maternal cells per embryo heart or embryo liver. **B**, Flow cytometry quantification of different maternal leukocyte subsets in embryo hearts. **C**, Median fluorescent intensity of major histocompatibility complex II (MHCII) and CCR2 on maternal F4/80^+^Ly6Chi cells found in embryo hearts. **Bottom**, Representative histograms. **D**, Immunofluorescent images of cross section of E14.5 embryo heart from CCR2^+/−^control and CCR2^+/−^NDPI pregnancies. Sections were stained with DAPI (blue), endomucin (EMCN; green), isolectin B4 (ISOb4; yellow), and actinin-2 (ACTN2; red). Graph indicates quantification of endomucin staining of embryonic hearts expressed as percent of total heart area. **E**, Quantification of the in vivo uptake of BrdU into CD45^−^CD31^+^ endothelial cells and CD45^−^troponin-T^+^ cardiomyocytes in embryo hearts from CCR2^+/−^control and CCR2^+/−^NDPI pregnancies compared with control. **F**, Quantification of absolute numbers of CX_3_CR1 fetal cells in E14.5 embryonic hearts from control, NDPI, CCR2^+/−^control, and CCR2^+/−^NDPI pregnancies. **G**, Proportion of CX_3_CR1^+^CCR2^+^ and CX_3_CR1^+^CCR2^−^ fetal cells in E14.5 embryonic hearts from control, NDPI, CCR2^+/−^control, and CCR2^+/−^NDPI pregnancies. **H**, Absolute number of CX_3_CR1^+^CCR2^+^ fetal cells in E14.5 embryonic hearts from control, NDPI, CCR2^+/−^control, and CCR2^+/−^NDPI pregnancies. Each symbol represents an individual mouse, and statistical significance was tested by unpaired Student *t* test. In all cases, data are mean±SEM. MFI indicates median fluorescence intensity; and ns, not significant. *P≤0.05. **P≤0.01. ***P≤0.001. ****P≤0.0001 (**A–C**): 1-way ANOVA with Dunnett multiple-comparison test compared with control (**D–F** and **H**); Brown-Forsythe ANOVA test with Dunnett multiple-comparison test (**E**, troponin-T); or 2-ANOVA comparing means of CCR2^+^ or CCR2^−^ percent between mouse groups with the Tukey multiple-comparison test (**G**).

To test whether immune cells from inflamed placentas have the capacity to directly shape embryonic heart development, we used the CD45.1/CD45.2 system, which allows discrimination of immune cells of maternal or fetal origin (see Methods). Maternal cells were identified as CD45.1^+^CD45.2^−^ (referred to hereafter as maternal), whereas fetal cells were identified as CD45.1^+^CD45.2^+^ (referred to hereafter as fetal). There was a 4-fold increase in the absolute number of maternal cells in the NDPI embryonic hearts compared with control, with no significant differences observed in maternal cells in the fetal liver (Figure 4A). The detection of maternal leukocytes in NDPI embryonic hearts was confirmed through adoptive transfer of GFP^+^ cells into the maternal circulation (see Methods). Both flow cytometry and immunofluorescence revealed a significant number of CD45^+^GFP^+^ in the placenta fetal layers and within in the embryonic hearts from NDPI but not control pregnancies (Figure S6A and 6B). These data were validated by the increased leakage of FITC dextran in NDPI placentas compared with their control counterparts (Figure S6C) and support the hypothesis that exaggerated placental inflammation promotes a breakdown in placental tissue barrier.

Phenotypic analyses of maternal leukocytes within the embryonic hearts from NDPI pregnancies revealed the presence of T cells, natural killer cells, neutrophils, and monocytes, with the predominant maternal cell type being F4/80^+^Ly6C^hi^ macrophages (Figure 4B), with no difference in MHC II expression between control and NDPI pregnancies but significantly increased expression of the proinflammatory chemokine receptors CCR2 (Figure 4C), CCR5, and CXCR4 (Figure S7A). This chemokine receptor expression profile was akin to that of placental inflammatory F4/80^−^Ly6C^hi^ monocytes from NDPI pregnancies. In line with our GFP (green fluorescent protein) cell tracking experiments, these data show that maternal cells migrate across the placental barrier and are recruited to the embryo heart. Quantitative polymerase chain reaction analyses revealed significant increases in gene expression for both CCL3 and CCL4, ligands for CCR5, with a reciprocal attenuation in CXCL12 (Figure S7B) in embryonic hearts from NDPI pregnancies compared with control. These changes were organ specific; no differences were observed in embryonic livers (Figure S7C), suggesting targeted migration of maternal cells specifically to the embryonic heart. There was a significant augmentation in both interleukin (IL)-6 and IL-1β in NDPI embryonic hearts but not TNF-α or IL-10 (Figure S7D). Intracellular flow cytometry identified a significant upregulation in the absolute number of maternal leukocytes, fetal leukocytes, and nonleukocytes expressing IL-1β in NDPI hearts and only maternal macrophages and nonleukocytes expressing higher levels of IL-6 (Figure S7E). Together, these data indicate a maternal proinflammatory CCR2-driven environment within the embryonic heart interferes with normal development of the organ.

To mechanistically challenge our hypothesis that maternal CCR2^+^ monocytes cause aberrant heart development, we investigated the impact of maternal monocyte CCR2 deletion on embryonic heart development in our NDPI model. CCR2^−/^^−^ females on a B6 background were mated with Balb/C (CCR2^+/+^) males, and pregnancies were treated as outlined in Figure S1, with placental tissue, embryos, and offspring resulting from these pregnancies being CCR2^+/^^−^control or CCR2^+/^^−^NDPI. Initial analyses revealed that CCR2^+/^^−^NDPI placentas were of comparable weight compared with control (Figure S8A). TNF-α levels from placentas from CCR2^+/^^−^control and CCR2^+/^^−^NDPI pregnancies were similar to levels from control placentas (Figure S8B). The numbers of F4/80^+^Ly6C^hi^ inflammatory macrophages and F4/80^−^Ly6C^hi^ monocytes from CCR2^+/^^−^control and CCR2^+/^^−^NDPI placentas were analogous to those of control placentas (Figure S8C and S8D). This attenuation in inflammation in CCR2^+/^^−^NDPI placentas suggests that placental inflammation in NDPI pregnancies is maternally driven.

We next sought to look at the development of control and NDPI embryonic hearts. Reverse transcriptase–quantitative polymerase chain reaction analyses were carried out on key genes involved in embryonic heart development. There was a significant downregulation gene expression in NDPI embryonic hearts compared with control in the following genes: *Gata6* (regulates cardiomyocyte proliferation together with *Gata4*^24^); *Mef2c* (required for normal endocardium formation^25^); *Hey2* (involved in normal ventricular wall and myocyte development ^26^); *Loxl2* (required for maturation of extracellular matrix and normal ventricular septation during development^27^); *Myh6* and *Myh7* (required for cardiomyocyte contractility^28^); *Nppa* (restricted during development to the trabecular myocardium and required for ventricular formation^29^); and *Yap1* (required for vascular wall development through smooth muscle proliferation and cardiomyocyte proliferation^30^; Figure S8E). Given that we observed a LVNC-like phenotype, the downregulation in *Nppa* was surprising. However, *Nppa* is required for normal ventricular formation during cardiac development.^29^ Thus, the downregulation in *Nppa* could account for the ventricular defects that we also observed in our NDPI embryo hearts. Furthermore, recent data suggest that downregulation in the *Myh7* gene is associated with LVNC.^31^ Thus, the downregulation in *Myh7* expression in NDPI embryonic hearts could account for the LVNC-like phenotype.

There were significant increases in gene expression of *Gata4, Meft2c, Hey2*, and *Nppa* in both CCR2^+/^^−^control and CCR2^+/^^−^NDPI embryonic hearts compared with controls (Figure S8E). Immunofluorescence revealed normal development with endomucin expression from both CCR2^+/^^−^control and CCR2^+/^^−^NDPI embryonic hearts at levels comparable to those of control (Figure 3A and 4D), as well as proliferation of both CD31^+^ endothelial cells and troponin-T^+^ cardiomyocytes (Figure 4E).

We next focused on resident fetal cardiac macrophages, which are CX_3_CR1^+^ and originate from the yolk sac. These comprise 2 populations: the CCR2^−^ macrophage population that exerts proangiogenic functions during cardiac development^32,33^ and the CCR2^+^ macrophage population that has a short life span within the embryonic and neonatal heart, with currently unknown function.^33^ We found no difference in absolute CX_3_CR1^+^ numbers between control and NDPI pregnancies or between CCR2^+/^^−^control and CCR2^+/^^−^NDPI (Figure 4F). However, we observed an increased inflammatory CX3CR1^+^CCR2^+^ phenotype in NDPI embryonic hearts compared with control in terms of both proportion and absolute number of macrophages per heart (Figure 4G and 4H). This coincided with a 2-fold decrease in the number of proliferating resident fetal CCR2^−^ macrophages (Figure S8F). These data highlight that a skew toward a CX3CR1^+^CCR2^+^ heart resident fetal macrophage phenotype may underlie the dysregulated embryonic heart development observed in NDPI pregnancies.

**Figure 5.**
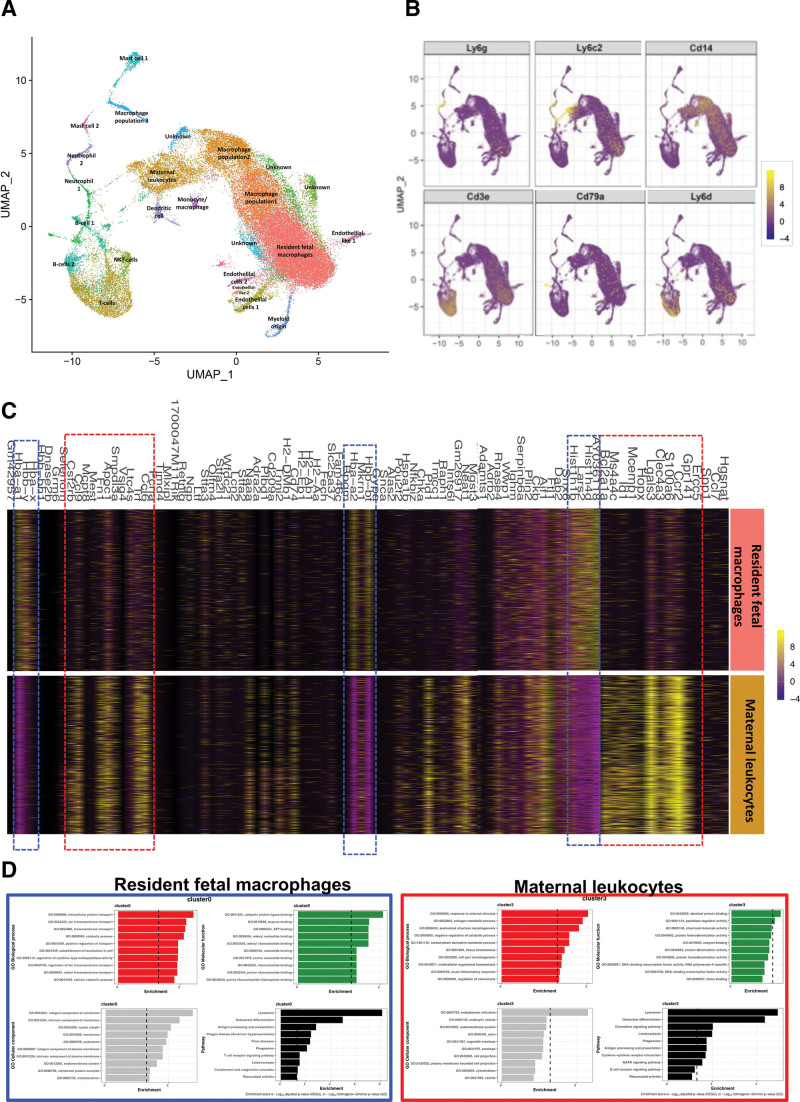
**Single-cell sequencing of leukocyte s from E14.5 embryo hearts.** Neutrophils were depleted at days 4.5 and 7.5 of pregnancy with αLy6G. Mice were sacrificed at embryonic day (E) 14.5 of pregnancy, and hearts were dissected from harvested embryos from control or NDPI pregnancies. CD45^+^ cells were isolated from heart single cell suspensions with CD45 preeclamptic and antipreeclamptic microbeads. Single-cell sequencing was performed on the isolated cells. **A**, Uniform Manifold Approximation and Projection showing cell clusters found in hearts of fetuses from control and NDPI pregnancies. **B**, Feature plots showing expression of key genes within clusters. **C**, Heart map analyses comparing gene expression between resident fetal macrophage and maternal leukocyte clusters. **D**, Enrichment analysis of Gene Ontology (GO) terms and pathways for differentially expressed genes. GO analysis including biological process, cellular component, and molecular function. Pathway analysis based on the KEGG database.

To gain a deeper understanding of leukocyte composition and phenotype in the embryonic hearts, we undertook single-cell RNA sequencing. CD45^+^ leukocytes were isolated from E14.5 embryonic hearts from control and NDPI pregnancies. Uniform Manifold Approximation and Projection analyses revealed heterogeneous leukocyte populations with a mixture of both myeloid and lymphoid cells present (Figure 5A and 5B and Figure S9 and S10). Further analyses of the clusters revealed resident fetal macrophage and maternal leukocyte clusters, expressing distinct genes. For example, resident fetal macrophages expressed many hemoglobin genes, including *Hbb-y, Hba-x, Hbb-bt*, and *Hba-a2*, which have been shown to be highly expressed in yolk sac–derived macrophages^34,35^ (Figure 5C, blue dotted lines). Genes associated with maternal leukocytes cluster appeared to be associated with adult macrophages, including *Ccr2, Lgals3, S100A6, Hopx, Ms4a4c, Clec4a3, Ccl6*, and *Ccl9* (Figure 5C, red dotted lines). Enrichment analyses also revealed distinct pathways between the resident fetal macrophage and maternal leukocyte clusters. GO (Gene Ontology) Biological Processes analyses of resident fetal macrophages revealed pathways involved in intracellular protein transport and ion transmembrane transport, with the top pathway hit being lysosome (Figure 5D, left). Indeed, evidence suggests that yolk sac macrophage lysosomal activity may be important in regulating fetal testis vascularization and morphogenesis,^36^ which may also have important implications in the vascularization of the developing heart. Enrichment analyses of the maternal leukocyte cluster revealed that GO Biological Processes associated with adult macrophages, including carbohydrate and collagen metabolism; macrophage-derived collagen has recently been demonstrated to contribute directly to cardiac fibrosis after injury^37^ (Figure 5D, right). Taken together, our single-cell RNA sequencing data suggest that a heterogeneous leukocyte population exists within the developing heart and that distinct gene clusters and pathways distinguish between resident fetal macrophages and maternal leukocytes.

### Impaired Cardiac Development From NDPI Pregnancies Persists Into Postnatal Life

We next investigated whether the abnormal embryonic heart development detailed at E14.5 continues into postnatal life, assessed at postnatal day (P) 5 and P28 (adult).

Offspring from NDPI pregnancies at P5 had smaller body weight and a higher heart:body weight ratio compared with their control counterparts (Figure S11A). We observed an ≈90% reduction in maternal cells within P5 hearts (Figure S11B). No significant difference was seen in the absolute number of resident CX_3_CR1^+^ leukocytes (Figure S11C), yet the proportion of CX_3_CR1^+^CCR2^−^ continued to be significantly lower in offspring hearts from NDPI pregnancies compared with control (Figure S11D). Because we observed an LVNC-like phenotype in embryo hearts from NDPI pregnancies at E14.5 (Figure 3), we assessed the cardiac architecture of P5 hearts from both groups, staining cross sections with DAPI, wheat germ agglutinin (for general architecture), and endomucin. Cardiac tissue of P5 hearts of offspring of NDPI pregnancies displayed hypertrabeculation, with a less compact endocardial structure coupled with a significant reduction in endomucin staining (Figure S11E), accompanied by a significant reduction in the number of CD31^+^ endothelial cells (Figure S11F). Postnatally, hearts from CCR2^+/^^−^NDPI offspring resembled control P5 hearts (Figure S11G), suggesting that the maternal CCR2^+^ leukocytes drive the defect in endocardial structure.

**Figure 6.**
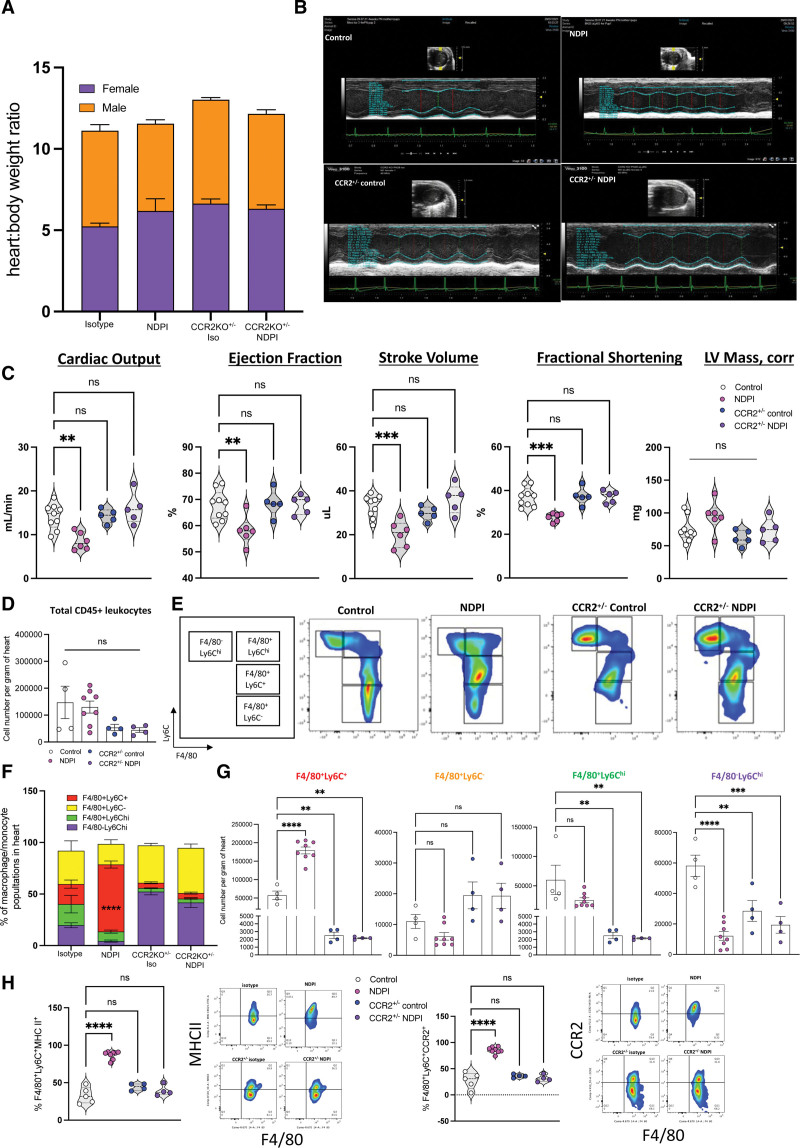
**Aberrant embryonic cardiac development from NDPI pregnancies continues into postnatal life.** Neutrophils were depleted at days 4.5 and 7.5 of pregnancy with αLy6G. Offspring of these dams were euthanized at postnatal day (P) 5 or P28 as indicated, and heart structure and immune composition were assessed. Offspring from control (white) and neutrophil-driven placental inflammation (NDPI; pink). **A**, P28 offspring body weights in grams and heart:body weight ratio from control, NDPI, CCR2^+/−^control, and CCR2^+/−^NDPI pregnancies. **B**, Representative echocardiography plots of P28 hearts of offspring from control, NDPI, CCR2^+/−^control, and CCR2^+/−^NDPI pregnancies. **C**,Graphs showing quantification of heart parameters determined by echocardiography. **D**, Flow cytometric quantification of total CD45^+^ leukocytes from P28 offspring hearts of control, NDPI, CCR2^+/−^control, and CCR2^+/−^NDPI pregnancies. **E**, Flow cytometric analyses of macrophage and monocyte populations as determined by F4/80 and Ly6C staining in P28 offspring hearts from control, NDPI, CCR2^+/−^control, and CCR2^+/−^NDPI pregnancies. **F**, Proportion of macrophage and monocyte populations shown in **E** in P28 offspring hearts from control, NDPI, CCR2^+/−^control, and CCR2^+/−^NDPI pregnancies. **G**, Absolute number per gram of heart tissue of monocyte and macrophage populations shown in **E** in P28 offspring hearts from control, NDPI, CCR2^+/−^control, and CCR2^+/−^NDPI pregnancies. **H**, Percentage of F4/80^+^Ly6C^+^ MHCII^+^ (**left**) and F4/80^+^Ly6C^+^ CCR2^+^ in P28 offspring hearts from control, NDPI, CCR2^+/−^control, and CCR2^+/−^NDPI pregnancies. Each symbol represents an individual mouse, and statistical significance was tested by 1-way ANOVA with the Dunnett multiple-comparison test or Brown-Forsythe ANOVA test with Dunnett post hoc comparison (**G**, F4/80^+^Ly6C^+^) compared with control. In all cases, data are mean±SEM. LV indicates left ventricular; and ns, not significant. *P≤0.05. **P≤0.01. ***P≤0.001. ****P≤0.0001.

No difference in heart:body weight ratios among all 4 mouse groups was observed regardless of the sex of the mouse (Figure 6A) at P28. Echocardiography of male and female adult offspring from NDPI pregnancies revealed a significant attenuation of cardiac output, stroke volume, ejection fraction, and fractional shortening, with no difference in LV mass, compared with control, whereas both CCR2^+/^^−^control and CCR2^+/^^−^NDPI offspring displayed normal cardiac function (Figure 6B and 6C). We next investigated the phenotype of cardiac macrophages in adult offspring hearts. No overall difference was observed in the total number of CD45^+^ leukocytes within the cardiac tissue across all 4 groups (Figure 6D). We assessed monocyte and macrophage subsets with respect to F4/80 and Ly6C, namely 3 macrophage populations, F4/80^+^Ly6C^+^, F4/80^+^Ly6C^hi^, and F4/80^+^Ly6C, as well as the monocyte population, F4/80^−^Ly6C^hi^. The proportion and number of F4/80^+^Ly6C^+^ macrophages were significantly increased in P28 hearts of NDPI offspring (Figure 6F and 6G), coupled with a significant proportion of this subset expressing MHC II and CCR2 (Figure 6H). We also observed a significant attenuation in the number of F4/80^+^Ly6C^+^, F4/80^+^Ly6C^hi^, and F4/80^−^Ly6C^hi^ populations in both CCR2^+/^^−^control and CCR2^+/^^−^NDPI offspring hearts compared with control (Figure 6G), adding support that this chemokine receptor is important in maintaining a proinflammatory environment within the cardiac tissue.^38^

### Quelling Placental Inflammation Prevents Abnormal Cardiac Development

From the above data, we propose a model in which a breakdown in the placental tissue barrier, because of high local inflammation, promotes cardiac-selective influx of proinflammatory maternal leukocytes, which affects normal cardiac development. To address this hypothesis, we targeted TNF-α–mediated placental inflammation by injecting NDPI pregnant mice with a neutralizing anti–TNF-α antibody (referred hereafter as NDPI+aTNF-α; see Figure S12A for scheme). Blocking TNF-α reduced placental TNF-α levels, rescued the poor placental tissue architecture, enabled deeper trophoblast invasion, and restored placenta collagens at both the gene and protein level (Figure S12B–S12E).

**Figure 7.**
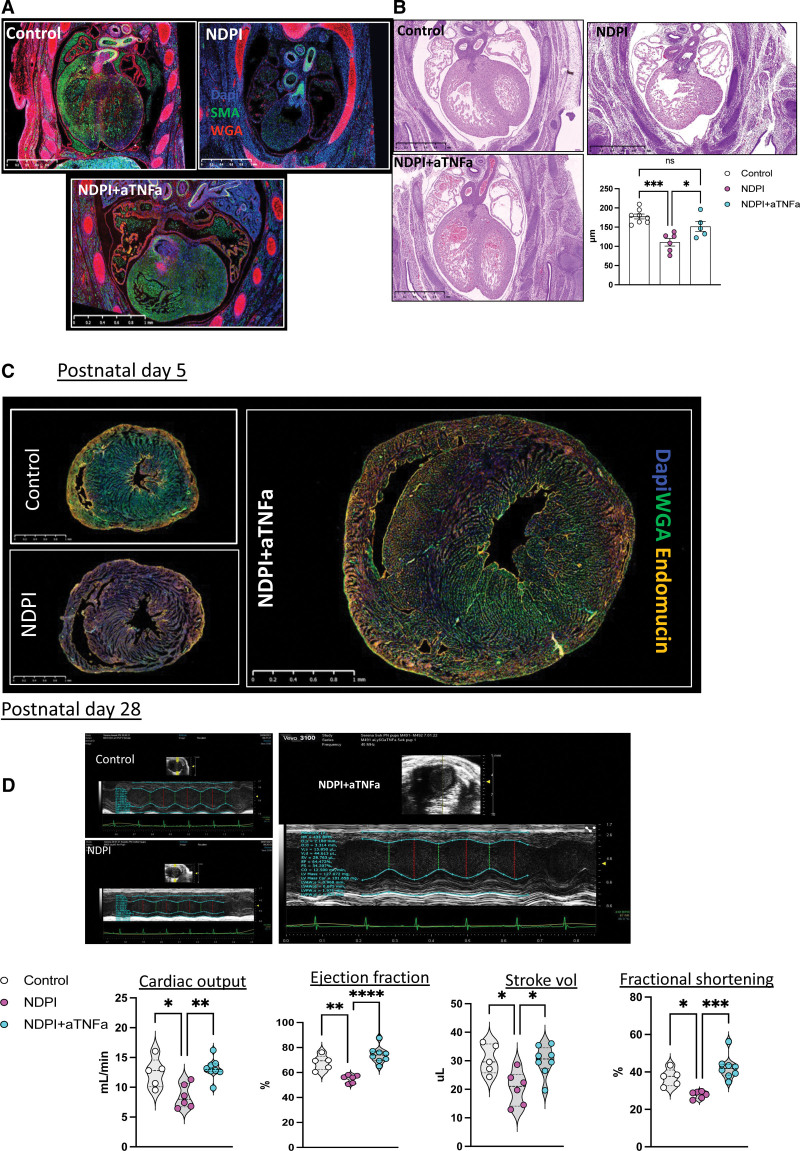
**Quelling placental inflammation prevents abnormal cardiac development.** Neutrophils were depleted at days 4.5 and 7.5 of pregnancy with αLy6G. At day 8.5 of pregnancy, tumor necrosis factor-α (TNF-α) was neutralized by injecting aTNF-α immunoglobulin G (IgG) intravenously. Mice were euthanized at embryonic day (E) 14.5 of pregnancy, and placentas and embryos were harvested to assess the structure and immune composition. Control is shown in white, neutrophil-driven placental inflammation (NDPI) in pink, and neutrophil-depleted and TNF-α neutralized (aTNF-αNDPI) in blue. **A**, Immunofluorescent images of cross section of E14.5 embryo heart. Sections were stained with DAPI (blue), wheat germ agglutinin (WGA; red), and smooth muscle actin (SMA; green). Graph showing ventricular wall thickness quantification. **B**, Hematoxylin and eosin sections of E14.5 embryonic hearts from aTNF-αNDPI pregnancies compared with control Graph represents left ventricular wall thickness. **C**, Immunofluorescent images showing the expression of endomucin (orange) and WGA (green) in cross sections of hearts from postnatal day (P) 5. Scale bar, 1 mm. Magnified images showing endomucin staining of the ventricle. Graph showing the proportion of total CD31^+^ cells in P5. **D**, Echocardiography plot with graphs (**bottom**) showing quantification of heart parameters determined by echocardiography. Each symbol represents an individual mouse, and statistical significance was tested by 1-way ANOVA with Bonferroni post hoc correction. In all cases, data are mean±SEM. ns Indicates not significant. *P≤0.05. **P≤0.01. ***P≤0.001. ****P≤0.0001.

Fewer activated placental neutrophils were counted, expressing lower levels of TNF-α, CXCR2, CD114, and matrix metalloproteinase-9 from NDPI+aTNF-α pregnancies compared with NDPI, coupled with reduced numbers of inflammatory monocytes (Figure S12F and S12G). After maternal aTNF-α treatment, fewer maternal cells were recruited into the embryonic hearts (Figure S12H) and the proportion of CX_3_CR1^+^CCR2^−^ resident fetal macrophages increased compared with both control and NDPI pregnancies (Figure S12I). This was coupled with a downregulation in both IL-1β and IL-6 gene expression (Figure S12J). These cellular changes yielded a well-defined cardiac structure, resembling the structure of control embryonic hearts (Figure 7A), including LV wall thickness (Figure 7B) and a restoration in the proliferation of both CD31^+^ endothelial cells and cardiomyocytes (Figure S12K).

Postnatally (P5), we observed an ≈2-fold increase in the proportion of CX_3_CR1^+^CCR2^−^ macrophages relative to CX_3_CR1^+^CCR2^+^ cells in NDPI+aTNF-α compared with P5 hearts from NDPI offspring (Figure S12L). Heart:body weight ratios from offspring of NDPI+aTNF-α were akin to control offspring (Figure S12M). We also observed a loss in the hypertrabeculation displayed by NDPI pregnancies and a more compact endocardium within the cardiac tissue, accompanied by a significant increase in CD31^+^ endothelial cells in NDPI+aTNF-α offspring (Figure 7C).

Last, we observed a restoration in cardiac function of adult (P28) hearts of NDPI+aTNF-α offspring compared with their NDPI counterparts (Figure 7D), which coincided with fewer F4/80^+^Ly6C^+^ macrophages within the cardiac tissue. Cardiac macrophages displayed a more quiescent, anti-inflammatory phenotype, expressing significantly lower levels of MHC II and CCR2 compared with their NDPI counterparts (Figure S12N).

Taken together, these data suggest that tempering NDPI with TNF-α neutralization is sufficient to rescue cardiac development and function in offspring postnatally and into adulthood.

## Discussion

We describe how placental inflammation, driven by activated neutrophils, promotes aberrant embryonic heart development that affects cardiac structure and downstream cardiac function in postnatal life. Specifically, locally produced neutrophil TNF-α promotes the establishment of an inflammatory placental environment with breakdown of tight tissue barriers; this in turn allows the transfer of placental inflammatory maternal monocytes to the embryonic heart, impeding its normal development, leading to persistent heart dysfunction.

After neutrophil depletion, an activated circulating neutrophil phenotype emerges, with high levels of CXCR2 and granulocyte colony-stimulating factor receptor CD114. Consequently, activated neutrophils are present within the placental tissue, coupled with high expression of TNF-α and matrix metalloproteinase-9. This neutrophil-driven inflammatory environment is responsible for matrix degradation of the placentas, with repressed collagen gene and protein expression.

The complex structure of the placenta barrier ensures that maternal leukocytes do not enter the fetal compartment, and this guarantees the tolerogenic state of the placenta.^39^ However, maternal cells can cross the barrier and, through the fetal circulation (called maternal microchimeric cells), enter fetal organs, including liver, lung, pancreas, and heart.^40^ A recent study identified maternal microchimeric cells in cardiac tissue of infants who died of neonatal lupus with heart block,^41^ suggesting that cells of maternal origin may reach the heart to affect cardiac responses in offspring. Using the CD45.1 (maternal) and CD45.2 (paternal) system, as well as GFP leukocyte transfer experiments, we identified a significant increase in the number of maternal (CD45.1^+^CD45.2^−^) inflammatory macrophages in embryonic hearts from NDPI pregnancies. The phenotype of the maternal cells is akin to the maternal inflammatory monocytes detected in the placenta. Analyses of chemokines in embryonic hearts and livers identified an embryonic heart-specific (but not liver-specific) chemokine ligand profile resulting from NDPI pregnancies (namely CCL3, CCL4), which matched reciprocal chemokine receptors on maternal cells in both placenta and embryonic heart. This raises a question: What may be responsible for altering the environment of the developing heart? The timing of both neutrophil depletions in our model precedes maternal inflammatory immune cell infiltration because the first depletion is carried out at E4.5, which is before both placenta and heart development, and the second depletion at E7.5, at the beginning of placental and heart development.^42^ This suggests that manipulation of the maternal quiescent neutrophil response in early gestation may prime the placenta and, subsequently, the embryonic heart to a proinflammatory environment.

Placental development occurs in parallel to embryonic cardiac development, and studies have highlighted the reciprocal influence these organs exert on each other’s development.^5,43^ Indeed, evidence suggests that women who have preeclampsia during their pregnancy have a significant increased risk of their fetuses developing a CHD,^8^ attributed to the poor placental development that occurs in preeclamptic pregnancies, including poor trophoblast invasion and aberrant oxygen and nutrient transfer from the mother.^7^ There may also be a genetic link between placental development in early-onset preeclampsia and the development of fetal CHDs whereby epigenetic programming of placental and fetal tissues leads to an increase of cardiac anomalies in offspring of preeclamptic pregnancies.^44^

Embryonic hearts from NDPI pregnancies display hypertrabeculation and thinner LV, reminiscent of the congenital heart condition LVNC.^22^ Elegant studies have demonstrated recently that resident cardiac fetal macrophages populate distinct regions of the developing heart: CX3CR1^+^CCR2^−^ macrophages predominantly populate the myocardial wall, and CX3CR1^+^CCR2^+^ macrophages are found in the trabecular projections.^11^ However, the exact function during embryonic heart development of this latter subtype is currently unclear. It is noteworthy that the presence of CCR2^+^ macrophages within the embryonic and neonatal heart is short-lived, whereas their CCR2^−^ counterparts are self-renewing.^12,33^ The current data add another layer of complexity to this dichotomy whereby the presence of inflammatory maternal cells within the developing heart can promote the induction of these CCR2^+^ macrophages. In adult cardiac tissue, the roles of CCR2^−^ and CCR2^+^ macrophages are more apparent: Resident CCR2^−^ macrophages of fetal origin have a reparative function, whereas CCR2^+^ monocyte-derived macrophages promote myocardial injury and inflammation.^38^ Our findings indicate that, within the embryonic heart, maternal inflammatory cells can skew toward a fetal CCR2^+^ inflammatory macrophage phenotype, which coincides with a distinct LVNC-like tissue architecture. These CCR2^+^ fetal macrophages may function to promote embryonic cardiac injury, or they may create an inflammatory environment that subsequently affects heart development. This may suggest why these cells are short-lived under normal heart developmental conditions. Postnatally, although there was no presence of inflammatory maternal cells in offspring hearts, an inflammatory cardiac macrophage phenotype persisted in P5 and P28 hearts from offspring of NDPI pregnancies, coupled with poor cardiac tissue architecture and function. Together, these data indicate that the influence of inflammatory maternal cells in embryonic heart development is imprinted in postnatal life and add support to the concept that the innate immune competency of the placenta is key to the leukocyte composition of the developing heart.

### Conclusions

We present a mechanistic paradigm whereby neutrophil-driven inflammation in pregnancy can preclude normal embryonic heart development as a direct consequence of poor placental development. It is important to note that this study also opens translational avenues for early diagnoses and potential treatment of CHDs in utero. The former assertion is evidenced by the finding that NDPI pregnancies are associated with an activated neutrophil phenotype within the maternal circulation. Women with preeclampsia present an activated neutrophil phenotype^14,16^ and may produce a higher incidence of CHDs in offspring.^45^ Thus, early phenotyping of neutrophils from pregnant women could provide an early diagnostic test to identify CHDs in fetuses. Last, early identification of placental inflammation could be mitigated through a therapeutic intervention such as anti–TNF-α used here. Thus, anti-inflammatory therapy can restore normal embryonic and postnatal heart development and function, offering a potential alternative to current invasive in utero interventions to treat CHDs.

## Article Information

### Acknowledgments

The authors thank Mason Arnold and James Gillett at the Biological Services Unit, Queen Mary, London, for their invaluable assistance with the animals during and after the coronavirus disease 2019 (COVID-19) lockdown. They also thank Dr Kevin Blighe of Clinical Bioinformatics Research Ltd (London, UK) for analyses of the single-cell sequencing data. E.J.W. performed experiments, analyzed data, and helped write the manuscript. S.B. performed experiments and analyzed data. S.F. performed experiments and analyzed data. K.M.M. and R.T.M. analyzed RNA sequencing data and provided figures. G.K. performed experiments. F.P. processed and produced HREM images. L.K.V. and A.P.P. provided human placenta tissue; G.G. provided CCR2^−/−^ mice and provided discussion of the manuscript. N.P.D. performed experiments, analyzed data, and helped write the manuscript. M.P. and F.M.M.-B. provided critical discussion of experiments and the manuscript. S.N devised the concept; planned, performed, and supervised experiments; analyzed data; wrote the manuscript; and composed figures. Further information and requests for resources and reagents should be directed to and will be fulfilled by the corresponding author. Links to data set raw data for bulk RNA sequencing and single-cell sequencing can be found in the Methods section.

### Sources of Funding

This work was funded by a British Heart Foundation intermediate basic science research fellowship (SN; FS/17/1/32528). This work is aligned with the British Heart Foundation Accelerator Award (AA/18/5/34222) to Queen Mary, which focuses on cardiac inflammation; Wellcome Trust Investigator Award Wellcome Trust investigator award (GJG;217093/Z/19/Z; GJG); and MRC programme grant (GJG; MRV0109721). The flow cytometry core facility was funded by CRUK (core award C16420/A18066).

### Disclosures

None.

### Supplemental Material

Expanded Methods

Figures S1–S13

References 46–50

## Supplementary Material


