# Placental inflammation leads to abnormal embryonic heart development

**DOI:** 10.1161/CIRCULATIONAHA.122.061934

**Published:** 2022-12-09

**Authors:** Eleanor J Ward, Serena Bert, Silvia Fanti, Kerri M Malone, Robert T Maughan, Christina Gkantsinikoudi, Fabrice Prin, Lia Karina Volpato, Anna Paula Piovezan, Gerard J Graham, Neil P Dufton, Mauro Perretti, Federica M Marelli-Berg, Suchita Nadkarni

**Affiliations:** 1 William Harvey Research Institute, Queen Mary University of London, Charterhouse Square, London, EC1M 6BQ, UK; 2 European Bioinformatics Institute (EMBL-EBI), Wellcome Genome Campus, Hinxton, Cambridge, CB10 1SD, UK; 3 National Heart and Lung Institute, Imperial College London, London SW3 6LY, UK; 4 Crick Advanced Light Microscopy Facility, The Francis Crick Institute, London NW1 1AT, UK; 5 Postgraduate Program in Health Science, University of Southern Catarina (UNISUL), Campus Pedra Branca, Palhoça, SC, Brazil; 6 Institute of Infection, Immunity and Inflammation, University of Glasgow, Glasgow, G12 8TA

**Keywords:** Placenta, heart, inflammation, neutrophils, macrophages, maternal, fetal, congenital heart disease

## Abstract

**Background:**

Placental and embryonic heart development occurs in parallel, and these organs have been proposed to exert reciprocal regulation during gestation. Poor placentation has been associated with congenital heart disease (CHD), an important cause of infant mortality. However, the mechanisms by which altered placental development can lead to CHD remain unresolved.

**Methods:**

In the study we use an *in vivo* neutrophil-driven placental inflammation model via antibody depletion of maternal circulating neutrophils at key stages during time-mated murine pregnancy – embryonic day (E)4.5 and E7.5. Pregnant mice were culled at E14.5 to assess placental and embryonic heart development. A combination of flow cytometry, histology and bulk RNA-sequencing was used to assess placental immune cell composition and tissue architecture. We also used flow cytometry and single-cell sequencing to assess embryonic cardiac immune cells at E14.5 and histology and gene analyses to investigate embryonic heart structure and development. In some cases, offspring were culled at postnatal day (P)5 and P28 to assess any postnatal cardiac changes in immune cells, structure and cardiac function, as measured by echocardiography.

**Results:**

In the current study we show that neutrophil-driven placental inflammation leads to inadequate placental development and loss of barrier function. Consequently, placental inflammatory monocytes of maternal origin become capable of migration to the embryonic heart and alter the normal composition of resident cardiac macrophages and cardiac tissue structure. This cardiac impairment continues into postnatal life, hindering normal tissue architecture and function. Finally, we show that tempering placental inflammation can prevent this fetal cardiac defect and is sufficient to promote normal cardiac function in postnatal life.

**Conclusions:**

Taken together, these observations provide a mechanistic paradigm whereby neutrophil-driven inflammation in pregnancy can preclude normal embryonic heart development as a direct consequence of poor placental development, which have major implications on cardiac function into adult life.

## Introduction

Congenital heart diseases (CHDs) are an important cause of stillbirths, with ~11% stillbirths attributed to a type of CHD^1^, and associated with ~35% of infant deaths^2^. The heart is one of the first organs to develop in the human embryo, the first stages beginning by the end of the 2^nd^ week of pregnancy^3^. In a series of coordinated developmental stages, the heart begins as a primitive heart tube developing into the four-chamber organ by the 8^th^ week of pregnancy, with a detectable heart rate^4^. By the 2^nd^ trimester, the heart can start pumping blood around the fetus. The placenta is a specialized organ that acts as a tight barrier to regulate the transfer of oxygen and nutrients to the developing fetus, whilst preventing passage of harmful pathogens and cells. Placental and embryonic heart development occur in parallel, suggesting that the two organs influence each other’s development^5^. Clinical studies suggest a strong association between placental dysfunction and CHDs^6^, with poor trophoblast invasion, and aberrant oxygen and nutrient transfer from the mother, leading to poor fetal cardiac development^7^. This is compounded by evidence suggesting women who have preeclampsia during their pregnancy have a significant increased risk of their fetuses developing a CHD^8^.

The role of tissue-resident macrophages in promoting normal organogenesis is well established ^9^. At around E8.5, yolk sac-derived erythro-myeloid progenitors (EMP) migrate to the developing embryo in a chemokine-dependent manner. These EMPs develop into pre-macrophages expressing CX_3_CR1, Kit and CSFR1 and seed various organs including brain, liver, and heart, where they persist into adulthood through a process of self-renewal ^10^. In the heart, CX_3_CR1^+^CCR2^**-**^ macrophages migrate from the yolk sac to promote angiogenesis, regulate coronary vascular development^11^, and exert reparative functions in adult cardiac tissue ^12^. This contrasts with blood monocyte-derived CCR2^+^ macrophages that promote inflammation in the adult heart ^12, 13^.

We have previously demonstrated that our model of maternal neutrophil depletion during murine pregnancy indues a preeclampsia-like phenotype, typified by abnormal placental development including shallow invasion of trophoblasts into the decidua and subsequent remodelling of the spiral arteries^14^. In the current study, we revisit this model and demonstrate that maternal neutrophil depletion promotes placental inflammation and a breakdown in the tight placental tissue barrier, both key features of preeclampsia placental phenotype^15, 16^. We go onto show this neutrophil-driven placental inflammation (NDPI) model allows migration of inflammatory maternal monocytes to the embryonic heart, which, in turn, promotes abnormal fetal cardiac development with inadequate cardiac function in postnatal and adult life.

## Methods

The data that support the findings of this study are available from the corresponding author upon reasonable request

Clinical samples were approved by the Institutional Review board University of Southern Santa Catarina (UNISUL) under 34681920.8.0000.5369. Animal studies were conducted with strict adherence to the Home Office guidelines (PPL P71E91C8E). A detailed description of the methods are available in the Supplemental Material.

Please see Figure S13 for flow cytometry gating strategy and antibody and primer information. All raw RNA-sequencing data and single cell sequencing data have been deposited on Figshare (https://figshare.com/s/8b13463311cf442e9d15), (https://figshare.com/s/98321569e7f6a15aff65 and https://figshare.com/s/83bf3f8ba06d4884d827).

### Statistical analyses

Statistical analyses were carried out using Prism software (GraphPad, version 9). In all cases data were tested for normality using the Kolmogorov-Smirnov test. For data comparing control Vs NDPI and had equal variances, two-tailed Student’s t-test was used. For unequal variance, Welch’s t-test was used, and Mann-Whitney test for nonparametric comparison. For data comparing 3 or more groups, where data had equal variance, we carried out one-way ANOVA with post hoc Bonferroni test Comparison of data with unequal variance was tested using the Brown-Forsythe ANOVA, followed by post hoc Dunnett’s test. Two-way ANOVA was used to determine statistical significance between groups and two or more cell populations. In all cases *P<0.05* was considered significant.

## Results

### A Neutrophil-driven model of placental inflammation

**Figure 1:**
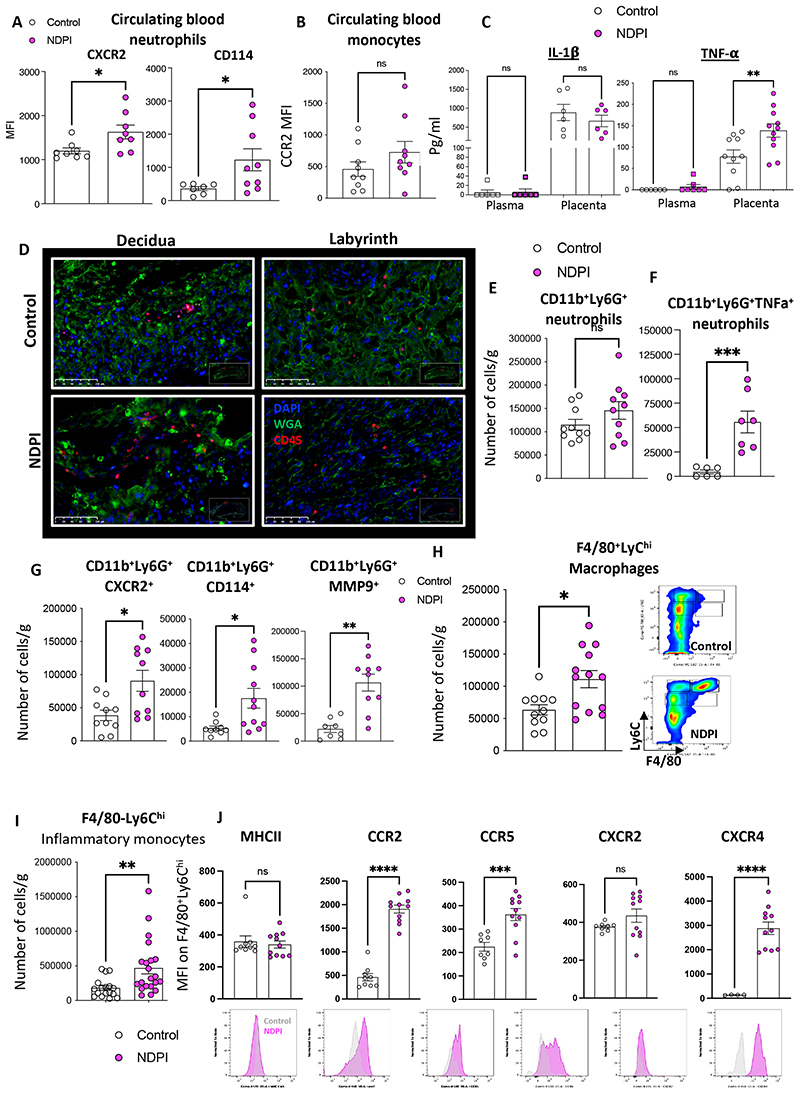
A Neutrophil-driven model of placental inflammation Neutrophils were depleted at day 4.5 and 7.5 of pregnancy using αLy6G. Mice were sacrificed at E14.5 of pregnancy and placentas harvested to assess the structure and immune composition. Isotype control treated (referred to hereafter as control; white) and neutrophil depleted (referred to hereafter as NDPI; pink). (A) Expression of activation markers by maternal blood neutrophils (B) CCR2 expression on maternal blood monocytes (C) ELISA showing the concentration of IL-1β and TNF-α in plasma and placenta digest supernatants. (D) Immunofluorescent staining of placentas for CD45 (red) WGA (green) and cell nuclei with DAPI (blue). Expression is shown in the decidual layer (left) and labyrinth layer (right). (E-G) Neutrophil subpopulations from placentas analyzed by flow cytometry expressed as absolute cell number per gram of tissue. (E) CD11b^+^Ly6G^+^ neutrophils, (F) CD11b^+^Ly6G^+^ TNFα^+^ neutrophils, (G) neutrophils expressing CD11b^+^Ly6G^+^ CXCR2, CD11b^+^Ly6G^+^ CD114+ and CD11b^+^Ly6G^+^ MMP9^+^. (H) Macrophages from placentas analyzed by flow cytometry expressed as absolute cell number per gram of tissue. (I) Inflammatory monocytes from placentas analyzed by flow cytometry expressed as absolute cell number per gram of tissue. (J) F4/80^-^Ly6C^hi^ populations from placentas were analyzed for the expression of MHCII, CCR2, CXCR2, CCR5 and CCR6, expressed as median fluorescent intensity. Representative histograms are shown below the quantification (control= grey, NDPI =pink). Each symbol represents an individual mouse from different pregnancies and statistical significance was tested by unpaired Student’s t-test or Welch’s t-test (G – CXCR2 and CD114 and H). ns = not significant, *p≤0.05, ** p≤0.01, ***p≤0.001 **** p≤0.0001. In all cases, data are mean ± SEM. MFI, median fluorescence intensity

Following antibody depletion (anti-Ly6G, clone IA8), neutrophils return to the circulation within 72hrs ^22^ and these cells now present an activated, pro-inflammatory phenotype, characterized by high CXCR2 and CD114 (G-CSFR) expression. No differences were observed in circulating proinflammatory CCR2^+^ monocytes (Figure 1 A). We next investigated the placental environment in more detail. Maternal neutrophil depletion resulted in smaller placentas and shallow trophoblast invasion (Figure S1B and S1C), coupled with an exaggerated TNF-α placental concentration, but not the circulation (Figure 1C). There was no overall difference in total CD45^+^ leukocyte numbers (Figure 1D and Figure S1D), nor in numbers of CD3^+^ T cells and NK cells (Figure S1E). Although placentas from neutrophil-depleted mothers displayed no overall difference in the number of neutrophils compared to their isotype control (referred to hereafter as control) counterparts (Figure 1E), placental neutrophils displayed an activated phenotype with high expression of TNF-α,CXCR2, CD114 (G-CSFR), and MMP9 (Figures 1F and G). Therefore, neutrophil depletion in pregnant mice induces ***neutrophil-driven placental inflammation*** (referred to hereafter as NDPI).

NDPI was also defined by presence of activated F4/80^+^Ly6C^hi^ macrophages (Figure 1H), with no difference in the number of these macrophages making TNF-α between both groups (Figure S1F), suggesting neutrophils are the likely source of increased TNF-α in NDPI. Intra-placental monocytes displayed an inflammatory phenotype compared to control (Figure 1I). These F4/80^-^Ly6C^hi^CCR2^+^ monocytes expressed higher levels of CCR2, CCR5 and CXCR4, but not MHC II or CXCR2 (Figure 1J) compared control. Lack of difference in total leukocyte numbers between placentas from control and NDPI pregnancies (Figure S1D), coupled with no overall change in the inflammatory status of circulating maternal monocytes, indicated that the phenotype of placental macrophages and monocytes is not due to an influx from the maternal circulation, but rather to *in-situ* activation. Thus, we hypothesized that TNF-α producing neutrophils within the placental tissues regulate the phenotype of placental monocytes and macrophages. To challenge this hypothesis, we isolated placental neutrophils from control and NDPI pregnancies and co-cultured them with naïve splenic monocytes from non-pregnant aged-matched mice. Co-cultures induced higher proportions of inflammatory F4/80^+^Ly6C^hi^ macrophages expressing MHC II and CCR2 following monocyte exposure to NDPI neutrophils, mirroring the inflammatory phenotype observed in NDPI *in vivo* (Figure S1G and S1H).

### Neutrophil-driven Inflammation promotes a breakdown in placental tissue barrier

**Figure 2:**
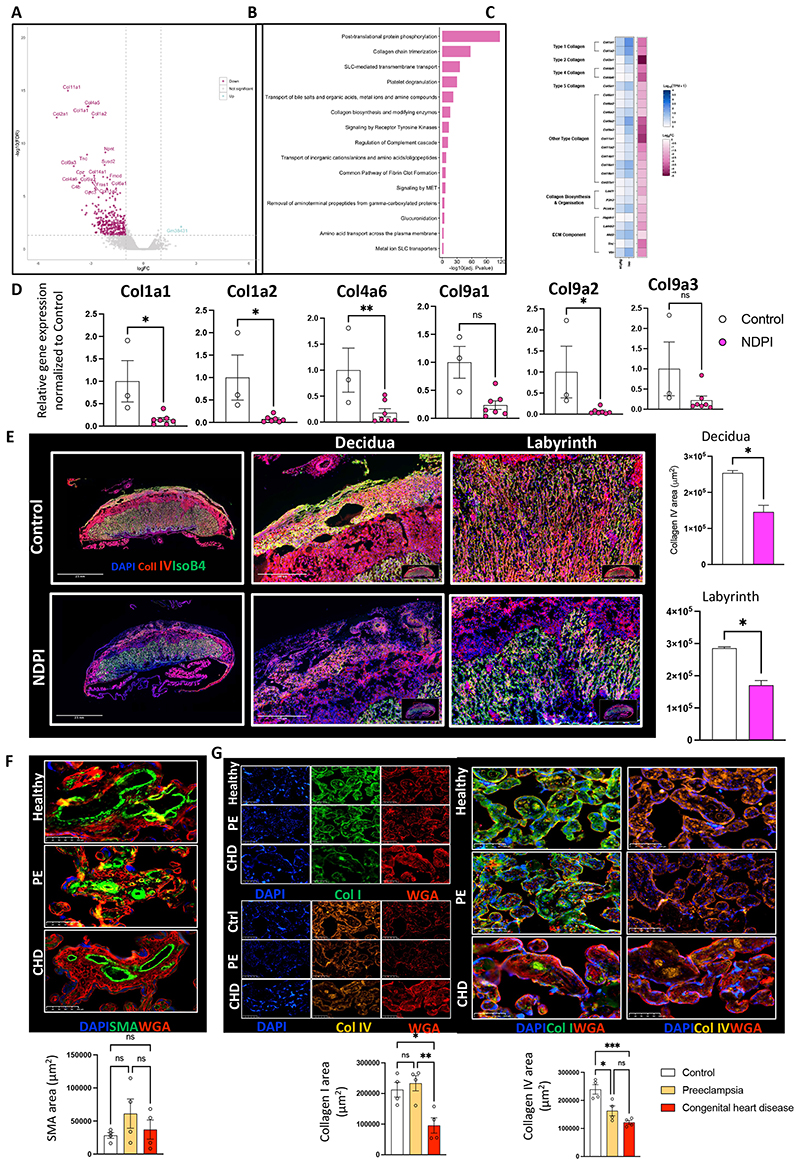
Neutrophil-driven Inflammation promotes a breakdown in the placental tissue barrier (A-E) Neutrophils were depleted at day 4.5 and 7.5 of pregnancy using αLy6G. Mice were sacrificed at E14.5 of pregnancy and placentas harvested. Control (white) and NDPI (pink). (A-C) RNA sequencing from placentas. (A) Volcano plot showing differentially expressed genes in control versus NDPI placentas (B) Top 15 modulated pathways in placentas of NDPI mice when compared to control (C) Heat map showing gene expression of extracellular matrix components of NDPI or control placentas (D) RT-PCR showing the gene expression of Col1a1, col1a2, col4a6, col5a1, col9a1, col9a2 and col9a3 (E) Immunofluorescent images of control or NDPI murine placentas, stained with DAPI (blue) collagen IV (red) isolectin B4 (red). Magnified images of the decidua and labyrinth of the placenta and graphs showing the quantification of Col IV staining. (F-G) Immunofluorescent staining of term placentas from women with healthy, pre-eclamptic pregnancies or pregnancies carrying babies with congenital heart disease for (F) SMA (green) (G) Col I (green) (top panel) Col IV (orange) bottom panel. In all panels WGA staining in red and cell nuclei are stained with Dapi (blue). Quantification of area stained with SMA, Col I and Col IV shown in panel below images Each symbol represents an individual sample from different pregnancies and statistical significance was tested by unpaired Student’s t-test or Welch’s t-test (E) or one-way ANOVA (F and G). ns = not significant, *p≤0.05, ** p≤0.01, **p≤0.001. In all cases, data are mean ± SEM.

To further investigate the features of NDPI we undertook bulk RNA sequencing of E14.5 placental tissues from control and NDPI pregnancies. In NDPI we observed downregulation of 329 genes, out of 357 genes in total to be significantly changed (false detection rate, FDR, corrected P<0.05) (Figure 2A). Pathway analyses identified post-translational protein phosphorylation, collagen trimerization as the top pathways (Figure 2B).

We focused our attention on collagen genes, since these extracellular matrix (ECM) components are important for placental tissue integrity^23^. Further analyses revealed that of the 44 collagen genes within the mouse genome, 17 were significantly downregulated in NDPI (Figure S2A): including *Col1a1 and Col1a2, Col2a1, Col4a6, Col9a2* and *Col9a3, Col11a1, Col11a2* and *Col11a3* (Figure 2C and 2D). Immunofluorescent staining for two collagens (type I and IV) demonstrated lower expression of collagen I in the decidua of NDPI placentas, but no difference in the labyrinth, compared to control (Figure S2B). For collagen IV, significant reductions were quantified in both decidua and labyrinth of NDPI placentas (Figure 2E). Collagen IV is also required for the invasive properties of trophoblasts^24^, suggesting reduced collagen IV expression may explain the shallow trophoblast invasion displayed in NDPI settings.

We next sought to establish whether these collagen matrix features are present in human tissues in two types of pregnancy complications that affect placental development (Table 1). We assessed term placental tissue from preeclamptic pregnancies (PE), chosen because of an activated neutrophil environment in these patients^14, 16^; and from pregnancies with fetuses that have CHD in the absence of maternal PE. Both were compared normal, uncomplicated pregnancies. There was no significant changes in smooth muscle actin expression surrounding the maternal spiral arteries in all 3 placentas (Figure 2F). Collagen I from CHD placentas was significantly attenuated compared to healthy and PE placentas (Figure 2G), whereas collagen IV was significantly downregulated in both PE and CHD placentas compared to healthy (Figure 2G). Together, these data indicate NDPI has a negative functional impact on the placental support structure.

### Placental inflammation leads to poor embryonic cardiac development

**Figure 3:**
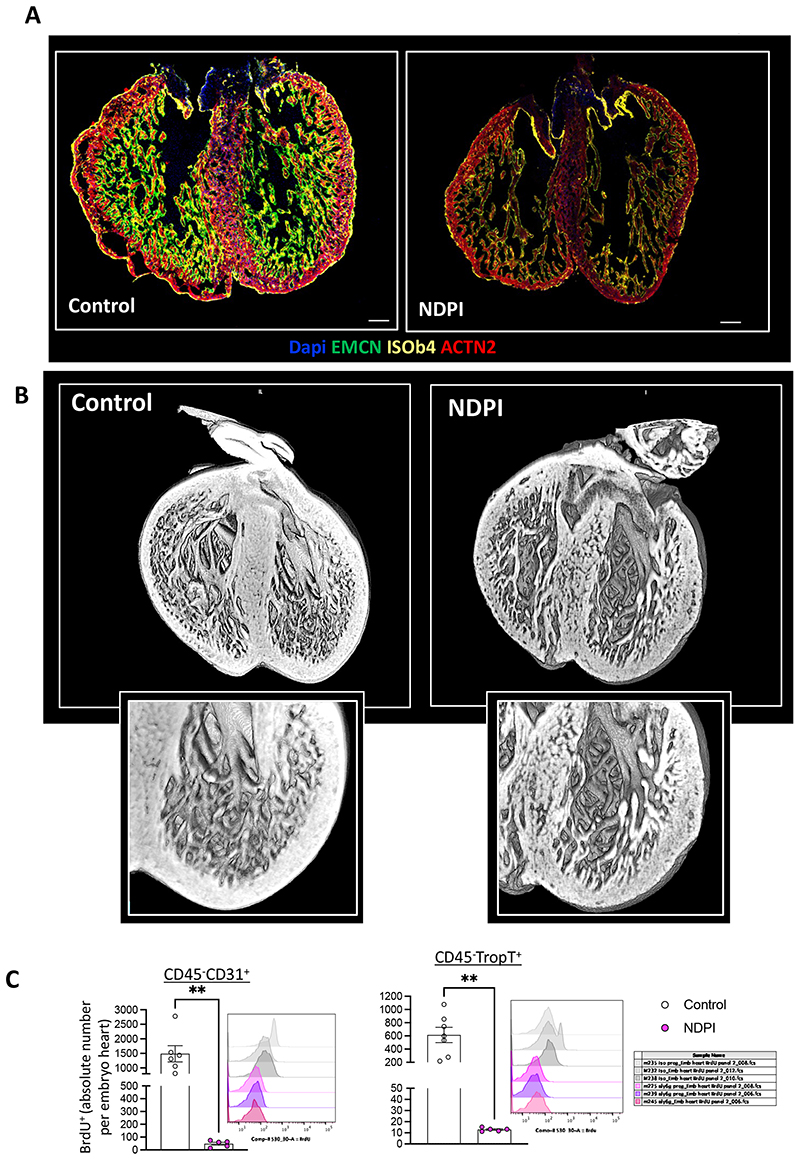
Placental inflammation leads to poor embryonic cardiac development Neutrophils are depleted at day 4.5 and 7.5 of pregnancy using αLy6G. Mice were sacrificed at E14.5 of pregnancy and embryos harvested. Control (white) NDPI (pink). (A) Immunofluorescent images of E14.5 embryo heart of cross section. Sections were stained with DAPI (blue) endomucin (EMCN; green), isolectin B4 (ISOb4; yellow) and Actinin-2 (ACTN2; red) (B) High-resolution episcopic microscopy images of E14.5 hearts (C) Quantification of the *in vivo* uptake of BrdU into CD45^-^CD31^+^ endothelial cells and CD45^-^Troponin-T^+^ cardiomyocytes in embryo hearts. Each symbol represents an individual mouse and statistical significance was tested by unpaired Student’s t-test. ns = not significant, *p≤0.05, ** p≤0.01, ***p≤0.001 **** p≤0.0001. In all cases, data are mean ± SEM.

We next investigated the association between poor placentation and cardiac development in utero. Initial observations revealed abnormal heart development in the gross structure of embryonic hearts at E14.5 from NDPI pregnancies, with significantly thinner compact myocardium within LV the compared to control, coupled with attenuated endomucin (endocardial cell marker) staining within the total heart area, indicating poor vascularization (Figure 3A and Figure S3A-C). However, this defect appeared to be restricted to the endocardium, as we observed no difference in expression on epicardial-specific WT-1 (Figure S3D), nor in gene expression of epicardial-specific *Tcf21* and *Sema3d*. We observed NDPI embryonic hearts have impaired TGFβ-activity displaying reduced phosphorylation of SMAD3 (Figure S4A) and diminished expression of downstream TGF-SMAD3 target, transgelin (TAGLN), at E14.5 (Figure S4B). SMAD3 is required for the activation of TAGLN ^25^, suggesting a potential defect in SMC formation in NDPI E14.5 hearts. Moreover, *Tagln-Cre:Tgfbr2* knockout mice display a less compact and thinner LV wall^26^.

Between E9.0 and E9.5, primitive cardiomyocytes within the ventricular wall form finger-like projections called trabeculae that are lined by endocardial cells. As cardiac development progresses, ventricles undergo a switch from a mostly trabecular to a compacted state where cardiomyocytes compact and increase ventricular wall thickness, and is essential for normal heart function. Dysregulation of this switch can cause hypertrabeculation, leading to a congenital cardiomyopathy named left ventricular non-compaction (LVNC)^27^. 3-D imaging using high resolution episcopic microscopy revealed increased trabeculation of the ventricular walls of hearts from NDPI embryos, compared to controls, both in terms of number and length (Figure 3B Figure S5A), indicative of a LVNC-like phenotype. Recent studies have demonstrated dysregulation of coronary endothelial cells promotes LVNC^28^. With this in mind, we interrogated the status of the proliferation of endothelial cells (ECs) within the embryonic heart. Flow cytometric analyses revealed reduced numbers of proliferating CD31^+^ EC in NDPI embryonic hearts compared to controls as assessed by *in vivo* BrdU incorporation (Figure 3C, left panel), as well as a significant reduction in phospho histone H3 staining as indicated by immunofluorescence (Figure S5B). We also observed attenuated expression of key regulators of angiogenesis including ICAM-1, VCAM-1, endoglin, and thrombospondin (Figure S5C). These data indicate NDPI impedes normal embryonic heart development by hindering embryonic heart vascularization. This attenuation in endothelial cell proliferation was accompanied by a significant downregulation in the number of proliferating troponin-T^+^ cardiomyocyes (Figure 3C, right panel), adding further support that embryonic hearts from NDPI pregnancies develop abnormally.

### CCR2 driven accumulation of maternal proinflammatory leukocytes in embryonic hearts of NDPI pregnancies

**Figure 4:**
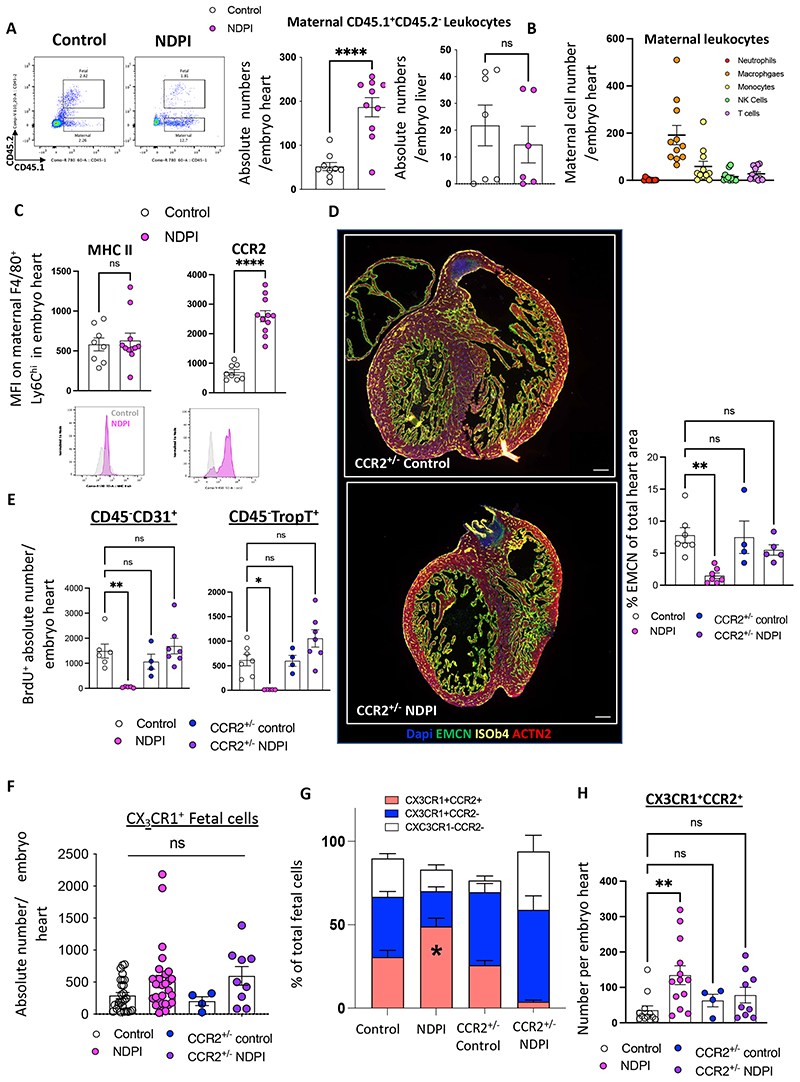
CCR2 driven accumulation of maternalproinflammatory leukocytes in embryonic hearts of NDPI pregnancies Neutrophils were depleted at day 4.5 and 7.5 of pregnancy using αLy6G. Mice were sacrificed at E14.5 of pregnancy and hearts dissected from harvested embryos. Control (white) and neutrophil depleted (NDPI) (pink). (A) Flow cytometry plots of leukocytes in E14.5 fetal hearts from and NDPI pregnancies; CD45.1^+^ CD45.2^-^ cells are of maternal origin and CD45.1^+^CD45.2^+^ cells are of fetal origin. Graphs show quantification of number of maternal cells per embryo heart or embryo liver. (B) Flow cytometry quantification of different maternal leukocyte subsets in embryo hearts. (C) Median fluorescent intensity of MHCII and CCR2 on maternal F4/80+Ly6Chi cells found in embryo hearts. Lower panel, representative histograms. (D) Immunofluorescent images of E14.5 embryo heart of cross section from CCR2^+/-^ control and CCR2^+/-^ NDPI pregnancies. Sections were stained with DAPI (blue) endomucin (EMCN; green), isolectin B4 (ISOb4; yellow) and Actinin-2 (ACTN2; red). Graph indicated quantification of endomucin staining of embryonic hearts expressed as %of total heart area. (E) Quantification of the *in vivo* uptake of BrdU into CD45^-^CD31^+^ endothelial cells and CD45^-^Troponin-T^+^ cardiomyocytes in embryo hearts from CCR2^+/-^ control and CCR2^+/-^ NDPI pregnancies, compared to control. (F) Quantification of absolute numbers of CX3CR1 fetal cells in E14.5 embryonic hearts from control, NDPI, CCR2^+/-^ control and CCR2^+/-^ NDPI pregnancies (G) Proportion of CX_3_CR1^+^CCR2^+^ and CX_3_CR1^+^CCR2^-^ fetal cells in E14.5 embryonic hearts from control, NDPI, CCR2^+/-^ control and CCR2^+/-^ NDPI pregnancies (H) Absolute number of CX3CR1^+^CCR2^+^ fetal cells in E14.5 embryonic hearts from control, NDPI, CCR2^+/-^ control and CCR2^+/-^ NDPI pregnancies Each symbol represents an individual mouse and statistical significance was tested by unpaired Student’s t-test. ns = not significant, *p≤0.05, ** p≤0.01, ***p≤0.001 **** p≤0.0001 (A-C). One -way ANOVA, with Dunnett’s multiple comparison test, compared to Control (D-F &H), Brown-Forsythe ANOVA test with Dunnett’s multiple comparison test (E, Trop-T);or two-ANOVA, comparing means of CCR2+ or CCR2-% between mouse groups with Tukey’s multiple comparison test (G). In all cases, data are mean ± SEM. MFI, median fluorescence intensity

To test whether immune cells from inflamed placentas have the capacity to directly shape embryonic heart development, we utilized the CD45.1/CD45.2 system, which allows discrimination of immune cells of maternal or fetal origin (see Methods). Maternal cells were identified as CD45.1^+^CD45.2^-^ (referred to hereafter as maternal), whereas fetal cells were identified as CD45.1^+^CD45.2^+^ (referred to hereafter as fetal). There was a 4-fold increase in the absolute number of maternal cells in the NDPI embryonic hearts compared to control, with no significant differences observed in maternal cells in the fetal liver (Figure 4A). The detection of maternal leukocytes in NDPI embryonic hearts was confirmed via adoptive transfer of GFP^+^ cells into the maternal circulation (see Methods). Both flow cytometry and immunofluorescence revealed a significant number of CD45^+^GFP^+^ in the placenta fetal layers ***and*** within in the embryonic hearts from NDPI but not control pregnancies (Figure S6A and B). These data were validated by the increased leakage of FITC dextran in NDPI placentas compared to their control counterparts (Figure S6C) and supports the hypothesis that exaggerated placental inflammation promotes a breakdown in placental tissue barrier.

Phenotypic analyses of maternal leukocytes within the embryonic hearts from NDPI pregnancies revealed the presence of T cells, NK cells, neutrophils, monocytes; with the predominant maternal cell type being F4/80^+^Ly6C^hi^ macrophages (Figure 4B), with no difference in MHC II expression between control and NDPI pregnancies, but significant increased expression of proinflammatory chemokine receptors CCR2 (Figure 4C), CCR5 and CXCR4 (Figure S7A). This chemokine receptor expression profile was akin to placental inflammatory F4/80^-^Ly6C^hi^ monocytes from NDPI pregnancies. In line with our GFP cell tracking experiments, these data show that maternal cells migrate across the placental barrier and are recruited to the embryo heart. q-PCR analyses revealed significant increases in gene expression for both CCL3 and CCL4, ligands for CCR5, with a reciprocal attenuation in CXCL12 (Figure S7B) in embryonic hearts from NDPI pregnancies, compared to control; these changes were organ-specific, since no differences were observed in embryonic livers (Fig S7C) and suggest targeted migration of maternal cells specifically to the embryonic heart. There was a significant augmentation in both IL-6 and IL-1 β in NDPI embryonic hearts, but not TNF-α or IL-10 (Figure S7D). Intracellular flow cytometry identified a significant upregulation in the absolute number of maternal, fetal, and non-leukocytes expressing IL-1β in NDPI hearts, and only maternal macrophages and non-leukocytes expressing higher levels of IL-6 (Figure S7E). Altogether, these data indicate a maternal proinflammatory CCR2-driven environment within the embryonic heart interferes with normal development of the organ.

To mechanistically challenge our hypothesis that maternal CCR2^+^ monocytes cause aberrant heart development we investigated the impact of maternal monocyte CCR2 deletion on embryonic heart development in our NDPI model. CCR2^-/-^ females on a B6 background were mated with Balb/C (CCR2^+/+^) males and pregnancies were treated as outlined in Figure S1, with placental tissue, embryos and offspring resulting from these pregnancies being CCR2^+/-^ control or CCR2^+/-^ NDPI. Initial analyses revealed CCR2^+/-^ NDPI placentas were of comparable weight to control (Figure S8A). TNF-α levels from placentas from CCR2^+/-^ control and CCR2^+/-^ NDPI pregnancies were at similar levels to control placentas (Figure S8B). The number of F4/80^+^Ly6C^hi^ inflammatory macrophages and F4/80^-^ Ly6C^hi^ monocytes from CCR2^+/-^ control and CCR2^+/-^ NDPI placentas were analogous to control placentas (Figure S8C&D). This attenuation in inflammation in CCR2^+/-^ NDPI placentas suggests placental inflammation in NDPI pregnancies is maternally driven.

We next sought to look at the development of control and NDPI embryonic hearts. RT(q)-PCR analyses were carried out on key genes involved in embryonic heart development. There was a significant downregulation gene expression in NDPI embryonic hearts compared to control in the following genes: *Gata6* (regulates cardiomyocyte proliferation together with *Gata4*^29^)*; Mef2c* (required for normal endocardium formation^30^)*; Hey2* (involved in normal ventricular wall and myocyte development ^31^)*; Loxl2*, (required for maturation of ECM and normal ventricular septation during development^32^); *Myh6, Myh7* (required for cardiomyocyte contractility^33^)*; Nppa* (during development is restricted to the trabecular myocardium and required for ventricle formation^34^); and *Yap1* (required for vascular wall development through smooth muscle proliferation and cardiomyocyte proliferation^35^) (Figure S8E). Given we see observe a LVNC-like phenotype, the downregulation in *Nppa* was surprising. However, *Nppa* is required for normal ventricle formation during cardiac development^34^. Thus, the downregulation in *Nppa* could account for the ventricular defects that we also observe in our NDPI embryo hearts. Furthermore, recent data suggest downregulation in the *Myh7* gene is associated with LVNC^36^. Thus, the downregulation in *Myh7* expression in NDPI embryonic hearts, could account for the LVNC-like phenotype.

There were significant increases in gene expression of *Gata4, Meft2c, Hey2* and *Nppa* in both CCR2^+/-^ control and CCR2^+/-^ NDPI embryonic hearts compared to controls (Figure S8E). Immunofluorescence revealed normal development with endomucin expression from both CCR2^+/-^ control and CCR2^+/-^ NDPI embryonic hearts at comparable levels to control (Figure 3A and 4D) as well as proliferation of both CD31^+^ endothelial cells and troponin-T^+^ cardiomyocytes (Figure 4E).

We next focused on resident fetal cardiac macrophages, which are CX_3_CR1^+^ and originate from the yolk sac. These comprise of two populations: CCR2^-^ macrophage population that exert pro-angiogenic functions during cardiac development ^37, 38^; and CCR2^+^ macrophage population that have a short life-span within the embryonic and neonatal heart, with currently unknown function^38^. We found no difference in absolute CX_3_CR1^+^ numbers between control and NDPI pregnancies, nor between CCR2^+/-^ control and CCR2^+/-^ NDPI (Figure 4F). However, we observed an increased inflammatory CX3CR1^+^CCR2^+^ phenotype in NDPI embryonic hearts compared to control, both in terms of proportion and absolute macrophages per heart (Figures 4G & 4H). This coincided with a two-fold decrease in the number of proliferating resident fetal CCR2^-^ macrophages (Figure S8F). These data highlight that a skew toward a CX_3_CR1^+^CCR2^+^ heart-resident fetal macrophage phenotype may underlie the dysregulated embryonic heart development observed in NDPI pregnancies.

**Figure 5:**
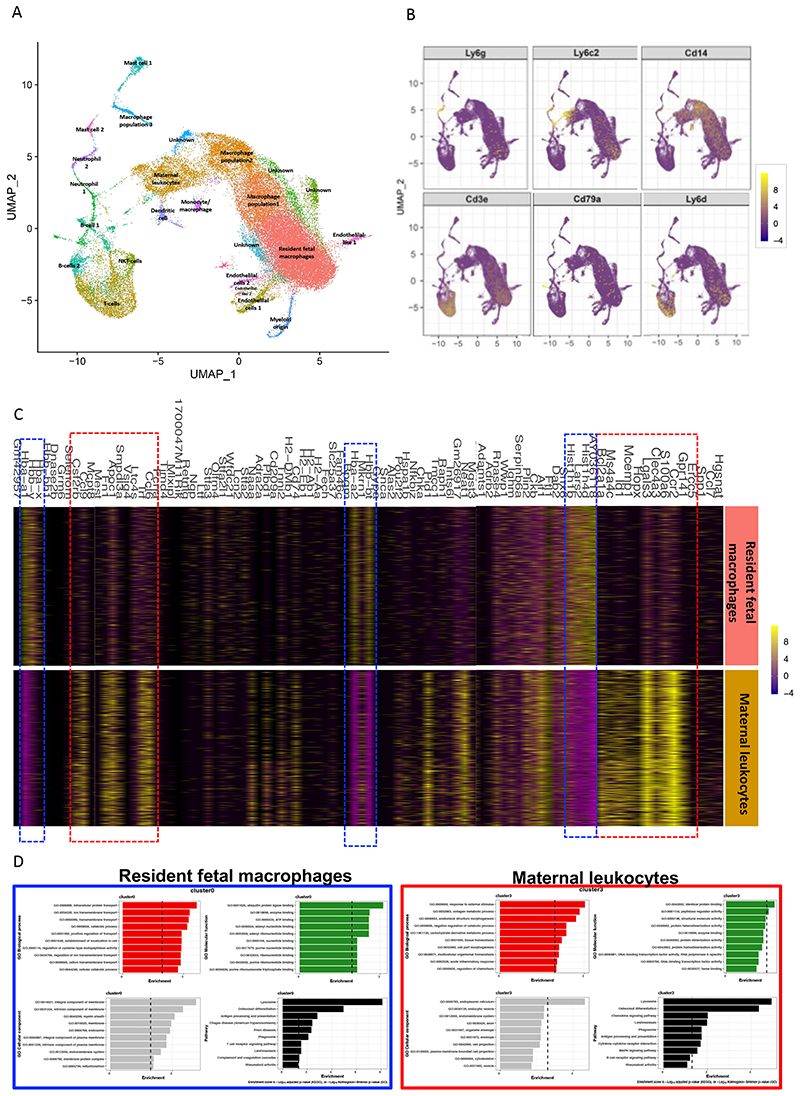
Single-cell sequencing of leukocyte s from E14.5 embryo hearts Neutrophils were depleted at day 4.5 and 7.5 of pregnancy using αLy6G. Mice were sacrificed at E14.5 of pregnancy and hearts dissected from harvested embryos from control or NDPI pregnancies. CD45^+^ cells were isolated from heart single cell suspensions using CD45 PE and anti-PE microbeads. Single cell sequencing was performed on the isolated cells. (A) UMAP showing cell clusters found in hearts of fetuses from control and NDPI pregnancies. (B) Feature plots showing expression of key genes within clusters (C) Heart map analyses comparing gene expression between resident fetal macrophage and maternal leukocyte clusters (D) Enrichment analysis of GO terms and pathways for differentially expressed genes. GO analysis including biological process, cellular component, and molecular function. Pathway analysis based on the KEGG database

To gain a deeper understanding of leukocyte composition and phenotype in the embryonic hearts, we undertook single cell RNA-sequencing (scRNA-seq). CD45^+^ leukocytes were isolated from E14.5 embryonic hearts from control and NDPI pregnancies. UMAP analyses revealed heterogenous leukocyte populations with a mixture of both myeloid and lymphoid cells present (Figure 5A and 5B; Figures S9 and S10). Further analyses of the clusters revealed resident fetal macrophage and maternal leukocyte clusters, expressing distinct genes. For example, resident fetal macrophages expressed many haemoglobin genes including *Hbb-y, Hba-x, Hbb-bt and Hba-a2*, which have been shown to be highly expressed in yolk sac-derived macrophages ^39, 40^ (Figure 5C, blue dotted lines). Genes associated with maternal leukocytes cluster appeared to be associated with adult macrophages, including *Ccr2, Lgals3, S100A6, Hopx, Ms4a4c, Clec4a3, Ccl6, Ccl9* (Figure 5C, red dotted lines). Enrichment analyses also revealed distinct pathways between the resident fetal macrophage and maternal leukocyte clusters. GO Biological Processes analyses of resident fetal macrophages revealed pathways involved in intracellular protein transport, ion transmembrane transport, with the top pathway hit being lysosome (Figure 5D left panel). Indeed, evidence suggests yolk sac macrophage lysosomal activity may be important in regulating fetal testis vascularization and morphogenesis^41^, which may also have important implications in the vascularization of the developing heart. Enrichment analyses of the maternal leukocyte cluster revealed GO Biological Processes associated with adult macrophages, including carbohydrate and collagen metabolism; macrophage-derived collagen has recently been demonstrated to directly contribute to cardiac fibrosis following injury ^42^ (Figure 5D, right panel). Taken together, our sc-RNA-seq data suggest a heterogenous leukocyte population exists within the developing heart and that distinct gene clusters and pathways distinguish between resident fetal macrophages and maternal leukocytes.

### Impaired cardiac development from NDPI pregnancies persists into postnatal life

We next investigated whether the abnormal embryonic heart development detailed at E14.5 continues into postnatal life, assessed at postnatal day 5 (P5) P28 (adult).

Offspring from NDPI pregnancies at P5 had smaller body weights and a higher heart: body weight ratio, when compared to their control counterparts (Figure S11A). We observed a~90% reduction in maternal cells within P5 hearts (Figure S11B). No significant difference in absolute number of resident CX_3_CR1^+^ leukocytes (Figure S11C), yet the proportion of CX_3_CR1^+^CCR2^-^ continued to be significantly lower in offspring hearts from NDPI pregnancies compared to control (Figure S11D). As we observed a LVNC-like phenotype in embryo hearts from NDPI pregnancies at E14.5, (Figure 3), we assessed the cardiac architecture of P5 hearts from both groups, staining cross-sections with DAPI, wheat germ agglutinin (for general architecture) and endomucin. Cardiac tissue of P5 hearts of offspring of NDPI pregnancies displayed hypertrabeculation, with a less compact endocardial structure coupled with a significant reduction in endomucin staining (Figure S11E) and was accompanied with a significant reduction in the number of CD31^+^ endothelial cells (Figure S11F). Postnatally, hearts from CCR2^+/-^ NDPI offspring resembled control P5 hearts (Figure S11G), suggesting maternal CCR2^+^ leukocytes drive the defect in endocardial structure.

**Figure 6:**
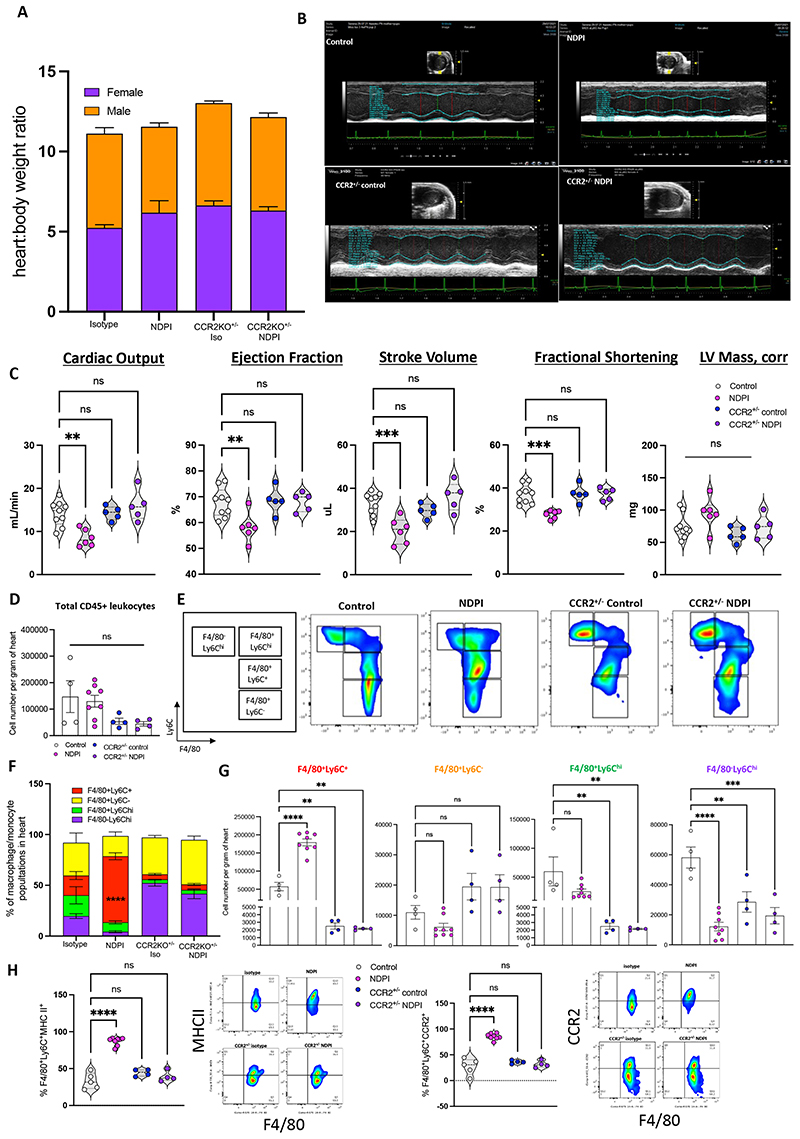
Aberrant embryonic cardiac development from NDPI pregnancies continues into postnatal life Neutrophils were depleted at day 4.5 and 7.5 of pregnancy using αLy6G. Offspring of these dams were sacrificed at post-natal day 5 (P5) or 28 as indicated and heart structure and immune composition assessed. Offspring from Control (white) and NDPI (pink). (A) P28 offspring body weights in grams and heart: body weight ratio from control, NDPI, CCR2^+/-^ control and CCR2^+/-^ NDPI pregnancies (B) Representative echocardiography plots of P28 hearts of offspring from control, NDPI, CCR2^+/-^ control and CCR2^+/-^ NDPI pregnancies (C) Graphs showing quantification of heart parameters determined by echocardiography. (D) Flow cytometric quantification of total CD45^+^ leukocytes from P28 offspring hearts of control, NDPI, CCR2^+/-^ control and CCR2^+/-^ NDPI pregnancies (E) Flow cytometric analyses of macrophage and monocyte populations, as determined by F4/80 and Ly6C staining in P28 offspring hearts from control, NDPI, CCR2^+/-^ control and CCR2^+/-^ NDPI pregnancies (F) Proportion of macrophage and monocyte populations as shown in (E) in P28 offspring hearts from control, NDPI, CCR2^+/-^ control and CCR2^+/-^ NDPI pregnancies (G) Absolute number per gram of heart tissue of monocyte and macrophage populations shown in (E) in P28 offspring hearts from control, NDPI, CCR2^+/-^ control and CCR2^+/-^ NDPI pregnancies (H) Percentage F4/80^+^Ly6C^+^ MHCII^+^ (left panel) and F4/80^+^Ly6C^+^ CCR2^+^ in P28 offspring hearts from control, NDPI, CCR2^+/-^ control and CCR2^+/-^ NDPI pregnancies Each symbol represents an individual mouse and statistical significance was tested by One -way ANOVA, with Dunnett’s multiple comparison test, Brown-Forsythe ANOVA test, with Dunnett’s post comparison (G F4/80^+^Ly6C^+^), compared to control. Ns = not significant, *p≤0.05, ** p≤0.01, ***p≤0.001 **** p≤0.0001. In all cases, data are mean ± SEM.

No difference in the heart: body weight ratios between all four mouse groups was observed regardless of the sex of the mouse (Figure 6A) at P28. Echocardiography of male and female adult offspring from NDPI pregnancies revealed a significant attenuation of cardiac output, stroke volume, ejection fraction and fractional shortening, with no difference in left ventricle mass, compared to control; while both CCR2^+/-^ control and CCR2^+/-^ NDPI offspring displayed normal cardiac function (Figure 6B and 6C). We next investigated the phenotype of cardiac macrophages in adult offspring hearts. No overall difference was observed in the total number of CD45^+^ leukocytes within the cardiac tissue across all four groups (Figure 6D). We assessed monocyte and macrophage subsets with respect to F4/80 and Ly6C namely three macrophage populations: F4/80^+^Ly6C^+^, F4/80^+^Ly6C^hi^ and F4/80^+^Ly6C; as well as the monocyte population F4/80^-^Ly6C^hi^. The proportion and number of F4/80^+^Ly6C^+^ macrophages were significantly increased in P28 hearts of NDPI offspring (Figure 6F and 6G), coupled with a significant proportion of this subset expressing MHCII and CCR2 (Figure 6H). We also observed a significant attenuation in the number of F4/80^+^Ly6C^+^, F4/80^+^Ly6C^hi^ and F4/80^-^Ly6C^hi^ populations in both CCR2^+/-^ control and CCR2^+/-^ NDPI offspring hearts compared to control (Figure 6G), adding support that this chemokine receptor is important in maintaining a pro-inflammatory environment within the cardiac tissue^43^

### Quelling placental inflammation prevents abnormal cardiac development

Based on the above data, we propose a model where a breakdown in the placental tissue barrier, because of high local inflammation, promotes cardiac-selective influx of proinflammatory maternal leukocytes which impacts on normal cardiac development. To address this hypothesis, we targeted TNF- α-mediated placental inflammation by injecting NDPI pregnant mice with a neutralizing anti-TNF-α antibody (referred hereafter as NDPI+aTNF-α; see Figure S12A for scheme). Blocking TNF-α reduced placental TNF-α levels and i) rescued the poor placental tissue architecture, ii) enabled deeper trophoblast invasion and iii) restored placenta collagens at both gene and protein level (Figure S12B-E).

**Figure 7:**
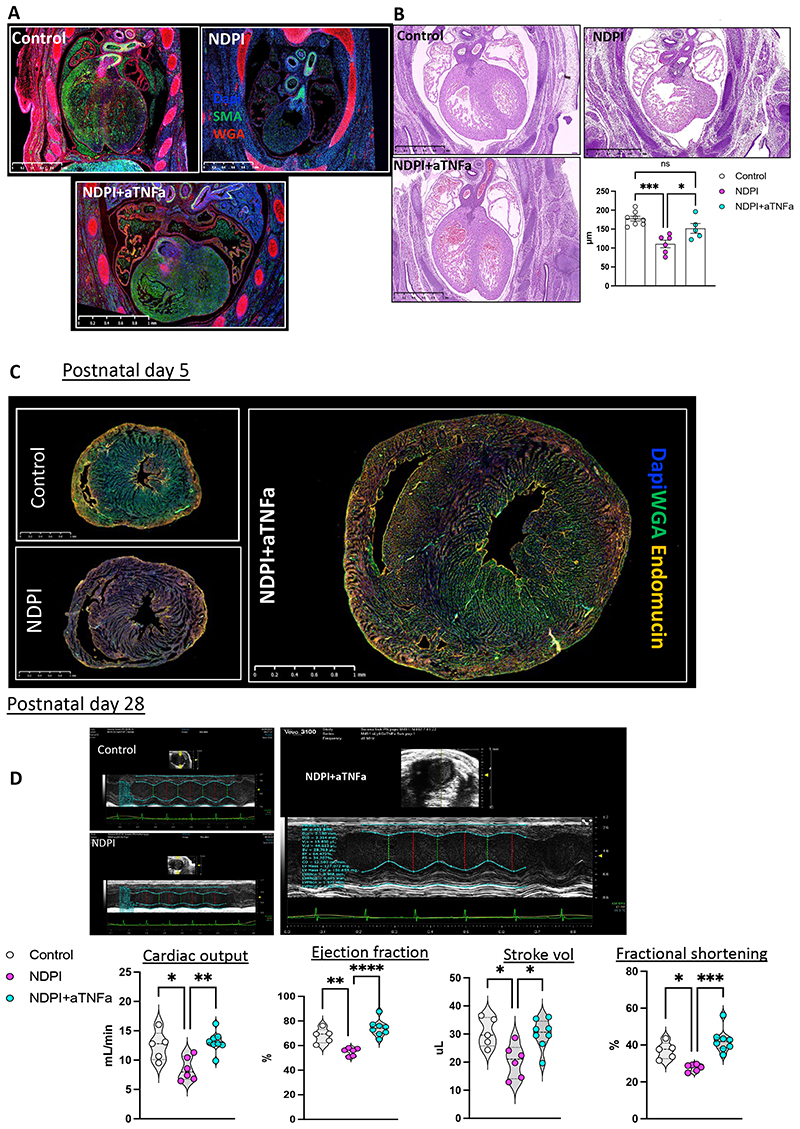
Quelling placental inflammation prevents abnormal cardiac development Neutrophils were depleted at day 4.5 and 7.5 of pregnancy using αLy6G. At day 8.5 of pregnancy TNFα was neutralized by injecting αTNFα IgG i.v. Mice were sacrificed at E14.5 of pregnancy and placentas and embryos harvested to assess the structure and immune composition. Control (white), NDPI (pink), neutrophil depleted and TNFα neutralized (aTNF-αNDPI; blue). (A) Immunofluorescent images of E14.5 embryo heart of cross section. Sections were stained with DAPI (blue) WGA (red) SMA (green). Graph showing ventricle wall thickness quantification. (B) H&E sections of E14.5 embryonic hearts from aTNF-αNDPI pregnancies compared to control Graph represents LV wall thickness (C) Immunofluorescent images showing the expression of endomucin (orange) and WGA (green) in cross sections of hearts from P5. Scale bar 1mm. Magnified images showing endomucin staining of the ventricle. Graph showing the proportion of total CD31^+^ cells in P5. (D) Echocardiography plot with graphs (bottom panel) showing quantification of heart parameters determined by echocardiography. Each symbol represents an individual mouse and statistical significance was tested by One-way with Bonferroni post-correction. ns = not significant, *p≤0.05, ** p≤0.01, ***p≤0.001 **** p≤0.0001. In all cases, data are mean ± SEM.

Fewer activated placental neutrophils were counted, expressing lower levels of TNF-α, CXCR2, CD114 and MMP9 from NDPI+aTNF-α pregnancies compared to NDPI, coupled with reduced numbers of inflammatory monocytes (Figure S12F and S12G). Following maternal aTNF-α treatment, fewer maternal cells were recruited into the embryonic hearts (Figure S12H) and the proportion of CX_3_CR1^+^CCR2^-^ resident fetal macrophages increased when compared to both control and NDPI pregnancies (Figure S12I). This was coupled with a downregulation in both IL-1β and IL-6 gene expression (Figure S12J). These cellular changes yielded a well-defined cardiac structure, resembling the structure of control embryonic hearts (Figure 7A) including left ventricle wall thickness (Figure 7B) and a restoration in the proliferation of both CD31^+^ endothelial cells and cardiomyocytes (Figure S12K).

Postnatally (P5), we observed a ~two-fold increase in the proportion of CX_3_CR1^+^CCR2^-^ macrophages relative to CX_3_CR1^+^CCR2^+^ cells, in NDPI+aTNF-α compared to P5 hearts from NDPI offspring (figure S12L). Heart: body weight ratios from offspring of NDPI+a TNF-α were akin to control offspring (Figure S12M). We also observed a loss in the hypertrabeculation displayed by NDPI pregnancies, and a more compact endocardium within the cardiac tissue, accompanied by a significant increase in CD31 ^+^ endothelial cells in NDPI+a TNF-α offspring (Figure 7C).

Finally, we observed a restoration in cardiac function of adult (P28) hearts of NDPI+a TNF-α offspring compared to their NDPI counterparts (Figure 7D), which coincided with fewer F4/80^+^Ly6C^+^ macrophages within the cardiac tissue. Cardiac macrophages displayed a more quiescent, anti-inflammatory phenotype, expressing significantly lower levels of MHCII and CCR2 compared to their NDPI counterparts (Figure S12N).

Taken together these data suggest tempering neutrophil-driven placental inflammation with TNF-α neutralization is sufficient to rescue cardiac development and function in offspring postnatally and into adulthood.

## Discussion

We describe how placental inflammation, driven by activated neutrophils, promotes aberrant embryonic heart development that impacts cardiac structure and downstream cardiac function in postnatal life. Specifically, locally produced neutrophil-TNF-α promotes the establishment of an inflammatory placental environment with breakdown of tight tissue barriers; this, in turn, allows the transfer of placental inflammatory maternal monocytes to the embryonic heart, impeding its normal development, leading to persistent heart dysfunction.

Following neutrophil depletion, an activated circulating neutrophil phenotype emerges, with high levels of CXCR2 and GCSF receptor CD114. Consequently, activated neutrophils are present within the placental tissue, coupled with high expression of TNF-α and MMP9. This neutrophil-driven inflammatory environment is responsible for matrix degradation of the placentas, with repressed collagen gene and protein expression.

The complex structure of the placenta barrier ensures maternal leukocytes do not enter the fetal compartment, and this guarantees the tolerogenic state of the placenta^44^. However, maternal cells, can cross the barrier and *via* the fetal circulation (called maternal microchimeric cells), enter fetal organs including liver, lung, pancreas and heart^45^. A recent study identified maternal microchimeric cells in cardiac tissue of infants who died from neonatal lupus with heart block^46^, suggesting cells of maternal origin may reach the heart to impact on cardiac responses in offspring. Utilizing the CD45.1 (maternal) and CD45.2 (paternal) system, as well as GFP leukocyte transfer experiments, we identified a significant increase in the number of maternal (CD45.1^+^CD45.2^-^) inflammatory macrophages in embryonic hearts from NDPI pregnancies. The phenotype of the maternal cells is akin to the maternal inflammatory monocytes detected in the placenta. Analyses of chemokines in embryonic hearts and livers identified an embryonic heart-specific (but not liver-specific) chemokine ligand profile resulting from NDPI pregnancies (namely CCL3, CCL4), which matched reciprocal chemokine receptors on maternal cells both in placenta and embryonic heart. This raises the question of what may be responsible for altering the environment of the developing heart? The timing of both neutrophil depletions in our model precedes maternal inflammatory immune cell infiltration since the 1^st^ depletion is carried out at E4.5, which is prior to both placenta and heart development, and the 2^nd^ depletion at E7.5, at the beginning of placental and heart development^47^. This suggests manipulation of the maternal quiescent neutrophil response in early gestation may prime the placenta and - subsequently – the embryonic heart to a pro-inflammatory environment.

Placental development occurs in parallel to embryonic cardiac development and studies have highlighted the reciprocal influence these organs exert on each other’s development^5,48^. Indeed, evidence suggests women who have preeclampsia (PE) during their pregnancy have a significant increased risk of their fetuses developing a CHD^8^, attributed to the poor placental development that occurs in PE pregnancies, including poor trophoblast invasion, and aberrant oxygen and nutrient transfer from the mother ^7^. There may also be a genetic link between placental development in early-onset PE and the development of fetal CHDs, where epigenetic programming of placental and fetal tissues leads to an increase of cardiac anomalies in offspring of PE pregnancies^49^.

Embryonic hearts from NDPI pregnancies display hypertrabeculation and thinner left ventricular walls, reminiscent of the congenital heart condition left ventricular non-compaction^27^. Elegant studies have demonstrated recently that resident cardiac fetal macrophages populate distinct regions of the developing heart: while CX3CR1^+^CCR2^-^ macrophages predominantly populate the myocardial wall, CX3CR1^+^CCR2^+^ macrophages are found in the trabecular projections^11^, however the exact function during embryonic heart development of this latter subtype is currently unclear. Noteworthy, the presence of CCR2^+^ macrophages within the embryonic and neonatal heart is short lived, whereas their CCR2^-^ counterparts are self-renewing^12, 38^. The current data add another layer of complexity to this dichotomy, whereby presence of inflammatory maternal cells within the developing heart can promote the induction of these CCR2^+^ macrophages. In adult cardiac tissue, the roles of CCR2^-^ and CCR2^+^ macrophages are more apparent: resident CCR2^-^ macrophages of fetal origin have a reparative function, whereas CCR2^+^ monocyte-derived macrophages promote myocardial injury and inflammation^43^. Our findings indicate that, within the embryonic heart, maternal inflammatory cells can skew toward a fetal CCR2^+^ inflammatory macrophage phenotype, that coincides with a distinct LVNC-like tissue architecture. These CCR2^+^ fetal macrophages may function to promote embryonic cardiac injury *or* they may create an inflammatory environment which subsequently impacts heart development; this may suggest why these cells are short-lived under normal heart developmental conditions. Postnatally, while there was no presence of inflammatory maternal cells in offspring hearts, an inflammatory cardiac macrophage phenotype persisted in P5 and P28 hearts from offspring of NDPI pregnancies, coupled with poor cardiac tissue architecture and function. Altogether, these data indicate that the influence of inflammatory maternal cells in embryonic heart development is imprinted in postnatal life and adds support to the concept that the innate immune competency of the placenta is key to the leukocyte composition of the developing heart.

In summary, we present a mechanistic paradigm whereby neutrophil-driven inflammation in pregnancy can preclude normal embryonic heart development as a direct consequence of poor placental development. Importantly, this study also opens translational avenues for early diagnoses and potential treatment of CHDs in utero. The former assertion is evidenced by the finding that NDPI pregnancies associate with an activated neutrophil phenotype within the maternal circulation. Relevantly, women with preeclampsia (PE) present an activated neutrophil phenotype^14, 16^ and may produce a higher incidence of CHDs in offspring^50^. Thus, early phenotyping of neutrophils from pregnant women could provide an early diagnostic test to identify CHDs in fetuses. Finally, early identification of placental inflammation could be mitigated through a therapeutic intervention like anti- TNF-α used here. Thus, anti-inflammatory therapy can restore normal embryonic and postnatal heart development and function, offering a potential alternative to current invasive in utero interventions to treat CHDs.

## Supplementary Material

Supplemental Material File

## Figures and Tables

**Table 1:** Demographics of pregnant patients from which placental tissue was assessed Patient demographics of placentas characterized in Figure 2F. Words in Bold highlight each patient demographic. CHD= fetal congenital heart disease

Pregnancy type	Age of Mother	Parity	Gestation (weeks)	Blood pressure	Mode of delivery
Healthy	18	0	41	Within normal range	Vaginal
Healthy	28	0	41	Within normal range	Vaginal
Healthy	31	0	39	Within normal range	Vaginal
Healthy	32	3	40	Within normal range	Vaginal
Healthy	40	5	37	Within normal range	Vaginal
Pre-eclampsia	30	2	37	190/110	C-section
Pre-eclampsia	22	0	37	171/106	C-section
Pre-eclampsia	32	2	36	178/113	C-section
Pre-eclampsia	32	2	38	190/120	C-section
Pre-eclampsia	22	0	40	192/101	Vaginal
Fetal CHD: D-transportation of great arteries	39	0	39	Within normal range	C-section
Fetal CHD: Ventricular septal defect	28	0	38	Within normal range	C-section
Fetal CHD: Tetralogy of Fallot	33	0	35	Within normal range	C-section
Fetal CHD: Ventricular septal defect	36	0	40	Within normal range	C-section

## Clinical Perspectives

### What is new?

- Placental inflammation leads to loss of barrier function that negatively impacts embryonic heart development
- Placental CCR2+ maternal inflammatory monocytes/macrophages impede resident cardiac fetal macrophage phenotype and function
- Directly targeting placental TNF-alpha rescues defective cardiac development

### What are the Clinical Implications?

- Potential for Placental inflammation to be used as a diagnostic tool to evaluate fetal risk of CHDs
- Targeted placental anti-inflammatory therapy offers an alternative clinical approach to current invasive in utero interventions
