# Supplementary material for: Placental inflammation leads to abnormal embryonic heart development
Source: Circulation. Author manuscript; Available in PMC 2023 Mar 20. (PMC10022676; doi:10.1161/CIRCULATIONAHA.122.061934)
Supplement: Supplemental Material File [file EMS157819-supplement-Supplemental_Material_File.pdf]

Supplemental Materials

Extended Methods

Human Samples

This study was carried out following the Declaration of Helsinki, was approved by the Research Ethics Committee of the University of Southern Santa Catarina (UNISUL) under 34681920.8.0000.5369 (for Preeclampsia samples - approved in 20/08/2020) or CAAE: 36084720.9.0000.5369 (for cardiac defects - approved in 18/01/2021) and all participants signed written informed consent and collected samples have been anonymised.

Animals

Female C57/Bl6J mice and male Balb/C mice were purchased from Charles River UK. CD45.1 (B6.SJL-Ptprca Pepcb/BoyJ) were originally purchased from Charles River Italy and C57BL/6-Tg(CAG GFP)131Osb/LeySopJ mice from The Jackson Laboratory, respectively. CCR2-deficient (B6.129S4-Ccr2tm1Ifc/J; JAX stock #004999)(Boring et al., 1997) were originally purchased from Jackson laboratory but were provided as a generous gift from Dr Gerard Graham, University of Glasgow. All mice were housed at the animal facility of Queen Mary University of London and were conducted with strict adherence to the Home Office guidelines (PPL P71E91C8E) following approval by the Animal Use and Care Committee of Queen Mary University of London (QMUL). Mice used in the experiments of this study, unless specified were used at 8- 12 weeks old

Timed-mated allogeneic pregnancy

Timed mating was performed by housing Balb/C male mice with aged-matched female C57BL/6J or CD45.1 mice overnight and confirmed by the presence of a copulatory plug the following morning; this was defined as day 1 or mouse embryonic stage 0.5 (E0.5). Circulating maternal neutrophils were depleted at days 5 (E4.5) and 8 (E7.5) of pregnancy using a monoclonal neutralizing antibody (Biolegend, clone 1A8; 50 µg

i.v.) or isotype control (Biolegend clone RTK2758; 50µg i.v). Following neutrophil depletion, some mice were injected with anti-mouse TNF- α i.v (Biolegend clone MP6-XT22; 10mg/kg i.v) on day 9 of pregnancy. Pregnant females were sacrificed at either day 15 of gestation (E14.5) or left to give birth and offspring were sacrificed at post-natal day 5 (P5) or post-natal day 28 (P28).

***In vivo* BrdU uptake**

Mice were injected with 100mg/kg 5-Bromo-2′-deoxyuridine (BrdU) i.v (Sigma; B5002-1G) and culled 1 hour later. Single cell suspension were prepared from the required organs and stained with FITC BrdU staining kit (Invitrogen; 8811-6600-42) according to the manufacturer's instructions.

Transfer of GFP$^+$ leukocytes into pregnant females

$5X10^6$ Splenic leukocytes from GFP$^+$ age-matched females were injected intravenously into either isotype or NDPI pregnant females. 18 hours after injections, pregnant mice were culled and detection of GFP$^+$ leukocytes within the placentas and E14.5 embryonic hearts via flow cytometry and immunofluorescence.

Placenta permeability assay using FITC dextran

To test the permeability of placentas, 10mg/ml of FITC-dextran in PBS (20,000 mW, Sigma Aldrich) was injected intravenously in pregnant isotype control or NDPI mice for 15 minutes. Mice were then culled, and placentas were immediately fixed in 4% paraformaldehyde for two hours followed by 70%ethanol, dehydrated in 30% sucrose, and embedded in OCT for imaging. Wide field imaging was carried out using Nanozoomer S60 slide scanner (Hamamatsu).

Placental neutrophil isolation and splenic monocyte *in vitro* co-culture

Placentas obtained from E14.5 pregnant mice were cut into small fragments and digested in 1XPBS with 20mM HEPES, 60 U/ml DNase I and 450 U/ml Collagenase I at 37°C and 250rpm for 20 minutes. Suspensions were passed through 70µm filters, washed and red blood cells lysed with ACK lysis buffer. Placental neutrophils contained in the resulting single cell suspension were enriched via positive selection using 0.2µg anti-mouse Ly6G PE (Biolegend, clone 1A8) per $1x10^6$ cells followed by

anti-PE microbeads (Biolegend), used according to the manufacturer's instructions. The enriched placental neutrophil population were then used in downstream applications.

Monocytes, from spleens of non-pregnant female C57BL/6J mice were isolated using a commercially available negative selection kit according to manufacturer's instructions (EasySep mouse monocyte isolation kit). $1x10^5$ placental neutrophils were co-cultured with $1x10^5$ splenic monocytes in DMEM (Gibco) growth media containing 10% FCS and 50 IU/mL penicillin, 50 µg/mL streptomycin and supplemented with 10ng/ml M-CSF (Biolegend; cat#576402) at time of culture and again at day 3 of culture. Cells were cultured for a total of 5 days.

Flow cytometry and antibodies

Single cell suspensions were prepared E14.5 embryo hearts, E14.5 embryo livers and P5 hearts through 70µm cell strainers. For placentas (see neutrophil isolation methods) and P28 hearts, enzymatic digestion was used to obtain single cell suspensions. Briefly, P28 hearts were digested using 450U/ml Collagenase I, 125 U/ml Collagenase XI, 60U/ml Dnase1 in 1XPBS containing 20mM HEPES. In both cases, tissues were digested for 20 minutes, at 37C in a shaking incubator at 125 RPM.

Dead cells were excluded using a fixable viability dye LIVE/DEAD fixable aqua (Thermo Fisher Scientific, L34957). Cells were gated on singlets (See Figure S12). Cells were then further phenotype as indicated in the results. Representative FACS plots show initial gating strategy for singlets and live cells.

Immunofluorescence microscopy

Tissues were fixed in 4% paraformaldehyde for 2 hours and paraffin or OCT embedded. Paraffin embedded sections were de-paraffinised with Histoclear and re-hydrated prior to antigen-retrieval performed by heating sections to 95^oC for 10 min in Abcam antigen retrieval solution (ab64236). Sections were stained for hematoxylin/eosin or incubated with primary antibodies for immunofluorescence staining and mounted with Fluoromount G (Thermofisher 00-4958-02)

Nuclei were visualized using DAPI. Wide field imaging (bright field and immunofluorescence) was carried out using Nanozoomer S210 or S60 slide scanner

(Hamamatsu). Confocal microscopy was carried out on a Carl Zeiss LSM800. Images were analysed with ImageJ (NIH) and Volocity (Version 6.3, PerkinElmer).

High resolution episcopic microscopy (HREM)

Samples for high resolution episcopic microscopy (HREM) were fixed in Bouin's for a minimum of 12h followed by extensive washing in PBS, dehydration in graded methanol series, incubation in JB-4 (Sigma) /Eosine (Sigma)/Acridine orange (Sigma) mix overnight to ensure proper sample infiltration and then embedded in fresh mix by adding the JB4 accelerator. Once polymerized the block where imaged as previously described[15]. Samples were sectioned on a Leica sledge microtome at 1 or 2 µm or on commercial oHREM (Indigo Scientific) at 0.85 or 1.7 µm. An image of the surface of the block was then acquired under GFP excitation wavelength light using Olympus MVX10 microscope and High-resolution camera (Jenoptik). After acquisition the stacks were adjusted for gray level using Photoshop CS6 and then process for isotropic scaling, ortogonal re-sectioning, 25% downscaling, using a mixture of commercial and homemade software (see Wilson R et al. NAR 2016, Vol. 44 D855-D861). 3D volume rendering of the datasets were produced from the 25% downscale stack using Horos. Quantification of number and length of LV trabeculae were measured using Fiji imaging software[16] Number of trabeculae were measured at the point of invagination within the endocardial wall and were made from 2D images obtained from the 3D rendering by two independent researchers.

Cytokine analysis

Placenta digest supernatants and plasma concentrations of TNF-α (Peprotech, 900-TN54) and IL-1β (Biolegend, 432601) were measured by ELISA according to the manufacturer's instructions.

***In Vivo* Cardiac Functional Assessments**

Echocardiography was performed on offspring at postnatal day 28 to assess cardiac structure and function using a Vevo-3100 imagining system (VisualSonics, Toronto, Ontario, Canada). Mice were anaesthetized (3% isoflurane in 0.2L/min oxygen, maintained with 2% isoflurane and 0.4L/min) and body temperature maintained at 37 °C. Heart rate was monitored for the duration of the procedure. Images from two-

dimensional brightness mode (B-mode) and one-dimensional motion mode (M-mode), were obtained. Left ventricular ejection fraction (EF%), fractional shortening (FS%), cardiac output and stroke volume measurements were obtained from short-axis M-mode. Values were averaged from three beats. Echocardiographic analyses were performed by two independent operators, using Vevo LAB dongle software V3.2.6.

Quantitative Real-time polymerase chain reaction

Placenta, fetal heart and fetal liver samples were stored in RNAlater (Invitrogen) at -80°C until processing. 30mg of placenta tissue was disrupted using a sample grinding kit (GE healthcare) and RLT buffer. Fetal heart and liver tissues were disrupted using a syringe and 25G needle and RLT buffer containing 2-Bme. RNA from the disrupted placental tissue and fetal heart and liver tissue was extracted using the RNeasy mini kit, according to the manufacturer's recommendations. RNA quality and quantity was assessed using absorption measurements (Roche, Nanodrop) prior to performing the reverse transcription reaction, as per the manufacturer's instruction (BioRad, iScript™ cDNA synthesis kit). qPCR was performed using 10ng cDNA, 1µM PCR primers (see table below for sequences) and 1x SYBR GreeN PCR mastermix (BioRad) in a CFX connect light cycler (BioRad). Expression was calculated according to the method outlined by Vandesompele *et al* [17]whereby data is normalized to two housekeeping genes, HPRT and RPL32A. The thermal cycling conditions were 95°C for 30 seconds and 40 cycles of 95°C for 5 seconds followed by 60°C for 30 seconds and then melt curve analysis 95°C for 5 seconds, 65°C for 5 seconds and 95°C for 5 seconds. Experiments were performed in duplicate.

Bulk RNA sequencing

High-quality RNA was extracted from 3 individual placenta from. Each treatment group, isotype (control) and aLy6G (NDPI) using Qiagen RNA extraction kit (Qiagen, Germany) and submitted to Eurofins (Germany) for Illumina sequencing. All raw data has been deposited on Figshare (https://figshare.com/s/8b13463311cf442e9d15). Please see the analysis pipeline at github.com/kerrimalone/aLy6G_RNAseq. Briefly, read quality was assessed with FastQC (v0.11.9)(http://www.bioinformatics). Transcript quantification was performed using Salmon (v.0.14.2) and the *Mus musculus* reference transcriptome (GRCm38, Gencode, release M23)[18]. Transcripts

were summarized at the gene level, genes displaying expression levels below one count per million (CPM) in at least 3 individual libraries were filtered out. Differential expression (DE) analysis was performed between NDPI and control using EdgeR [19][3]. A False-Discovery Rate (FDR) threshold (≤0.05, Benjamini-Hochberg) was set to define DE genes. Positive log2 fold change indicates that a gene is higher expressed in aLyG6-treated samples and vice versa. Cellular functions and pathways over-represented for the DE gene list were assessed using the SIGORA in R[20].

Fetal heart CD45$^+$ cell isolations

Hearts from fetuses at E14.5 were dissected and passed through a 70µm cell strainer to obtain a single cell suspension. Heart CD45$^+$ cells were enriched via positive selection using 0.2µg anti-mouse CD45 PE (Biolegend, clone 30-F11) per 1x10^6 cells followed by anti-PE microbeads (Biolegend), used according to the manufacturer's instructions. The enriched fetal heart CD45$^+$ cells were subsequently used for single cell RNA sequencing.

Single cell sequencing

Fetal heart CD45 cells were fixed using 80% methanol and stored at -80°C according to a GENEWIZ-developed protocol. Upon shipping to the Genewiz facility cryopreserved cells were thawed, washed, and counted following the 10x Genomics protocol. Cell suspensions were loaded onto the 10x Genomics Chromium Controller targeting ~10,000 cells per sample for processing and microdroplet generation. The resulting libraries were sequenced with the Illumina® HiSeq and resulted in 52,874 reads on average per cell, per sample. Primary data analyses were conducted in R v4.0.3 following the analysis strategy outlined https://www.ncbi.nlm.nih.gov/pmc/articles/PMC8238499/. Raw BC count matrices were imported to Seurat v4 with min.cells 3 and min.features 400. Prior to normalization, each sample was downsampled randomly to 50000 cells. Each sample was then normalised via SCT transformation. Prior to integration, 2000 variable features were identified using selection.method 'vst'. For identifying integration anchors, 2000 anchor features were identified via the cca method - other parameters were idims 1:30, k.anchor 5, k.filter 200, and k.score 30. For the final integration, the following parameters were set: idims 1:30 and k.weight 100. Prior to downsteam analysis, cells with mitochondrial gene expression > 10% were filtered out, as were

those with nCount_RNA > 40000 and nFeature_RNA > 5000. Clusters were identified in the integrated data using default parameters for FindNeighbors and FindClusters. Markers for each cluster were identified using FindMarkers with parameters logfc.threshold 0.0, test.use 'LR', min.pct 0.25, and min.diff.pct -Inf. Markers per cluster, per treatment were identified using FindAllMarkers and parameters logfc.threshold 0.0, test.use 'LR', min.pct 0.0, and min.diff.pct = -Inf. Volcano plots were generated via EnhancedVolcano. Gene Set Enrichment Analysis was performed via fgsea. Gene Ontology (GO) and KEGG enrichment was performed with topGO and KEGGprofile, respectively. Gene enrichment was also performed using enrichR. Further plots were generated via scDataviz. Cluster annotations were identified using top expressed genes in each cluster as shown in heat map in figure S9 (see also table in Figure S10)

All raw data has been deposited on Figshare (https://figshare.com/s/98321569e7f6a15aff65 and https://figshare.com/s/83bf3f8ba06d4884d827).

Data availability

All RNA seq and Single cell-sequencing raw data can be found via Figshare:

https://figshare.com/s/8b13463311cf442e9d15

https://figshare.com/s/98321569e7f6a15aff65

https://figshare.com/s/83bf3f8ba06d4884d827

All other data are available upon request to the corresponding author

Figure S1

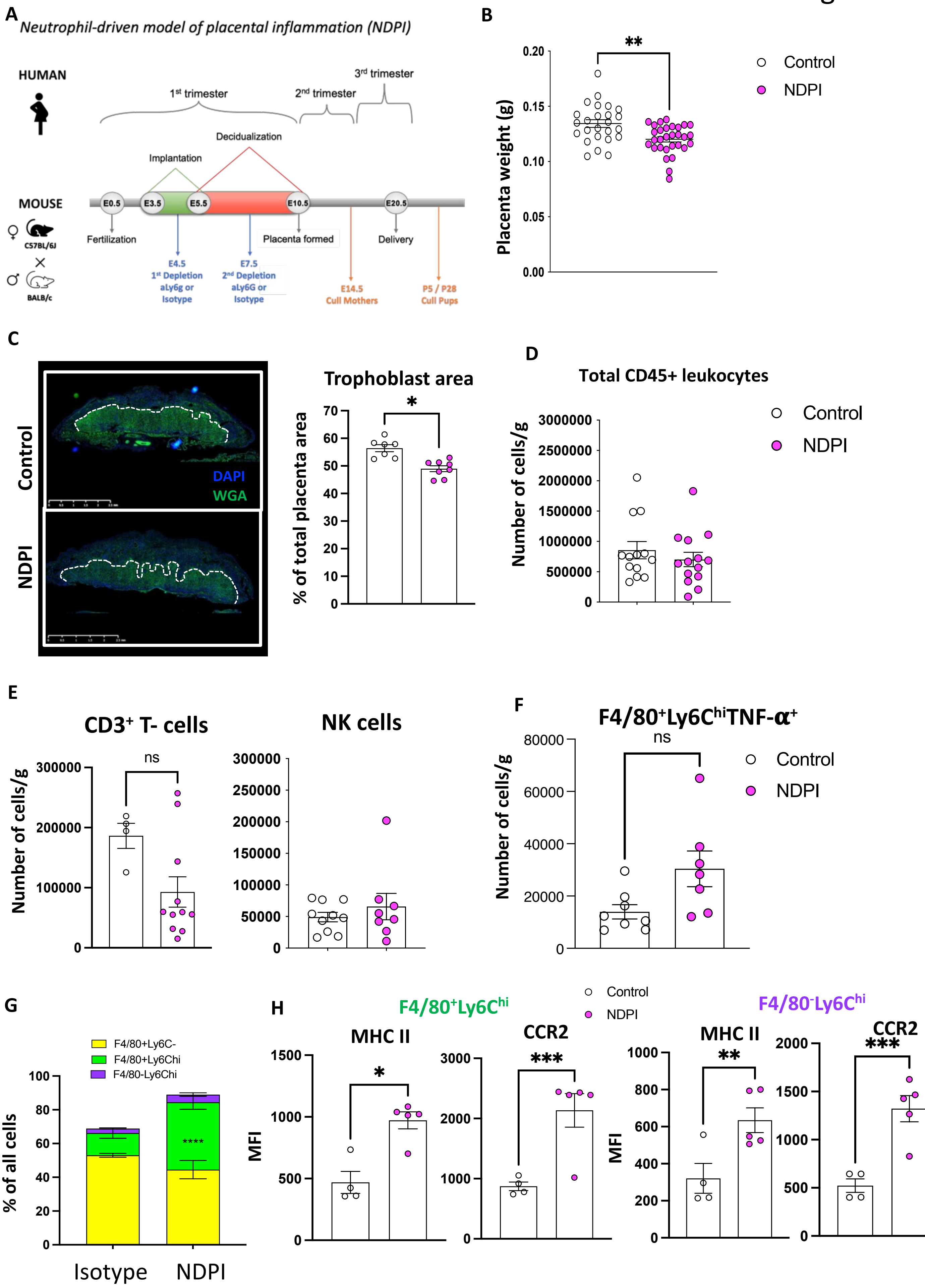
A
Neutrophil-driven model of placental inflammation (NDPI)
HUMAN
1st trimester
2nd trimester
3rd trimester
Decidualization
Implantation
MOUSE
C57BL/6J
BALB/c
E0.5
E3.5
E5.5
E10.5
E20.5
Fertilization
Placenta formed
Delivery
E4.5
1st Depletion
aLy6g or
Isotype
E7.5
2nd Depletion
aLy6G or
Isotype
E14.5
Cull Mothers
P5 / P28
Cull Pups
B
Placenta weight (g)
**
Control
NDPI
C
Control
NDPI
DAPI
WGA
Trophoblast area
% of total placenta area
*
D
Total CD45+ leukocytes
Number of cells/g
Control
NDPI
E
CD3+ T- cells
ns
NK cells
Number of cells/g
F
F4/80+Ly6ChiTNF-α+
ns
Number of cells/g
Control
NDPI
G
F4/80+Ly6C-
F4/80+Ly6Chi
F4/80-Ly6Chi
% of all cells
****
Isotype
NDPI
H
F4/80+Ly6Chi
MHC II
CCR2
MFI
*
***
Control
NDPI
F4/80-Ly6Chi
MHC II
CCR2
**
***

Supplementary Figure 1

Control treated (white) and neutrophil depleted (NDPI) (pink).

(A) Schematic for experimental design

(B) Weights of E14.5 placentas.

(C) Immunofluorescent staining of placentas for wheat germ agglutinin (WGA) (green) to show gross structure and cell nuclei with DAPI (blue). White dotted line indicates the separation of the labyrinthine zone/trophoblast area and the outer junctional zone. Graph showing the proportion of the total area of the placentas occupied by the trophoblast zone. Scale bar =2.5mm

(D-F) Flow cytometry quantification of different leukocyte subsets in placenta, expressed as cell number per gram of tissue (D) $CD45^+$ cells (E) $CD3^+$ total T cells, $NK1.1^+$ NK cells, (left to right) (F) $F4/80^+$ $Ly6c^{hi}TNF\text{-}\alpha^+$ macrophages.

(G) Proportion of $F4/80^+$ $Ly6C^-$, $F4/80^+Ly6C^{hi}$ and $F4/80^-Ly6C^{hi}$ cells following *in vitro* co-culture of isolated neutrophils from isotype of NDPI placentas with naïve splenic monocytes from non-pregnant female mice

(H) $F4/80^+Ly6C^{hi}$ and $F4/80^-Ly6C^{hi}$ co-cultures described in (G) were analysed for the expression of MHCII and CCR2, expressed as median fluorescent intensity.

Each symbol represents an individual mouse and statistical significance was tested by Student's unpaired t-test. ns = not significant, *p≤0.05, ** p≤0.01, ***p≤0.001 **** p≤0.0001. In all cases, data are mean ± SEM. MFI, median fluorescence intensity

Figure S2

A

Table 1 showing all 44 mouse collagens and their expression following RNA-seq analyses

Ensembl ID	Gene Symbol	Gene Name	Collagen Type	Adj P val	Log2FC
ENSMUSG00000001506	Col1a1	collagen, type I, alpha 1 (Col1a1), mRNA	type I	3.27E-14	3.188074
ENSMUSG00000029661	Col1a2	collagen, type I, alpha 2 (Col1a2), mRNA	type I	3.45E-13	2.874913
ENSMUSG00000022483	Col2a1	collagen, type II, alpha 1 (Col2a1), mRNA	type II	3.6E-13	4.937598
ENSMUSG00000026043	Col3a1	collagen, type III, alpha 1 (Col3a1), mRNA	type III	0.442993343	0.681686
ENSMUSG00000031502	Col4a1	collagen, type IV, alpha 1 (Col4a1), mRNA	type IV	0.995426174	0.090912
ENSMUSG00000031503	Col4a2	collagen, type IV, alpha 2 (Col4a2), mRNA	type IV	0.995426174	0.103611
ENSMUSG00000079465	Col4a3	collagen, type IV, alpha 3 (Col4a3), mRNA	type IV	0.996574685	0.014233
ENSMUSG00000067158	Col4a4	collagen, type IV, alpha 4 (Col4a4), mRNA	type IV	0.989220301	0.464417
ENSMUSG00000031274	Col4a5	collagen, type IV, alpha 5 (Col4a5), mRNA	type IV	3.12E-14	3.130044
ENSMUSG00000031273	Col4a6	collagen, type IV, alpha 6 (Col4a6), mRNA	type IV	0.00000051	3.663765
ENSMUSG00000026837	Col5a1	collagen, type V, alpha 1 (Col5a1), mRNA	type V	0.000273973	1.73128
ENSMUSG00000026042	Col5a2	collagen, type V, alpha 2 (Col5a2), mRNA	type V	0.442105017	0.828681
ENSMUSG00000004098	Col5a3	collagen, type V, alpha 3 (Col5a3), mRNA	type V	0.989220301	-0.32152
ENSMUSG00000001119	Col6a1	collagen, type VI, alpha 1 (Col6a1), mRNA	type VI	0.000000256	1.907141
ENSMUSG00000020241	Col6a2	collagen, type VI, alpha 2 (Col6a2), mRNA	type VI	0.0000231	1.60699
ENSMUSG00000048126	Col6a3	collagen, type VI, alpha 3 (Col6a3), mRNA	type VI	0.003193055	1.289803
ENSMUSG00000032572	Col6a4	collagen, type VI, alpha 4 (Col6a4), mRNA	type VI	low expression	
ENSMUSG00000091345	Col6a5	collagen, type VI, alpha 5 (Col6a5), mRNA	type VI	0.989220301	0.322323
ENSMUSG00000043719	Col6a6	collagen, type VI, alpha 6 (Col6a6), mRNA	type VI	low expression	
ENSMUSG00000025650	Col7a1	collagen, type VII, alpha 1 (Col7a1), mRNA	type VII	0.989220301	-0.26505
ENSMUSG00000068196	Col8a1	collagen, type VIII, alpha 1 (Col8a1), mRNA	type VIII	low expression	
ENSMUSG00000056174	Col8a2	collagen, type VIII, alpha 2 (Col8a2), mRNA	type VIII	0.446143852	0.970351
ENSMUSG00000026147	Col9a1	collagen, type IX, alpha 1 (Col9a1), mRNA	type IX	low expression	
ENSMUSG00000028626	Col9a2	collagen, type IX, alpha 2 (Col9a2), mRNA	type IX	0.00000051	3.616545
ENSMUSG00000027570	Col9a3	collagen, type IX, alpha 3 (Col9a3), mRNA	type IX	0.000000014	3.965448
ENSMUSG00000039462	Col10a1	collagen, type X, alpha 1 (Col10a1), mRNA	type X	low expression	
ENSMUSG00000027966	Col11a1	collagen, type XI, alpha 1 (Col11a1), mRNA	type XI	1.04E-15	4.296395
ENSMUSG00000024330	Col11a2	collagen, type XI, alpha 2 (Col11a2), mRNA	type XI	0.00000051	2.899853
ENSMUSG00000032332	Col12a1	collagen, type XII, alpha 1 (Col12a1), mRNA	type XII	0.056573025	1.102192
ENSMUSG00000058806	Col13a1	collagen, type XIII, alpha 1 (Col13a1), mRNA	type XIII	0.996574685	0.015684
ENSMUSG00000022371	Col14a1	collagen, type XIV, alpha 1 (Col14a1), mRNA	type XIV	0.000000146	2.296451
ENSMUSG00000028339	Col15a1	collagen, type XV, alpha 1 (Col15a1), mRNA	type XV	0.989220301	-0.18883
ENSMUSG00000040690	Col16a1	collagen, type XVI, alpha 1 (Col16a1), mRNA	type XVI	0.000372668	1.634199
ENSMUSG00000025064	Col17a1	collagen, type XVII, alpha 1 (Col17a1), mRNA	type XVII	low expression	
ENSMUSG00000001435	Col18a1	collagen, type XVIII, alpha 1 (Col18a1), mRNA	type XVIII	0.00000182	1.561112
ENSMUSG00000026141	Col19a1	collagen, type XIX, alpha 1 (Col19a1), mRNA	type XIX	low expression	
ENSMUSG00000016356	Col20a1	collagen, type XX, alpha1 (Col20a1), mRNA	type XX	0.99369326	-0.12657
ENSMUSG00000079022	Col22a1	collagen, type XXII, alpha 1 (Col22a1), mRNA	type XXII	0.989220301	-0.25757
ENSMUSG00000063564	Col23a1	collagen, type XXIII, alpha 1 (Col23a1), mRNA	type XXIII	0.995426174	0.053846
ENSMUSG00000028197	Col24a1	collagen, type XXIV, alpha 1 (Col24a1), mRNA	type XXIV	low expression	
ENSMUSG00000058897	Col25a1	collagen, type XXV, alpha 1 (Col25a1), mRNA	type XXV	low expression	
ENSMUSG00000004415	Col26a1	collagen, type XXVI, alpha 1 (Col26a1), mRNA	type XXVI	low expression	
ENSMUSG00000045672	Col27a1	collagen, type XXVII, alpha 1 (Col27a1), mRNA	type XXVII	0.010482257	1.417136
ENSMUSG00000068794	Col28a1	collagen, type XXVIII, alpha 1 (Col28a1), mRNA	type XXVIII	low expression	

B

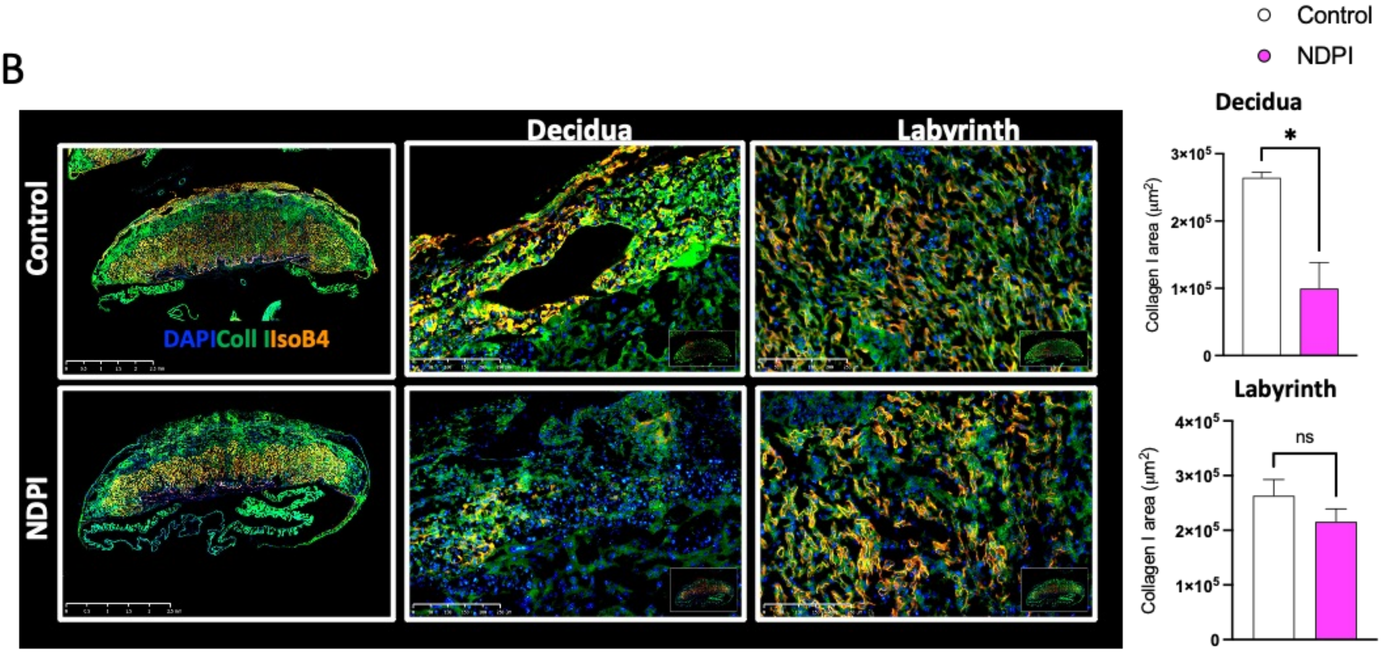

Supplementary Figure 2

Experiment as outlined in S1-A. Mice were sacrificed at E14.5 of pregnancy and placentas harvested and processed for RNA sequencing.

(A) Gene expression of the 44 collagen genes of the mouse genome NDPI vs control.

(B) Immunofluorescent staining of placentas for Collagen 1 (green) isolectin b4 (orange) and cell nuclei with DAPI (blue). Expression is shown in the whole placenta (left) decidual layer (middle) and labyrinth layer (right). Graphs showing quantification of the total area of Col I expression in the decidua and labyrinth. Scale bar = 2.5mm

Each symbol represents an individual mouse and statistical significance was tested by Welch's t-test (Decidua) or Student's unpaired t-test (Labyrinth). ns = not significant, *$p \leq 0.05$. Data are mean ± SEM.

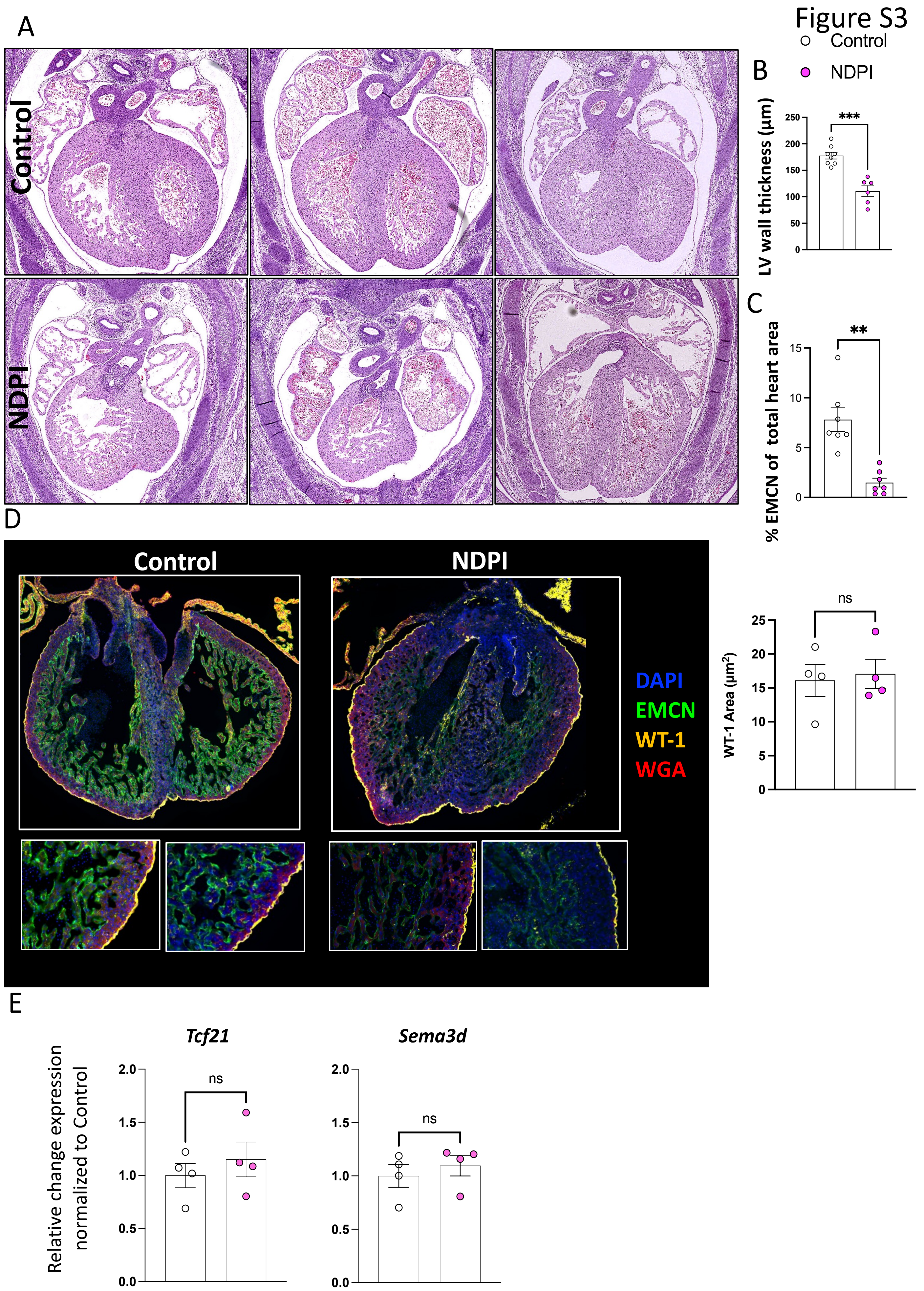
Figure S3
A
Control
NDPI
Control
NDPI
B
LV wall thickness (µm)
250
200
150
100
50
0
***
C
% EMCN of total heart area
15
10
5
0
**
D
Control
NDPI
DAPI
EMCN
WT-1
WGA
WT-1 Area (µm²)
25
20
15
10
5
0
ns
E
Relative change expression normalized to Control
Tcf21
Sema3d
2.0
1.5
1.0
0.5
0.0
ns
ns

Supplementary Figure 3

(A) Representative H&E images of E14.5 embryonic hearts from 3 independent control and 3 independent NDPI pregnancies taken at X40 magnification

(B) Left ventricular (LV) wall thickness measurement of E14.5 embryonic hearts from control and NDPI pregnancies

(C) Quantification of endomucin staining in E14.5 embryonic hearts shown in Figure 3A, from both groups, expressed as percentage of total heart area (bottom panel)

(D) Staining for epicardial-specific Wilm's tumour-1 (WT-1) in E14.5 embryonic hearts from control and NDPI pregnancies

(E) q-PCR analyses of genes expression of epicardial-specific genes, *Tcf21* and *Sema3d*

Each symbol represents an individual mouse and statistical significance was tested by Welch's t-test (B) or Student's t-test (E). ns = not significant, ***p≤0.0001. Data are mean ± SEM.

Figure S4

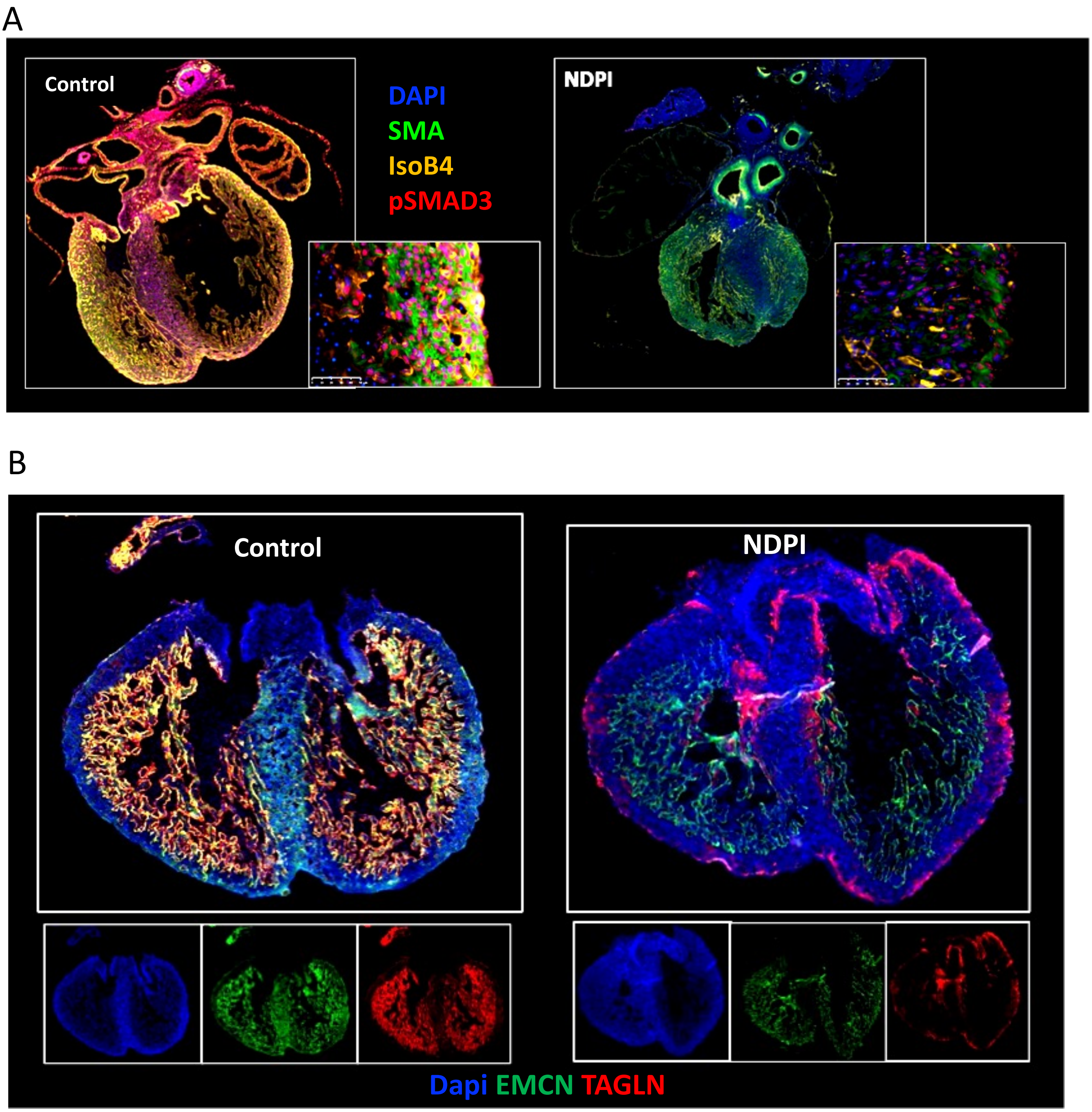
A
Control
DAPI
SMA
IsoB4
pSMAD3
NDPI
B
Control
NDPI
Dapi EMCN TAGLN

Supplementary Figure 4

(A) Immunofluorescence staining of E14.5 embryonic hearts from control and NDPI pregnancies. Hearts were stained for Dapi (blue), smooth muscle actin (SMA; green), isolectinB4 for vessels (orange) and phosphor(p)SMAD3 (Red) Representative images shown. Scale bar =0.5 mm

(B) Immunofluorescence staining of E14.5 embryonic hearts from control and NDPI pregnancies. Hearts were stained for Dapi (blue), smooth muscle actin (SMA; green), and transgelin (Tagln; Red). Representative images shown. Scale bar = 0.5mm

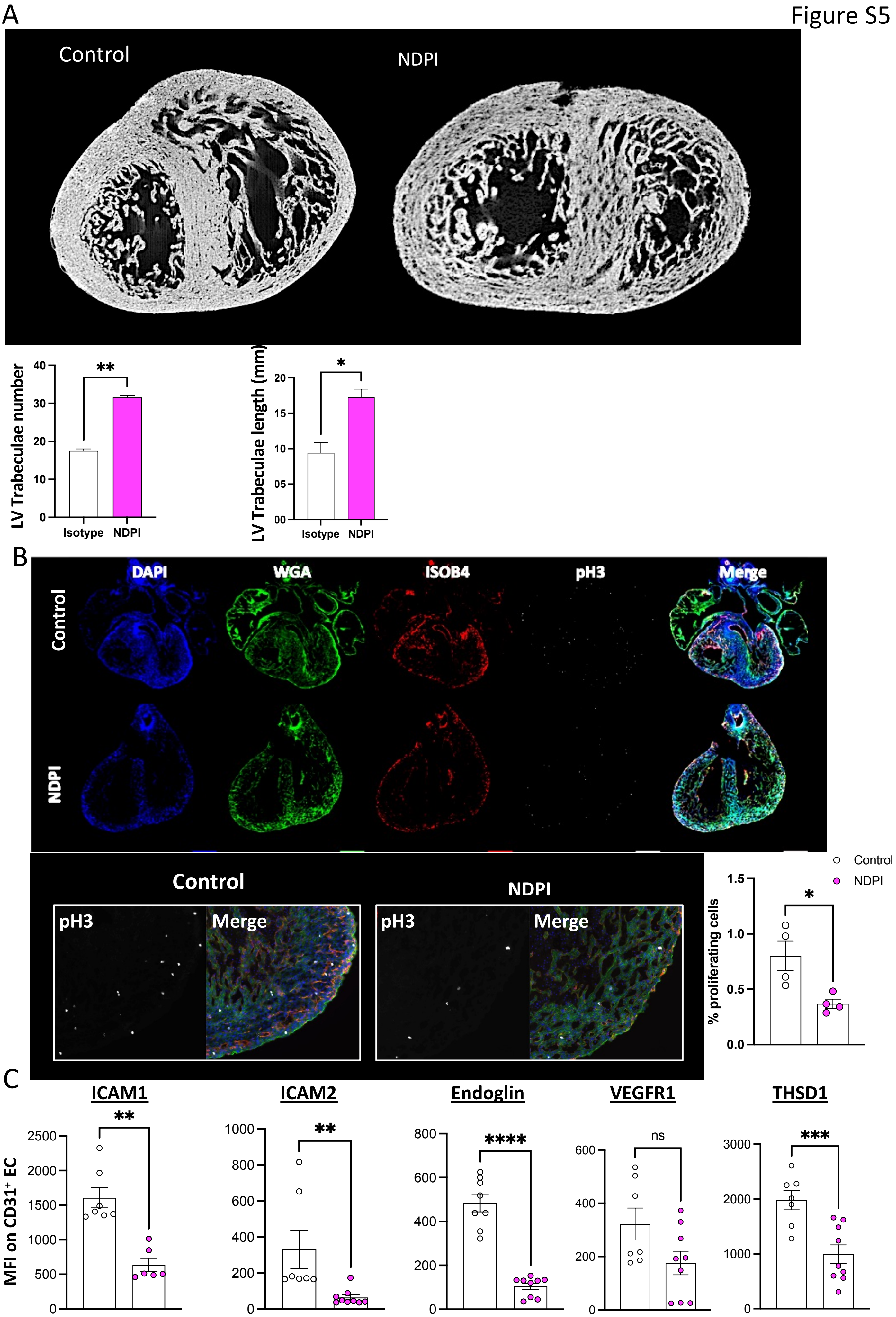

Figure S5
A
Control
NDPI
LV Trabeculae number
**
Isotype
NDPI
LV Trabeculae length (mm)
*
Isotype
NDPI
B
DAPI
WGA
ISOB4
pH3
Merge
Control
NDPI
Control
NDPI
pH3
Merge
pH3
Merge
Control
NDPI
% proliferating cells
*
C
ICAM1
ICAM2
Endoglin
VEGFR1
THSD1
MFI on CD31+ EC
**
**
****
ns
***

Supplementary Figure 5

(A) Quantification of number and length of left ventricular (LV) trabeculae analsyed from HREM images. Scale bar = 0.5mm

(B) Analyses of proportion of proliferating cells within E14.5 embryonic hearts using phoso-histone H3 (pH3). Scale bar = 0.5mm

(C) CD31+ endothelial cells from hearts of E14.5 embryos were analyzed for the expression of ICAM1, ICAM2, Endoglin, VEGFR1 and THSD1. Quantification showing the median fluorescent intensity (MFI) of the corresponding surface marker.

Each symbol represents an individual embryonic hearts from distinct pregnancies and statistical significance was tested by unpaired Student's t-test . ns = not significant, *p≤0.05, ** p≤0.01, ***p≤0.001 **** p≤0.0001. In all cases, data are mean ± SEM.

Figure S6

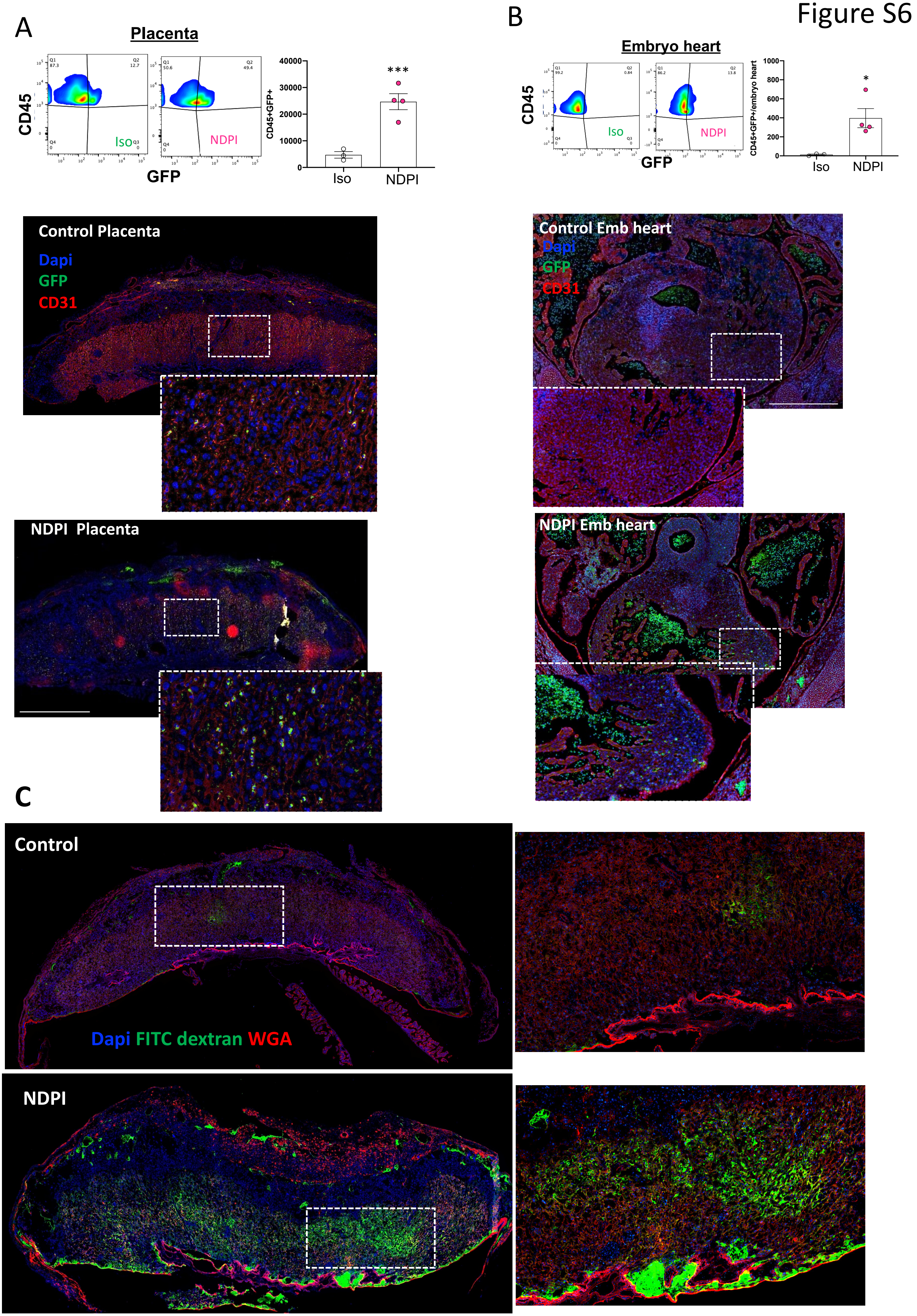

A
Placenta
CD45
GFP
Iso
NDPI
CD45+GFP+
***
B
Embryo heart
CD45
GFP
Iso
NDPI
CD45+GFP+/embryo heart
*
Control Placenta
Dapi
GFP
CD31
NDPI Placenta
Control Emb heart
Dapi
GFP
CD31
NDPI Emb heart
C
Control
Dapi FITC dextran WGA
NDPI

Supplementary Figure 6

(A) Flow cytometric and immunofluorescent analyses of placentas from *In vivo* transfer of GFP^{+} leukocytes in Control and NDPI pregnancies at E14.5. Images taken at x20 magnification and x200 magnification (dotted lined box)

(B) Flow cytometric and immunofluorescent analyses of embryonic hearts from *In vivo* transfer of GFP^{+} leukocytes in isotype and NDPI pregnancies at E14.5. Images taken at x20 magnification and x200 magnification (dotted lined box)

(C) Immunofluoresnce images of placentas from *In vivo* FITC-dextran infusion for 1 hour prior to sacrifice from isotype and NDPI pregnancies at E14.5. Images taken at x20 magnification and x200 magnification (dotted lined box).

Scale bar = 0.5mm Each symbol represents an individual mouse and statistical significance was tested by unpaired Student's t-test (placenta) or Welch's t-test (embryo heart). ns = not significant, *$p \leq 0.05$, ** $p \leq 0.01$, ***$p \leq 0.001$ **** $p \leq 0.0001$. In all cases, data are mean ± SEM.

Figure S7

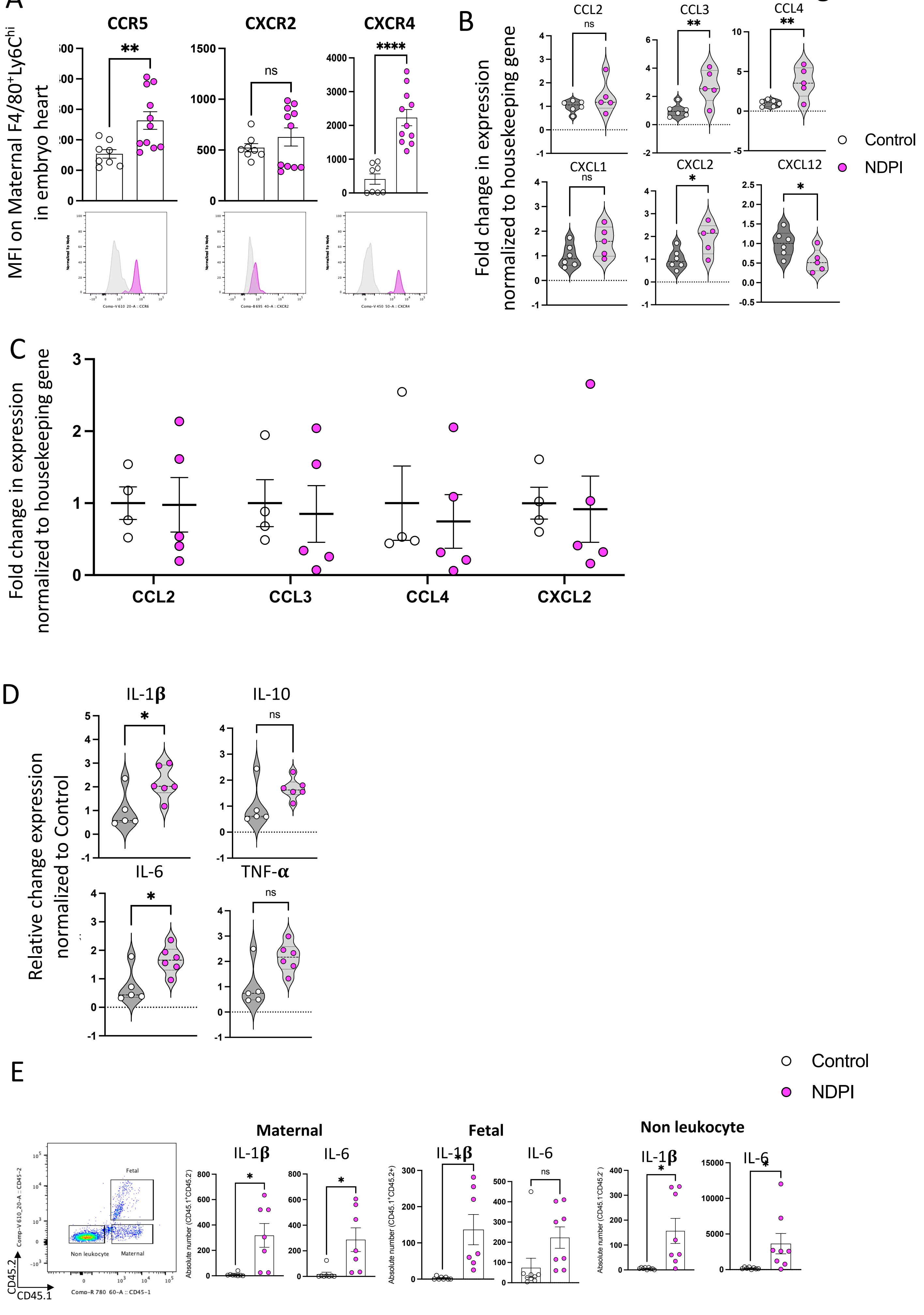

A
CCR5
CXCR2
CXCR4
MFI on Maternal F4/80+Ly6Chi in embryo heart
**
ns
****
B
CCL2
CCL3
CCL4
CXCL1
CXCL2
CXCL12
ns
**
*
Fold change in expression normalized to housekeeping gene
Control
NDPI
C
Fold change in expression normalized to housekeeping gene
CCL2
CCL3
CCL4
CXCL2
D
IL-1β
IL-10
IL-6
TNF-α
Relative change expression normalized to Control
E
Control
NDPI
Maternal
Fetal
Non leukocyte
IL-1β
IL-6
Absolute number (CD45.1+CD45.2-)
Absolute number (CD45.1+CD45.2+)
Absolute number (CD45.1-CD45.2-)
Fetal
Non leukocyte
Maternal
CD45.2
CD45.1

Supplementary Figure 7

(A) Median fluorescent intensity of MHCII, CCR2, CXCR2, CCR5 and CCR6 on maternal F4/80+Ly6Chi cells found in embryo hearts. Lower panel, representative histograms.

(B) Violin plot showing gene expression of chemokines in embryo hearts from control and NDPI pregnancies normalized to control

(C) Graph showing gene expression of CCL2, CCL3, CCL4 and CXCL2 in embryo livers from neutrophil depleted pregnancies normalised to isotype control.

(D) Violin plot showing gene expression of cytokines in embryo hearts from control and NDPI pregnancies normalized to control

(E) Representative FACs plot showing non leukocytes, maternal leukocytes ($CD45.1^{+}$ $CD45.2^{-}$) and fetal cells ($CD45.1^{+}CD45.2^{+}$) in embryo heart. Graphs show quantification of number of cells in maternal or fetal origin leukocytes or from non-leukocytes expressing Il-1β and IL-6.

Each symbol represents an individual mouse and statistical significance was tested by unpaired Student's t-test or Welch's t-test (CCR5, CXCR4 in A; CCL4 in B; and Maternal IL-1b and IL-6, Fetal IL-1b and non-leukocyte IL-1b and IL-6 in E). ns = not significant, *p≤0.05, ** p≤0.01, ***p≤0.001 **** p≤0.0001. In all cases, data are mean ± SEM.

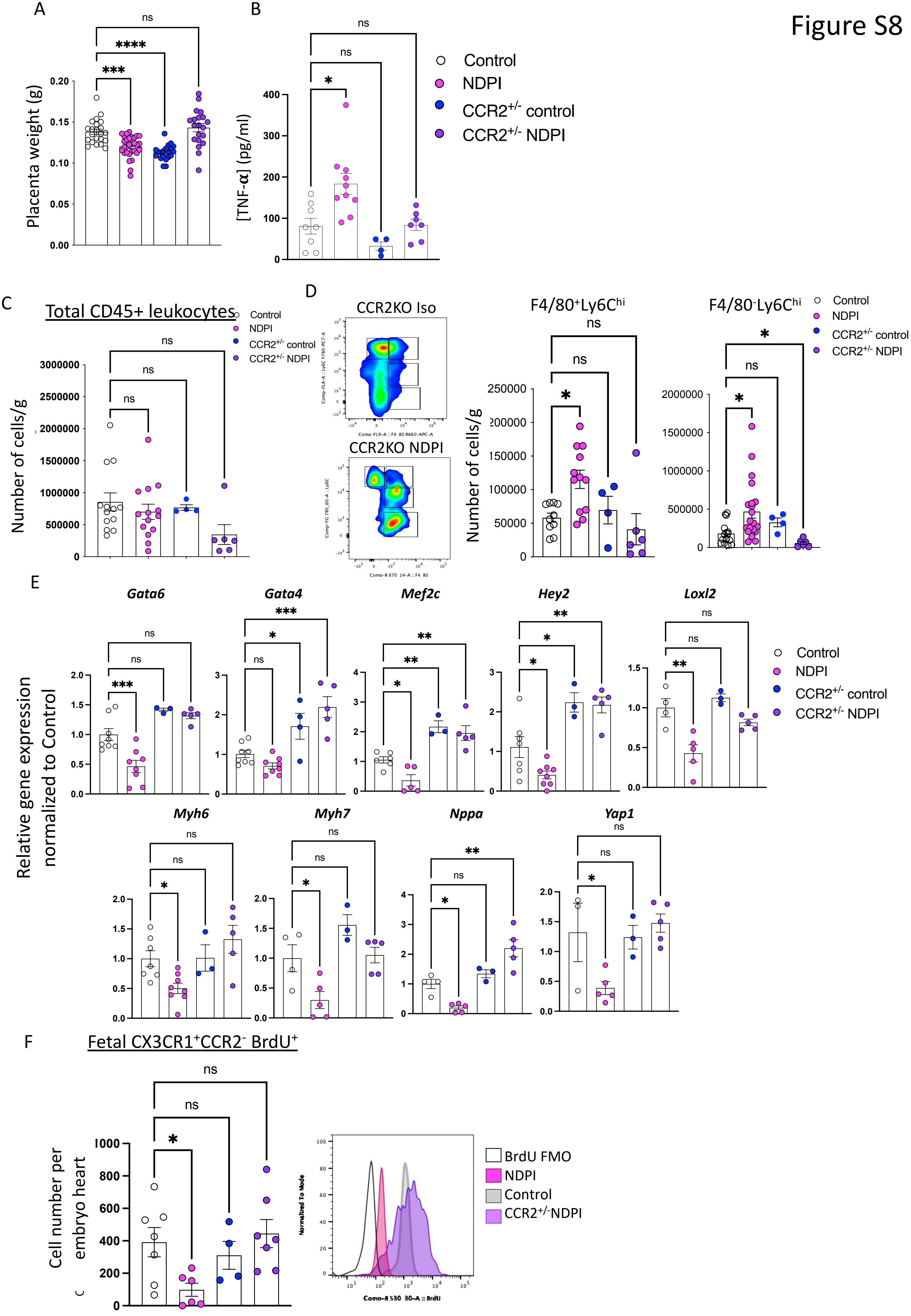
A
Placenta weight (g)
ns
****
***
B
[TNF-α] (pg/ml)
ns
ns
*
Control
NDPI
CCR2+/- control
CCR2+/- NDPI
C
Total CD45+ leukocytes
Number of cells/g
D
CCR2KO Iso
CCR2KO NDPI
F4/80+Ly6Chi
F4/80-Ly6Chi
E
Gata6
Gata4
Mef2c
Hey2
Loxl2
Myh6
Myh7
Nppa
Yap1
Relative gene expression normalized to Control
F
Fetal CX3CR1+CCR2- BrdU+
Cell number per embryo heart
BrdU FMO
NDPI
Control
CCR2+/-NDPI

Figure S8

Supplementary Figure 8

(A) Comparison of E14.5 placenta weights from Control, NDPI, $CCR2^{+/-}$control and $CCR2^{+/-}$ NDPI pregnancies

(B) Comparison of E14.5 placenta TNF-α production, as measured by standard ELISA, from Control, NDPI, $CCR2^{+/-}$control and $CCR2^{+/-}$ NDPI pregnancies

(C) Quantification of total CD45+ leukocytes by flow cytometry in E14.5 placentas from Control, NDPI, $CCR2^{+/-}$control and $CCR2^{+/-}$ NDPI pregnancies. Numbers are quantified as per gram of placenta tissue

(D) Representative FACS plots of F4/80 and Ly6c populations in $CCR2^{+/-}$control and $CCR2^{+/-}$NDPI placentasLoxl2 (compare with isotype and NDPI plots shown in Figure 1H); Comparison of $F4/80^{+}Ly6C^{hi}$ macrophage and $F4/80^{-}Ly6C^{hi}$ monocyte populations within the placenta from NDPI, $CCR2^{+/-}$control and $CCR2^{+/-}$NDPI pregnancies with Control. Numbers expressed as per gram of placenta tissue

(E) RT (q) PCR analyses of key heart development genes from E14.5 embryo hearts of Control, NDPI, $CCR2^{+/-}$control and $CCR2^{+/-}$NDPI pregnancies. Data compare gene transcription changes from embryonic hearts of NDPI, $CCR2^{+/-}$control and $CCR2^{-/-}$ NDPI pregnancies to Control.

(F) Flow cytometric analyses of resident fetal CX3CR1+CCR2- macrophage proliferation, measured by *in vivo* BrdU incorporation. Data compare proliferation from embryonic hearts of NDPI, $CCR2^{+/-}$control and $CCR2^{+/-}$ NDPI pregnancies compared to Control.

Each symbol represents an individual mouse and statistical significance was tested one-way ANOVA or Brown-Forsythe test (A, B and D), with Dunnett's multiple comparison test. ns = not significant, *p≤0.05, ** p≤0.01, ***p≤0.001 **** p≤0.0001. In all cases, data are mean ± SEM.

Figure S9

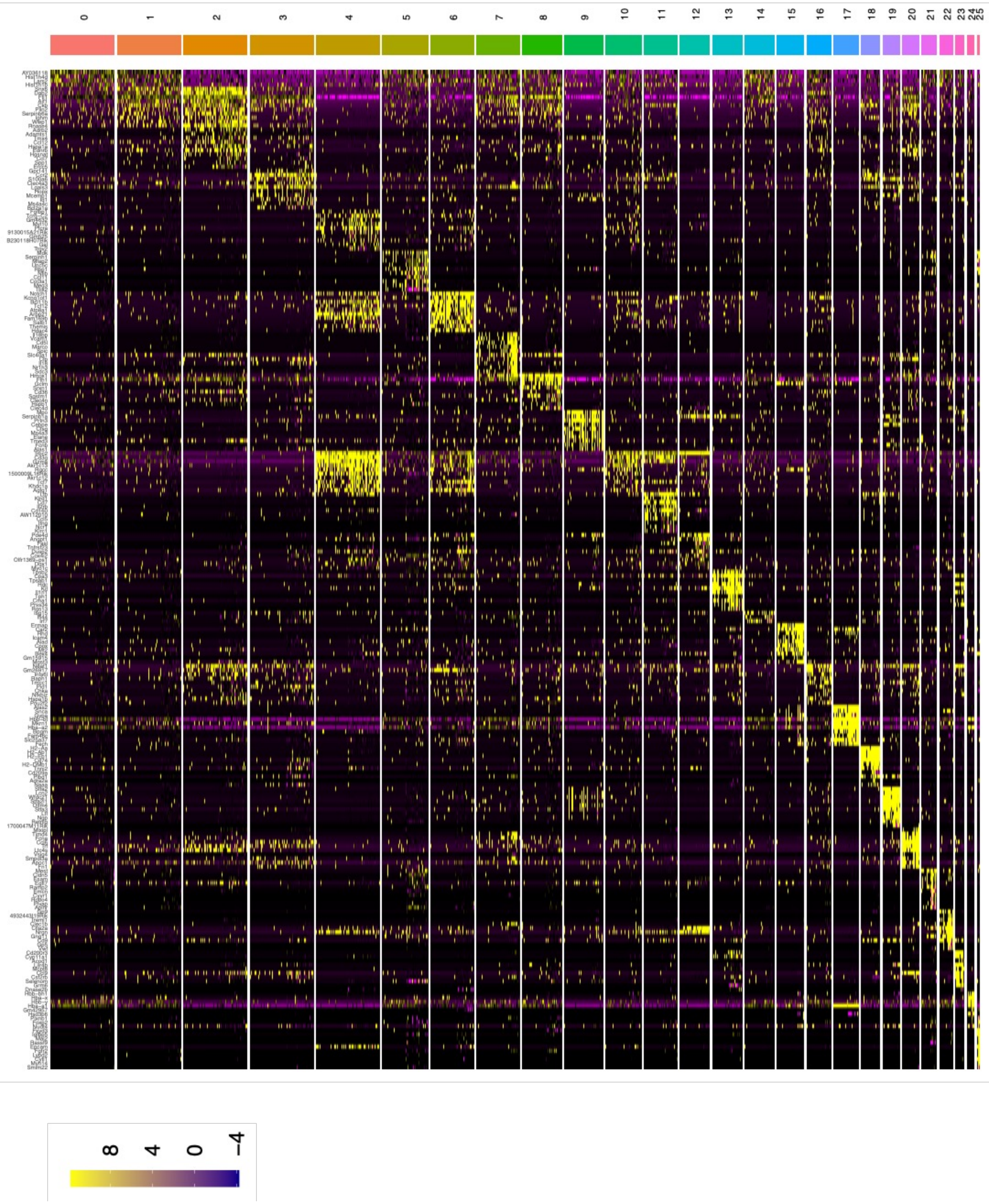

Supplementary Figure 9

Neutrophils were depleted at day 4.5 and 7.5 of pregnancy using αLy6G. Mice were sacrificed at E14.5 of pregnancy and hearts dissected from harvested embryos from control or neutrophil depleted placental inflammation (NDPI) pregnancies. $CD45^{+}$ cells were isolated from heart single cell suspensions using CD45 PE and anti-PE microbeads. Single cell sequencing was performed on the isolated cells.

Heat map of single-cell gene expression data across all clusters

Figure S10

Cluster number	Cell type	Top genes
0	Resident Fetal Macrophages	See figure 5 C
1	Macrophage 1	AY036118 Hist1h4d Lars2 Hist1h1b Snx6 Dab2 Ftl1 Aif1 Ckb Plin2 Serpinb6a Ighm
2	Macrophage 2	Hist1h1b Snx6 Dab2 Ftl1 Aif1 Ckb Plin2 Serpinb6a Ighm Wwp1 Rnase4 Adrb2 Adamts1 Tmx4 Ccl12 Hspa1a Ednrb Hgsnat Ccl7
3	Maternal leukocytes	See figure 5 C
4	T cells	Fgfbp1 Tcrg−C2 Gm4632 Myl10 Ptcra 9130015A21Rik Gm525 Notch1 Kcnq1ot1 Bcl11b Tcf12 Atp8a1 Arpp21 Fam169b Cd3g Gzma Akr1c13
5	Endothelial 1	Mfap2 Unc5c Rbp1 Nfib Ccl11 Col3a1 Meg3 Tbx2 Notch1
6	NKT cells	Notch1 Kcnq1ot1 Bcl11b Tcf12 Atp8a1 Arpp21 Fam169b Satb1 Themis Cd3g Gzma Akr1c13 Rgcc 1500009L16Rik Akr1c12 Tcf7
7	Unknown	Il18 Nr1h3 Sdc3 Hmox1 Fth1 Gclm
8	Unknown	Hmox1 Fth1 Gclm Srxn1 Cd36 Sqstm1 Clec4n Hspb1 Clec4d
9	Neutrophil 1	Mpo Serpinb1a Prtn3 Cebpe Ctsg Ms4a3 Elane Tmed3 Fcnb Alas1
10	T cell 2	Alas1 Trbc2 Cd3g Gzma Akr1c13 Rgcc 1500009L16Rik Akr1c12 Tcf7 Khdc1a Agfg1
11	Bcell 2	Klrd1 Cd7 Il2rb Cd160 AW112010 Ccl5 Ifng Ncr1 Klrc1
12	B cell 1	Angpt1 Fasl Tnfrsf23 Dusp2 Cox4i2 Olfr1369−ps1 Dqx1 Myo1c Clec1b Ctla2a Nrgn Gng11 Cd9
13	Mast cell 1	Tpsb2 Cpa3 Tpsab1 Hdc Kit Il1rl1 Tph1 Cma1 Prss34 Rgs13
14	Unknown	Isg15 Ifi44 Irf7
15	Macrophage 3	Irf7 Ermap Car2 Rhd Icam4 Alad Cpox Mt2 Bex4
16	Unknown	Gm26917 Ints6l Raph1 Tmcc1 Pid1 Chka Nfkbi
17	Myeloid origin	Snca Gypa Hbb−bt Mkrn1 Hba−a2 Bpgm Fam46c Slc25a37
18	Dendritic Cells	H2−Aa H2−Ab1 H2−Eb1 Cd74 H2−DMb1 Tnni2 Cd209a Plbd1 Adra2a
19	Neutrophil 2	Stfa2 Lcn2 Wfdc21 Stfa2l1 Olfm4 Stfa3 Ltf Ngp Retnlg
20	Monocyte/macrophage	Fcna Ccl6 Trf Ltc4s Vsig4 Smpdl3a Apoc1 Fn1
21	Endothelial 2	Cldn5 Esam Egfl7 Ramp2 Emcn Cyyr1 Robo4 Plvap Aplnr
22	Endothelial-like 1	Clec1b Ctla2a Nrgn Gng11 Cd9 Gp5 Vwf
23	Mast cell 2	Cd200r3 Cyp11a1 Acod1 Lilr4b Mcpt8 Ccl9 Csf2rb Selenom Grm6
24	Unknown	Hbb−bh1 Hba−x Hbb−y Hba−a1
25	Endothelial-like 2	Mal2 Rassf9 Epcam Fgfr2 Lypd2 Cytl1 Myh14 Smim22

Supplementary Figure 10

Data in the table indicate top genes expressed in each cluster of the single-cell analyses, as described in Figure S9. These genes were used to identify the cell types for each of the clusters

Figure S11

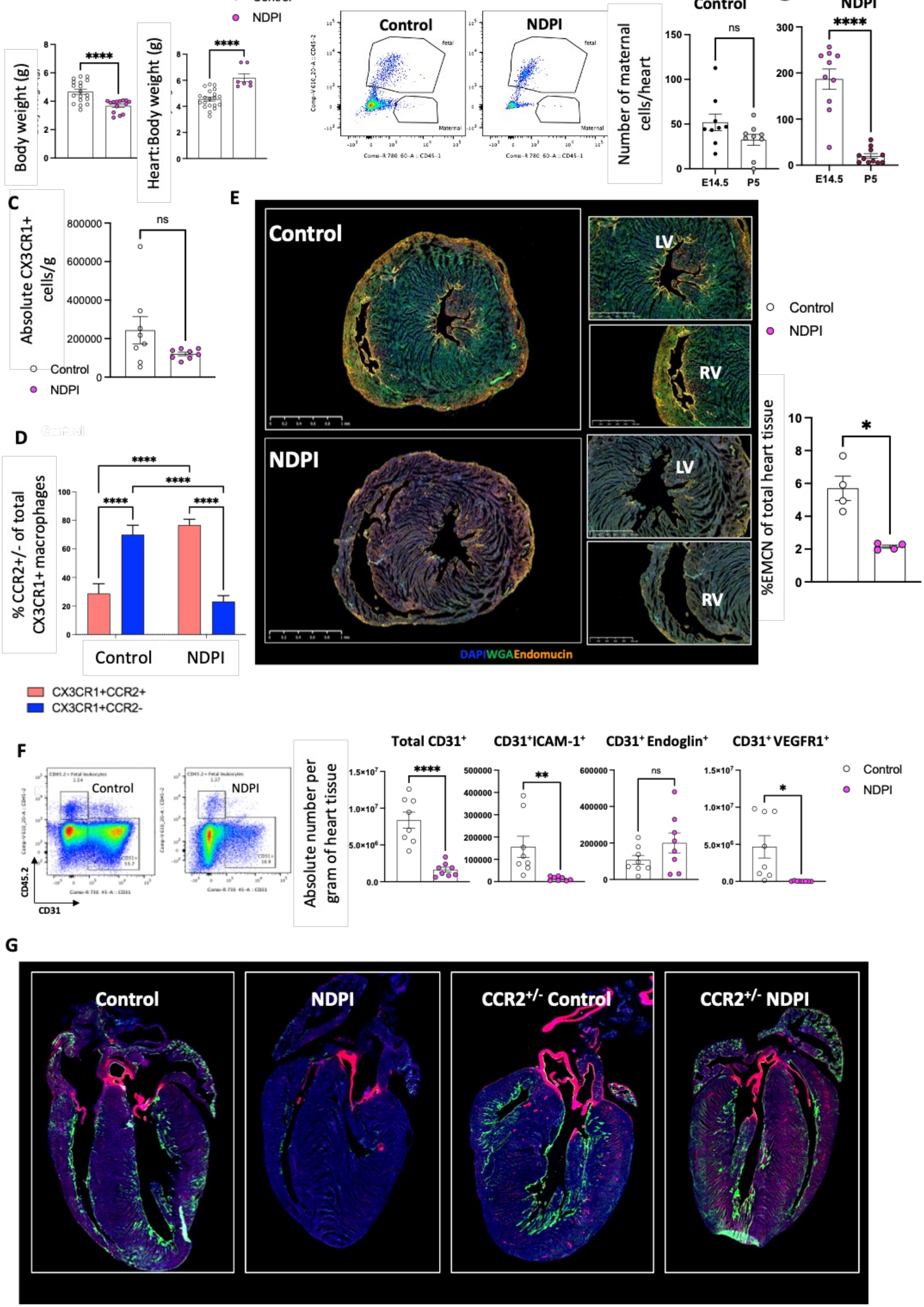
A
Postnatal day 5
Control
NDPI
Body weight (g)
Heart:Body weight (g)
****
B
Control
NDPI
fetal
Maternal
Number of maternal cells/heart
ns
****
E14.5
P5
C
Absolute CX3CR1+ cells/g
ns
D
% CCR2+/- of total CX3CR1+ macrophages
CX3CR1+CCR2+
CX3CR1+CCR2-
E
LV
RV
DAPI WGA Endomucin
%EMCN of total heart tissue
*
F
Total CD31+
CD31+ICAM-1+
CD31+ Endoglin+
CD31+ VEGFR1+
Absolute number per gram of heart tissue
CD45.2
CD31
**
G
Control
NDPI
CCR2+/- Control
CCR2+/- NDPI

Supplementary Figure 11

(A) Neutrophils were depleted at day 4.5 and 7.5 of pregnancy using αLy6G. Offspring of these dams were sacrificed at post-natal day 5 (P5) or 28 as indicated and heart structure and immune composition assessed. Offspring from Control (white) and neutrophil depleted (NDPI) (pink). P5 offspring body weights in grams and heart: body weight ratio.

(B) Flow cytometry plots of leukocytes in postnatal day 5 hearts from control of NDPI pregnancies; $CD45.1^{+}$ $CD45.2^{-}$ cells are of maternal origin and $CD45.1^{+}CD45.2^{+}$ cells are of fetal origin. Graph comparing number of maternal cells in offspring hearts at E14.5 and P5.

(C) Flow cytometric analyses of CX3CR1+ macrophages of fetal origin in P5 offspring hearts from isotype and NDPI pregnancies expressed as per gram of heart tissue. Representative flow cytometry plots shown.

(D) Proportion of $CCR2^{+}$ and $CCR2^{-}$ cells within the total population of CX3CR1+ macrophages in P5 heart

(E) Representative immunofluorescent images showing the expression of endomucin and wheat germ agglutinin in transverse sections of hearts from P5 offspring from control or NDPI x20 magnification. Scale bar 1mm. Magnified (x200) images showing endomucin staining of the ventricles (LV -left; RV, right). Graph showing the proportion of total heart area expressing endomucin. Hearts show Dapi (blue) wheat germ agglutinin (green) and endomucin (orange). Scale bar = 1mm

(F) Representative FACS plots showing heart $CD31^{+}$ populations from P5 offspring from dams treated with either isotype control or αLy6G neutralizing antibody. Graphs showing the number of CD31+ cells per gram of heart tissue and the number of these $CD31^{+}$ cells expressing ICAM-1, Endoglin and VEGFR1.

(G) Longitudinal sections of offspring P5 hearts from isotype, NDPI, $CCR2^{+/-}$control and $CCR2^{+/-}$ NDPI pregnancies. Hearts show Dapi (blue), wheat germ agglutinin (WGA; green) and endomucin (orange). X20 magnification

Each symbol represents an individual mouse and statistical significance was tested Student's unpaired t-test (A and C), Brown-Forsythe test with Dunnett's multiple comparison test (D). Welch's t-test (B, E and F -CD31), Mann-Whitney test (F- VEGFR1). ns = not significant, *p≤0.05, ** p≤0.01, ***p≤0.001 **** p≤0.0001. In all cases, data are mean ± SEM.

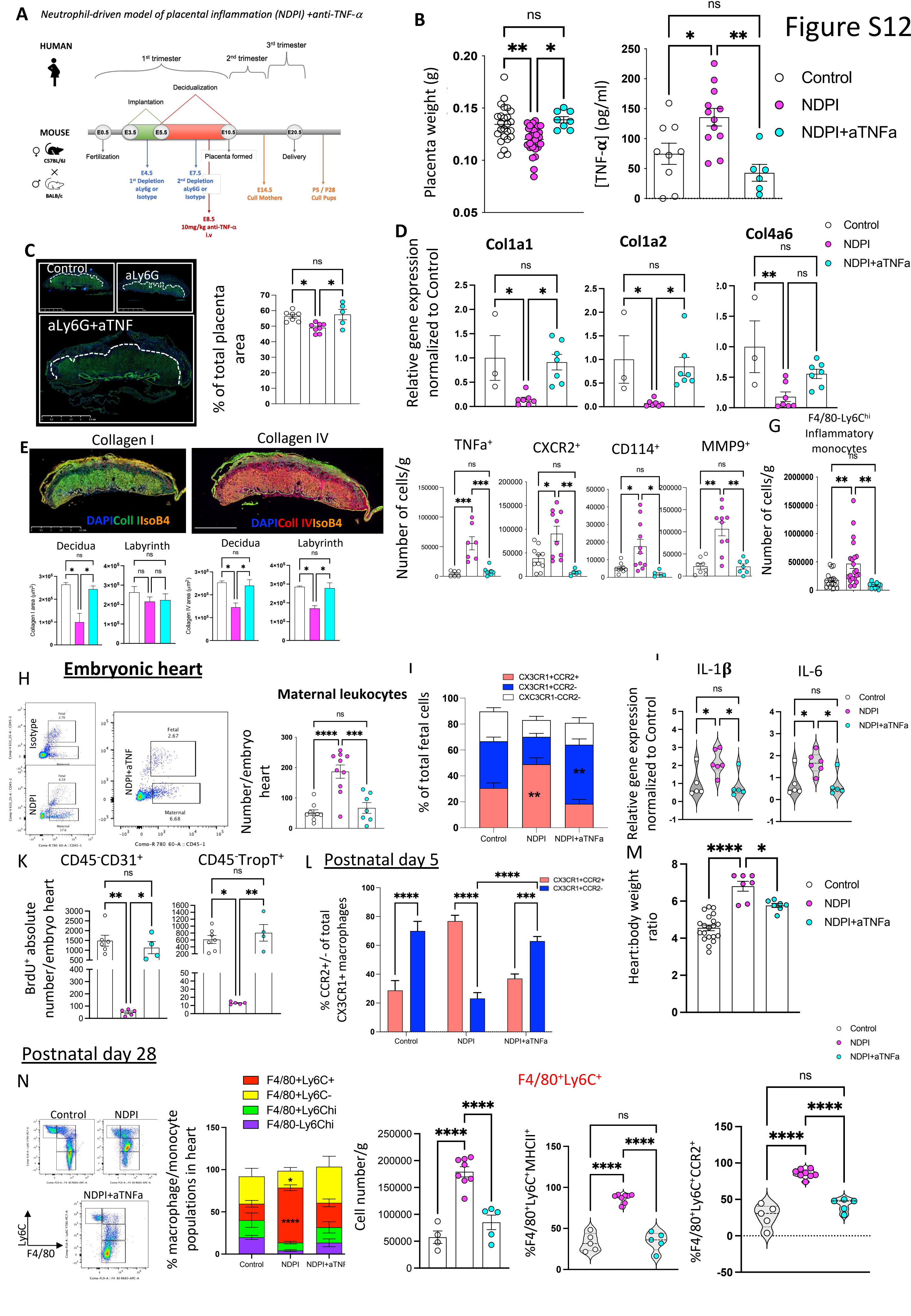

Figure S12
A
Neutrophil-driven model of placental inflammation (NDPI) +anti-TNF-α
HUMAN
1st trimester
2nd trimester
3rd trimester
Decidualization
Implantation
MOUSE
C57BL/6J
BALB/c
E0.5
E3.5
E5.5
E10.5
E20.5
Fertilization
Placenta formed
Delivery
E4.5
1st Depletion
aLy6g or Isotype
E7.5
2nd Depletion
aLy6G or Isotype
E8.5
10mg/kg anti-TNF-α i.v
E14.5
Cull Mothers
P5 / P28
Cull Pups
B
Placenta weight (g)
[TNF-α] (pg/ml)
Control
NDPI
NDPI+aTNFa
C
Control
aLy6G
aLy6G+aTNF
% of total placenta area
D
Relative gene expression normalized to Control
Col1a1
Col1a2
Col4a6
E
Collagen I
Collagen IV
DAPI Coll I IsoB4
DAPI Coll IV IsoB4
Decidua
Labyrinth
Collagen I area (μm²)
Collagen IV area (μm²)
Number of cells/g
TNFa+
CXCR2+
CD114+
MMP9+
G
F4/80-Ly6Chi Inflammatory monocytes
H
Embryonic heart
Isotype
NDPI
NDPI+aTNF
Fetal
Maternal
Maternal leukocytes
Number/embryo heart
I
CX3CR1+CCR2+
CX3CR1+CCR2-
CX3C3R1-CCR2-
% of total fetal cells
IL-1β
IL-6
Relative gene expression normalized to Control
K
CD45-CD31+
CD45-TropT+
BrdU+ absolute number/embryo heart
L
Postnatal day 5
% CCR2+/- of total CX3CR1+ macrophages
M
Heart:body weight ratio
Postnatal day 28
N
F4/80+Ly6C+
F4/80+Ly6C-
F4/80+Ly6Chi
F4/80-Ly6Chi
Ly6C
F4/80
% macrophage/monocyte populations in heart
Cell number/g
%F4/80+Ly6C+MHCII+
%F4/80+Ly6C+CCR2+

Supplementary Figure 12

(A) Dampening of exaggerated placental inflammation - schematic for experimental design.

(B) Weights of E14.5 placentas ELISA showing the concentration of TNF-α in placenta digest supernatants. Control treated (white), neutrophil depleted (NDPI) (pink), neutrophil depleted TNFα neutralised (NDPI + aTNFα) (blue).

(C) Immunofluorescent staining of placentas for wheat germ agglutinin (WGA) (green) to show gross structure and cell nuclei with DAPI (blue). White dotted line indicates the separation of the labyrinthine zone/trophoblast area and the outer junctional zone. Graph showing the proportion of the total area of the placentas occupied by the trophoblast zone. Scale bar =2.5mm

(D) RT-PCR showing the gene expression of Col1a1, col1a2 and col4a6 in placentas from control and NDPI and NDPI + aTNFa pregnancies normalised to control.

(E) Immunofluorescent staining of placentas for Collagen I (green) isolectin b4 (orange) and cell nuclei with DAPI (blue) (left image) or Collagen IV isolectin b4 (orange) and cell nuclei with DAPI (blue) (right image). Graphs showing quantification of the total area of Col I or Col IV expression in the decidua and labyrinth. Scale bar = 2.5mm

(F) Absolute number of neutrophils expressing TNFα, CXCR2, CD114 and MMP9 from placentas analyzed by flow cytometry expressed as absolute cell number per gram of tissue.

(G) Inflammatory monocytes from placentas analyzed by flow cytometry expressed as absolute cell number per gram of tissue.

(H) Representative FACS plots showing fetal and maternal leukocytes in embryo heart and graph showing quantification of number of maternal leukocytes per embryo heart.

(I) Proportion of total cells in embryo heart expressing CX3CR1+CCR2+, CX3CR1+CCR2- or CX3CR1-CCR2-.

(J) Violin plot showing gene expression of IL-1**β** and IL-6 in embryo hearts from control and NDPI pregnancies normalized to control.

(K) Flow cytometric analyses of endothelial (CD45-CD31+) and cardiomyocyte (CD45-Troponin-T+) proliferation, as measured by in vivo BrdU incorporation from E14.5 embryonic hearts of isotype, NDPI and NDPI+aTNFa pregnancies

(L) Proportion of CCR2+ and CCR2- CX3CR1+ macrophages from P5 offspring hearts from isotype, NDPI and NDPIaTNFα pregnancies

(M) Comparison of heart: body weight rations from P5 offspring of isotype, NDPI and NDPIaTNFα pregnancies

(N) Offspring from treated pregnancies sacrificed at day 28 post-natal. Gating strategy and representative FACS plots of heart cells based on expression of F4/80 and Ly6C. Graphs showing the proportion of various F4/80 Ly6C subpopulations and absolute number per gram of heart tissues. Proportion of MHCII, and CCR2 (%) within F4/80+Ly6C+ population from P28 hearts.

Each symbol represents an individual mouse and statistical significance was tested using one-way ANOVA with Bonferroni multiple comparison test (B-E and J,M, and N (bar graph), Brown-Forsythe test with Dunnett's multiple comparison test (F and K) or two-way ANOVA, comparing means of CCR2+ or CCR2- % between mouse groups with Tukey's multiple comparison test (I and L). ns = not significant, *$p \leq 0.05$, ** $p \leq 0.01$, ***$p \leq 0.001$ **** $p \leq 0.0001$. In all cases, data are mean ± SEM

Figure S13

A

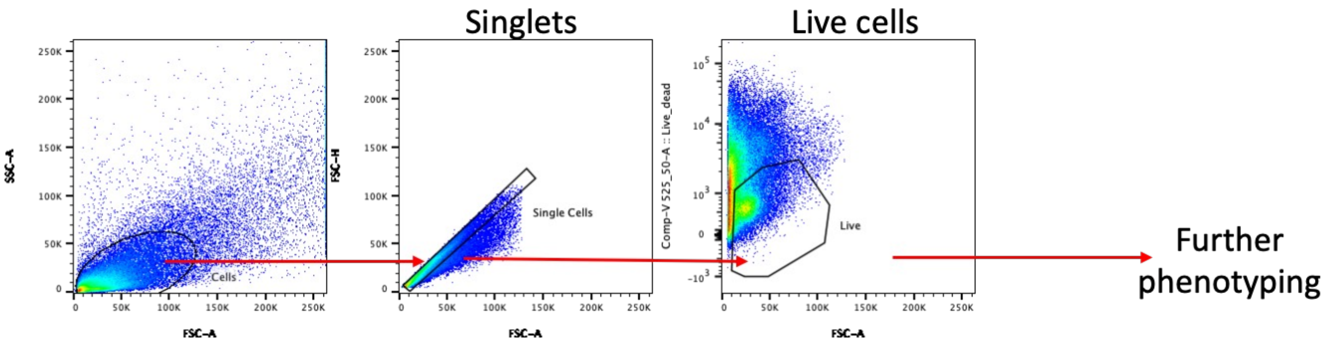

B

Primary Antibodies	Cat# and RFID	Company
Anti-Mouse CD45.1, APC Cy7 (clone: A20)	110716; RRID: AB_313505	Biolegend
Anti-Mouse CD45.2, BV605 (clone: 104)	10984; RRID:AB_2563485	Biolegend
Anti-Mouse CD45.2, PE (clone: 104)	109808; RRID:AB_313445	Biolegend
Anti-Mouse CD45, PE (clone: 30-F11)	103106; RRID:AB_312971	Biolegend
Anti-Mouse Ly-6C, PE Cy7 (clone: HK1.4)	128018; RRID:AB_1732082	Biolegend
Anti-Mouse F4/80, PE (clone: BM8)	123110; RRID:AB_893486	Biolegend
Anti-Mouse F4/80, APC (clone: BM8)	123116; RRID:AB_893481	Biolegend
Anti-Mouse F4/80, PerCP Cy5 (clone: BM8)	123128; RRID:AB_893484	Biolegend
Anti-Mouse CX3CR1, PerCP Cy5 (clone: SA011F11)	149009; RRID:AB_2564493	Biolegend
Anti-Mouse CX3CR1, APC (clone: SA011F11)	149008; RRID:AB_2564493	Biolegend
Anti-Mouse MHC II (I-Ab), FITC (clone: AF6-120.1)	116406; RRID:AB_313725	Biolegend
Anti-Mouse Mertk, AF700 (clone: DS5MMER)	56-5751-80; RRID:AB_2784770	Invitrogen
Anti-Mouse CCR2, BV421 (clone: SA203G11)	150605; RRID:AB_2571913	Biolegend
Anti-Mouse CCR6, BV605 (clone: 29-2L17)	129819; RRID:AB_2562513	Biolegend
Anti-Mouse CCR5, AF488 (clone: HM-CCR5)	107008; RRID:AB_528756	Biolegend
Anti-Mouse CXCR2, PerCP Cy5 (clone: SA044G4)	149308; RRID:AB_2565696	Biolegend
Anti-Mouse Ly-6G, PE (clone: 1A8)	127608; RRID:AB_1186099	Biolegend
Anti-Mouse Ly-6G, AF700 (clone: 1A8)	127622; RRID:AB_1064326 9	Biolegend
Anti-Mouse CD3, AF700 (clone: 17A2)	56-0032-82; RRID:AB_529507	Invitrogen
Anti-Mouse CD4, Super Bright 780 (clone: RM4-5)	78-0042-82; RRID:AB_2722967	Invitrogen
Anti-Mouse NK1.1, AF647 (clone: PK136)	108720; RRID:AB_2132713	Biolegend
Anti-Mouse CCR2, BV421 (clone: SA203G11)	150605; RRID:AB_2571913	Biolegend
Anti-Mouse IL-1β, FITC (clone: NJTEN3)	11-7114-80; RRID:AB_1071883 9	Invitrogen
Anti-Mouse IL-6, PE (clone: MP5-20F3)	504503; RRID:AB_315337	Biolegend
Anti-Mouse TMTSP (THSD1), Purified (clone: TX17.10)	122102;RRID:AB_8 30887	Biolegend
Anti-Mouse Goat anti-rat IgG, PE Cy7 (clone: Poly4054)	405413'RRID:AB_1 0661733	Biolegend
Anti-Mouse CD54 (ICAM1), PerCP Cy5.5 (clone: YN1/1.7.4)	116123; RRID:AB_2715951	Biolegend
Anti-Mouse CD102 (ICAM2), AF488 (clone: 3C4(MIC2/4))	105609; RRID:AB_2264501	Biolegend
Anti-Mouse CD31, AF700 (clone: 390)	102444;RRID:AB_2 832289	Biolegend
Anti-Mouse CD105 Endoglin, Pacific Blue (clone: MJ7/18)	120411; RRID:AB_2098890	Biolegend
Anti-Mouse VEGFR1, PE (clone: 141522)	FAB4711P; RRID:AB_2107040	R&D systems
Anti-Mouse TropT, APC (clone: 13-11)	565744; RRID:AB_2739341	BD Biosciences
Anti-Mouse CD11b, PE (clone: M1/70)	101208; RRID:AB_312791	Biolegend
Anti-Mouse CD11b, BV605 (clone: M1/70)	101257; RRID:AB_2565431	Biolegend
Anti-Mouse CD62L, PE Cy7 (clone: MEL-14)	104418; RRID:AB_313103	Biolegend
Anti-Mouse CD114, FITC (clone: 723806)	FAB6039G	R&D systems

C

Primary Antibodies	Cat#	Company
Rabbit polyclonal to CD31	ab28364	Abcam
Rat monoclonal [V.7C7.1] Endomucin	ab106100	Abcam
Monoclonal Anti-Actin, α-Smooth Muscle - FITC	F3777-.2ML	Sigma
Rat anti-mouse CD45 Antibody [30-F11]	103101	BioLegend
Wheat Germ Agglutinin Alexa Fluor 488	W11261	Thermofisher
Wheat Germ Agglutinin Alexa Fluor 647	W32466	Thermofisher
Rabbit Collagen I AF488	NBP1-77 458	Novus Bio
Collagen IV	NBP120-6586	Novus Bio
Isolectin B4	B-1205	Vector
Anti-phospho histone H3 AF647	06-570	Merck Millipore
Transgelin rabbit polyclonal	Ab14106	Abcam
WT1 rabbit polyclonal	12609-1	Proteintech
**Secondary Antibodies**	**Cat#**	**Company**
Streptavidin alexa fluor 555	S32355	Thermofisher
Donkey anti-Rat IgG (H+L) Alexa Fluor 555	A48270	Thermofisher
Donkey anti-Rabbit IgG (H+L) Alexa Fluor 647	A-31573	Thermofisher
Hoechst 33342	Ab228551	Abcam

D

Probe	Forward sequence (5' to 3')	Reverse Sequence (5' to 3')
Col 1 a1	GCTCCTCTTAGGGGCCACT	CCACGTCTCACCATTGGGG
Col 1 a2	GTAACTTCGTGCCTAGCAACA	CCTTTGTCAGAATACTGAGCAGC
Col2a1	CAGGATGCCCGAAAATTAGGG	ACCACGATCACCTCTGGGT
Col4a5	GTCCACCAGGTACAGAAGGTC	CTCCTTTCAAACCAGGTAAGCC
Col 4a6	ATCGGATACTCCTTCCTCATGC	CCAGGGGAGACTAGGGACTG
col 9A1	CGACCGACCAGCACATCAA	AGGGGGACCCTTAATGCCT
Col 9A2	AAGGGGCCTCCAGGTAAAGTT	TCCCATTAAACCATCAATGCCA
Col 9A3	GGAATGCCGGGGTTCAAGG	AGTCCTCTTAATCCTCGTGGG
Cxcl1	CTGGGATTCACCTCAAGAACATC	CAGGGTCAAGGCAAGCCTC
CXCL2	CCAACCACCAGGCTACAGG	GCGTCACACTCAAGCTCTG
CXCL5	GTTCCATCTCGCCATTCATGC	GCGGCTATGACTGAGGAAGG
CCL2	TTAAAAACCTGGATCGGAACCAA	GCATTAGCTTCAGATTTACGGGT
CCL3	TTCTCTGTACCATGACACTCTGC	CGTGGAATCTTCCGGCTGTAG
CCL4	TTCCTGCTGTTTCTCTTACACCT	CTGTCTGCCTCTTTTGGTCAG
CXCL12	TGCATCAGTGACGGTAAACCA	CACAGTTTGGAGTGTTGAGGAT
RPL32A	TTAAGCGAAACTGGCGGAAAC	TTGTTGCTCCCATAACCGATG
IL-6	TAGTCCTTCCTACCCCAATTTCC	TTGGTCCTTAGCCACTCCTTC
TNF-α	CATCTTCTCAAAATTCGAGTGACAA	TGGGAGTAGACAAGGTACAACCC
HPRT	GTAATGATCCAGTCAACGGGGGAC	CCAGCAAGCTTGCAACCTTAACCA
Il-10	AAACAAAGGACCAGCTGGAC	TTCCGATAAGGCTTGGCAAC
IL-1	AACCTGCTGGTGTGTGACGTTC	CAGCACGAGGCTTTTTTGTTGT
Gata4	CCCTACCCAGCCTACATGG	ACATATCGAGATTGGGGTGTCT
Gata6	TTGCTCCGGTAACAGCAGTG	GTGGTCGCTTGTGTAGAAGGA
Mef2c	ATGCCATCAGTGAATCAAAGGAT	GTGGTACGGTCTCCCAACT
Hey2	AAGCGCCCTTGTGAGGAAAC	GGTAGTTGTCGGTGAATTGGAC
Loxl2	ATTAACCCCAACTATGAAGTGCC	CTGTCTCCTCACTGAAGGCTC
Myh6	GCCCAGTACCTCCGAAAGTC	GCCTTAACATACTCCTCCTTGTC
Myh7	ACTGTCAACACTAAGAGGGTCA	TTGGATGATTTGATCTTCCAGGG
Nppa	GCTTCCAGGCCATATTGGAG	GGGGGCATGACCTCATCTT
Nppb	GAGGTCACTCCTATCCTCTGG	GCCATTTCCTCCGACTTTTCTC
Yap1	ACCCTCGTTTTGCCATGAAC	TGTGCTGGGATTGATATTCCGTA
Tcf21	CCCACTAAGAAAAGCCCGCTC	CCGTTCTCGTACTTGTCGTTC
Sema3d	CTGTATCCCCTTTTTGGGTTCAT	AACCAGACTGAGCAGGAAGAC

Supplementary Figure 13

(A) Flow cytometry gating strategy used for all flow cytometry data. Figure shows strategy of gating on singlet and live cells (based on live/dead stain)

(B) Flow cytometry antibody resource table

(C) Immunofluorescence antibody resource table

(D) Forward and reverse sequences of all primers used in the paper
